# A pan-variant mRNA-LNP T cell vaccine protects HLA transgenic mice from mortality after infection with SARS-CoV-2 Beta

**DOI:** 10.3389/fimmu.2023.1135815

**Published:** 2023-03-09

**Authors:** Brandon Carter, Pinghan Huang, Ge Liu, Yuejin Liang, Paulo J. C. Lin, Bi-Hung Peng, Lindsay G. A. McKay, Alexander Dimitrakakis, Jason Hsu, Vivian Tat, Panatda Saenkham-Huntsinger, Jinjin Chen, Clarety Kaseke, Gaurav D. Gaiha, Qiaobing Xu, Anthony Griffiths, Ying K. Tam, Chien-Te K. Tseng, David K. Gifford

**Affiliations:** ^1^ Computer Science and Artificial Intelligence Laboratory, Massachusetts Institute of Technology, Cambridge, MA, United States; ^2^ Department of Electrical Engineering and Computer Science, Massachusetts Institute of Technology, Cambridge, MA, United States; ^3^ Department of Microbiology and Immunology, The University of Texas Medical Branch, Galveston, TX, United States; ^4^ Acuitas Therapeutics, Vancouver, BC, Canada; ^5^ Department of Neuroscience, Cell Biology, and Anatomy, The University of Texas Medical Branch, Galveston, TX, United States; ^6^ National Emerging Infectious Diseases Laboratories, Department of Microbiology, Boston University School of Medicine, Boston, MA, United States; ^7^ Department of Pathology, The University of Texas Medical Branch, Galveston, TX, United States; ^8^ Department of Biomedical Engineering, Tufts University, Medford, MA, United States; ^9^ Ragon Institute of MGH, MIT, and Harvard, Cambridge, MA, United States; ^10^ Division of Gastroenterology, Massachusetts General Hospital, Boston, MA, United States; ^11^ Department of Biological Engineering, Massachusetts Institute of Technology, Cambridge, MA, United States

**Keywords:** COVID-19, SARS-CoV-2, peptide vaccine, mRNA-LNP, challenge study, T cell vaccine

## Abstract

Licensed COVID-19 vaccines ameliorate viral infection by inducing production of neutralizing antibodies that bind the SARS-CoV-2 Spike protein and inhibit viral cellular entry. However, the clinical effectiveness of these vaccines is transitory as viral variants escape antibody neutralization. Effective vaccines that solely rely upon a T cell response to combat SARS-CoV-2 infection could be transformational because they can utilize highly conserved short pan-variant peptide epitopes, but a mRNA-LNP T cell vaccine has not been shown to provide effective anti-SARS-CoV-2 prophylaxis. Here we show a mRNA-LNP vaccine (MIT-T-COVID) based on highly conserved short peptide epitopes activates CD8^+^ and CD4^+^ T cell responses that attenuate morbidity and prevent mortality in HLA-A*02:01 transgenic mice infected with SARS-CoV-2 Beta (B.1.351). We found CD8^+^ T cells in mice immunized with MIT-T-COVID vaccine significantly increased from 1.1% to 24.0% of total pulmonary nucleated cells prior to and at 7 days post infection (dpi), respectively, indicating dynamic recruitment of circulating specific T cells into the infected lungs. Mice immunized with MIT-T-COVID had 2.8 (2 dpi) and 3.3 (7 dpi) times more lung infiltrating CD8^+^ T cells than unimmunized mice. Mice immunized with MIT-T-COVID had 17.4 times more lung infiltrating CD4^+^ T cells than unimmunized mice (7 dpi). The undetectable specific antibody response in MIT-T-COVID-immunized mice demonstrates specific T cell responses alone can effectively attenuate the pathogenesis of SARS-CoV-2 infection. Our results suggest further study is merited for pan-variant T cell vaccines, including for individuals that cannot produce neutralizing antibodies or to help mitigate Long COVID.

## Introduction

Current strategies for COVID-19 vaccine design utilize one or more SARS-CoV-2 Spike protein subunits to primarily activate the humoral arm of the adaptive immune response to produce neutralizing antibodies to the Spike receptor binding domain (RBD) (1, 2). Vaccination to produce neutralizing antibodies is a natural objective, as neutralizing antibodies present an effective barrier to the viral infection of permissive cells by binding to the RBD and thus blocking cellular entry *via* the ACE2 receptor. However, the strategy of focusing on Spike as the sole vaccine target has proven problematic as Spike rapidly evolves to produce structural variants that evade antibody-based acquired immunity from vaccination or infection with previous viral variants (3, 4). Compared to the original Wuhan variant, novel viral variants are arising that are more contagious (5), and infectious to a broader range of host species (6). Thus, vaccine designers are pursuing a stream of novel Spike variant vaccines. Multivalent Spike vaccines and bivalent booster vaccines provide protection against multiple known variants of concern (VOCs) of SARS-CoV-2 but are not necessarily protective against unknown future variants (7). Mosaic RBD nanoparticles that display disparate SARS-CoV RBDs have been found to produce effective neutralizing antibodies against both SARS-CoV-1 and SARS-CoV-2 (8), but the robustness of mosaic RBD protection against possible future Spike mutations depends upon conserved Spike structural epitopes.

The vaccine approach we present depends upon conserved T cell epitopes drawn from the entire viral proteome for protection against future variants. Since T cell epitopes can originate from any part of the viral proteome, they can be drawn from portions of the proteome that are evolutionarily stable and immunogenic. The predication of epitope stability can be accomplished by historical analysis of thousands of viral variants (9), structural analysis (10), or the functional analysis of mutations lethal to the virus. We have used a set of highly stable epitope candidates to design a T cell vaccine that covers a broad range human MHC class I and class II haplotypes. Our T cell vaccine design proceeds by vaccine epitope selection that optimizes population coverage where every vaccinated individual is predicted to experience on average multiple immunogenic peptide-HLA hits (11, 12).

## Methods

### *n-times* coverage vaccine design

**Figure 1:**
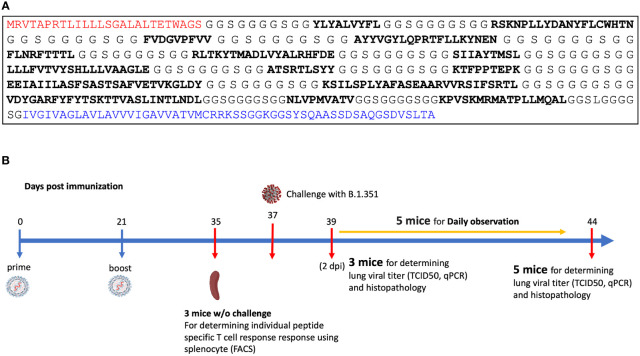
**(A)** Assembled vaccine construct containing a secretion signal sequence (red), peptides (bold) joined by non-immunogenic glycine/serine linkers, and an MHC class I trafficking signal (blue). **(B)** Study design.

The MIT-T-COVID vaccine realizes an *n-times* coverage objective by encoding multiple epitopes for each target MHC class I and II diplotype to (1) expand diverse sets of T cell clonotypes to fight viral infection, (2) accommodate variations in epitope immunogenicity between vaccinees, and (3) reduce the chances that viral evolution will lead to immune system escape (12). The MIT-T-COVID vaccine consists of eight MHC class I epitopes and three MHC class II epitopes (**Figure 1A**; **Supplementary Methods** and **Supplementary Table 1**). The MHC class I and II vaccine peptides are encoded into a single mRNA construct for delivery with the same Acuitas LNP delivery platform that is used by the Pfizer-BioNTech Comirnaty^®^ vaccine (**Figure 1A**). The eight MIT-T-COVID MHC class I epitopes are the HLA-A*02:01 subset of the MHC class I *de novo* MIRA only vaccine design of Liu et al. (11) that used combinatorial optimization to select vaccine epitopes to maximize *n-times* population coverage over HLA haplotype frequencies (12). For inclusion in the assembled construct, the eight MHC class I vaccine peptides were randomly shuffled, and alternate peptides were flanked with five additional amino acids at each terminus as originally flanked in the SARS-CoV-2 proteome. Selected epitopes were flanked to test if flanking enhanced or impaired epitope presentation. The three MHC class II epitopes were selected by considering all SARS-CoV-2 proteome windows of length 13-25 and selecting conserved peptides that are predicted to be displayed by the mouse MHC class II allele H-2-IAb (**Supplementary Methods**). The mRNA construct encodes a secretion signal sequence at the N-terminus and an MHC class I trafficking signal (MITD) at the C-terminus (13). Peptide sequences are joined by non-immunogenic glycine/serine linkers (14). The construct also included control peptides for HLA-A*02:01 and H-2-IAb (CMV pp65: NLVPMVATV for HLA-A*02:01 and Human CD74: KPVSKMRMATPLLMQAL for H-2-IAb) (15).

### SARS-CoV-2 Beta variant challenge study

We immunized with three test articles: a negative control injection (PBS), the Pfizer-BioNTech Comirnaty^®^ vaccine (wastage that was refrozen and then thawed), and the MIT-T-COVID vaccine (**Figure 1B**). We immunized HLA-A*02:01 human transgenic mice with these three vaccines, immunizing 11 age-matched male mice with each vaccine. Comirnaty^®^ or MIT-T-COVID immunizations contained of 10 µg of mRNA. The mice were immunized at Day 0 and boosted at Day 21 (**Supplementary Methods**). At Day 35 three mice from each group were sacrificed for immunogenicity studies, and at Day 37 the remaining eight mice were challenged intranasally (I.N.) with 5 x 10^4^ TCID_50_ of the SARS-CoV-2 B.1.351 variant. Challenged mice were subjected to daily monitoring for the onset of morbidity (i.e., weight changes and other signs of illness) and any mortality. At Day 39 (2 days post infection) three mice were taken from each group and sacrificed to determine viral burdens and to perform lung histopathology. At Day 44 (7 days post infection) the remaining mice were sacrificed to determine viral burdens and to perform lung histopathology.

Additional methods are presented in the **Supplementary Material**.

## Results

### MIT-T-COVID vaccine expands CD8^+^ and CD4^+^ SARS-CoV-2 specific T cells

**Figure 2:**
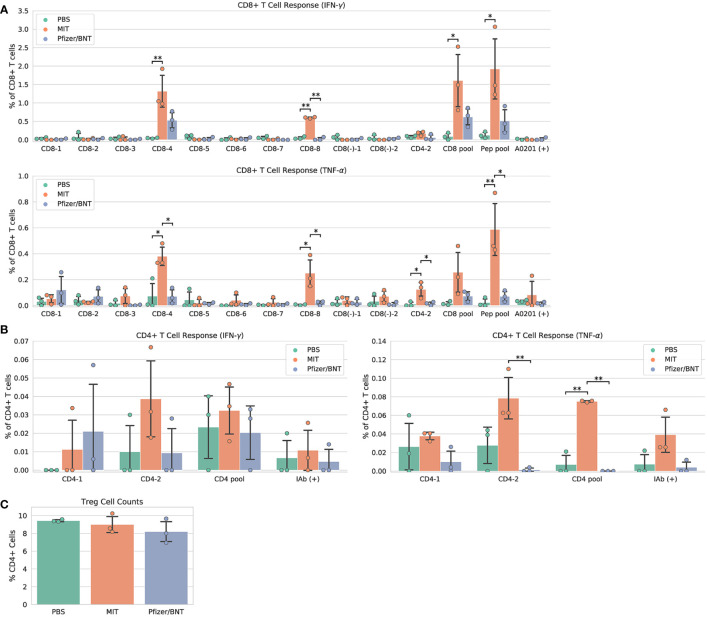
Vaccine immunogenicity. **(A)** CD8^+^ T cell responses, **(B)** CD4^+^ T cell responses, **(C)** Foxp3^+^ regulatory T cells (Tregs) as a percentage of all CD4^+^ cells. The CD8 pool includes MHC class I peptides CD8-1—CD8-8 (**Supplementary Table 1**). The CD4 pool includes MHC class II peptides CD4-1 and CD4-2. The Pep pool includes all query peptides in **Supplementary Table 1** except CD4-3. Error bars indicate the standard deviation around each mean. *P* values were computed by one-way ANOVA with Tukey’s test. *P < 0.05, **P < 0.01. See also **Supplementary Figure 12**.

Immunization with MIT-T-COVID vaccine expanded CD8^+^ and CD4^+^ T cells that expressed interferon gamma (IFN-γ) or tumor necrosis factor alpha (TNF-α) when queried by vaccine epitopes (**Figures 2A, B**). The observed variability of immunogenicity of MIT-T-COVID epitopes in convalescent COVID patients (**Supplementary Table 1**) and in the present study supports our usage of multiple epitopes per MHC diplotype for *n-times* coverage. Immunization by Comirnaty^®^ produced no significant T cell responses to vaccine epitopes (including a Spike epitope) when compared to PBS. CD8^+^ T cells that are activated by the CD8-4 epitope (YLQPRTFLL, Spike 269-277) are expanded in animals immunized with the MIT-T-COVID vaccine (IFN-γ 1.32% ± 0.53% of CD8^+^ T cells, *P* = 0.0087 *vs*. PBS; TNF-α 0.38% ± 0.09% of CD8^+^ T cells, *P* = 0.0149 *vs*. PBS; **Figure 2A**). Similarly, the CD8-8 epitope (TVYSHLLLV, ORF3a 89-97) activated CD8^+^ T cells that are expanded by the MIT-T-COVID vaccine (IFN-γ 0.60% ± 0.02% of CD8^+^ T cells, *P* = 0.001 *vs*. PBS; TNF-α 0.25% ± 0.12% of CD8^+^ T cells, *P* = 0.015 *vs*. PBS) and the CD4-2 epitope (RFYFYTSKTTVASLIN, ORF1ab 1421-1436) activated CD4^+^ T cells are expanded by the MIT-T-COVID vaccine (TNF-α 0.078% ± 0.027%, *P* = 0.058 *vs*. PBS, *P* = 0.010 *vs*. Comirnaty^®^, IFN-γ not significant, **Figure 2B**). We also measured interleukin-2 (IL-2) expression and found the MIT-T-COVID vaccine significantly expanded CD4^+^ T cells activated by the SARS-CoV-2 CD4^+^ pool (*P* = 0.0015 *vs*. PBS, *P* = 0.0013 *vs*. Comirnaty^®^, **Supplementary Figure 12**). The lack of HLA-A*02:01 transgenic animal response to certain CD8^+^ epitopes that were immunogenic in patients (**Supplementary Table 1**) is consistent with past studies of transgenic mouse models (16), the variability of immunogenicity of epitopes between in-bred mice (17), and the potential of immunodominance of the immunogenic epitopes.

We immunized an additional cohort of female HLA-A*02:01 transgenic mice (**Supplementary Methods**), and results are shown in **Supplementary Figure 8**. We also immunized female mice with synthetic peptides mixed with poly IC adjuvant and found no significant SARS-CoV-2 specific T cell responses compared to PBS controls (**Supplementary Figures 9A, B** and **Supplementary Methods**). We found a significant increase in the number of effector and memory CD8^+^ CD44^+^ T cells in MIT-T-COVID and Comirnaty^®^-immunized mice, compared to those immunized with Peptide/poly IC or PBS (**Supplementary Figure 9C**).

T cells that lack IFN-γ responses can produce effective immune mediators (18). We found that the fraction of CD4^+^ T cells that are Foxp3^+^, designated regulatory T cells (Treg), were not expanded in Comirnaty^®^ or MIT-T-COVID-immunized animals, suggesting that Treg cells that could induce tolerance were not expanded by immunization (**Figure 2C**).

### MIT-T-COVID attenuates morbidity and prevents mortality

**Figure 3:**
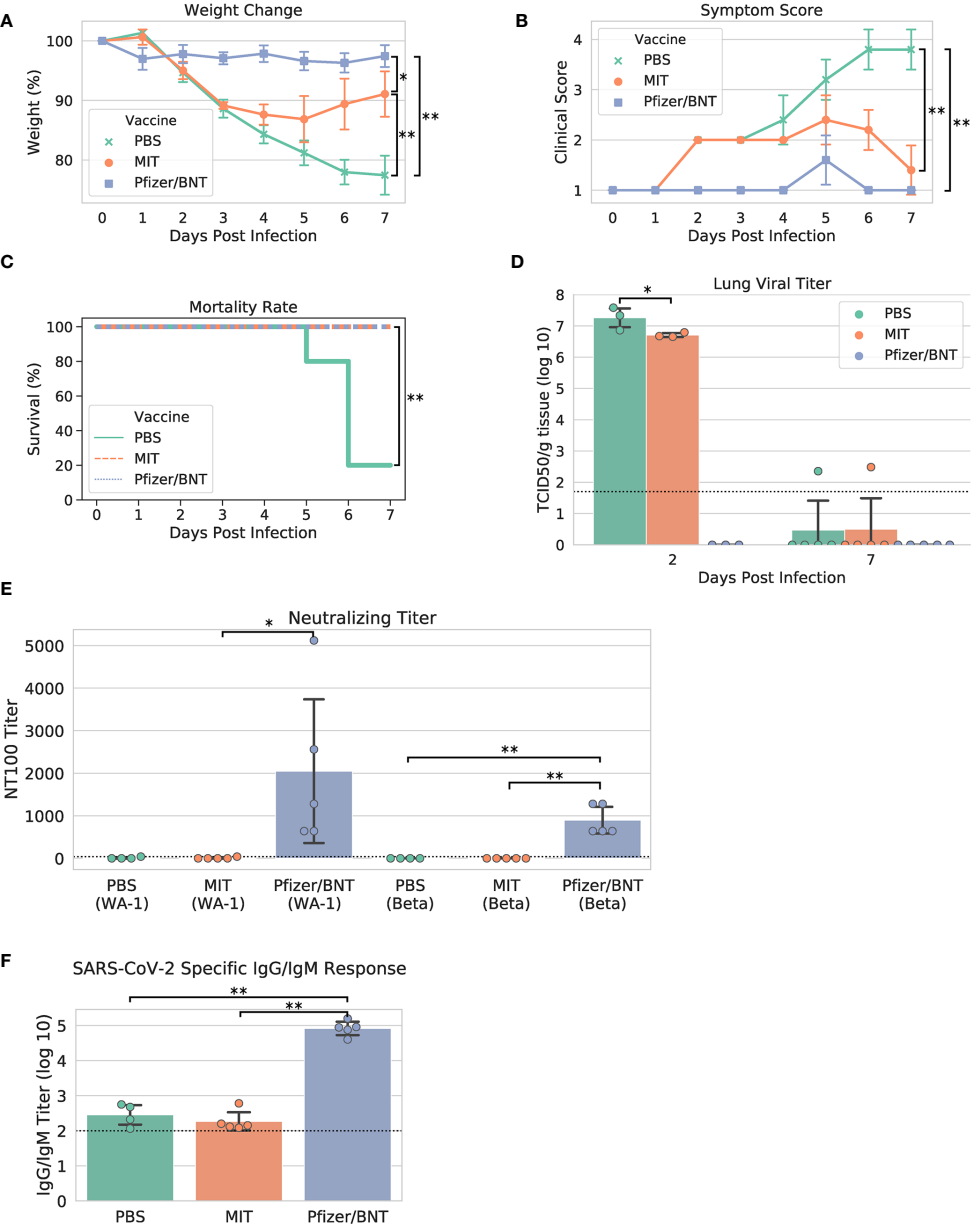
Study phenotypic data, lung viral titer, and vaccine antibody responses. **(A)** Weights *vs*. days post infection, **(B)** clinical scores *vs*. days post infection, **(C)** Kaplan-Meier mortality curve (mortality at 80% weight loss), **(D)** lung viral titer, **(E)** maximum serum dilution that provided 100% neutralization of viral infection *in vitro*, **(F)** IgG/IgM titer measured by ELISA against cell lysate infected with WA-1 SARS-CoV-2. Dotted lines in **(D–F)** indicate assay limits of detection. Error bars indicate the standard deviation around each mean. *P* values were computed by one-way ANOVA with Tukey’s test except **(C)** *P* values were computed using the logrank test. *P < 0.05 and **P < 0.01.

Upon viral challenge, both PBS and MIT-T-COVID-immunized animals exhibited a more than 10% weight loss by Day 3 (PBS mean weight reduction 11.386% ± 1.688%, MIT mean weight reduction 10.851% ± 0.641%), with the MIT-T-COVID-immunized animals beginning to recover from Day 4 onward (**Figure 3A**). The weight phenotype was mirrored by the clinical score phenotype, with PBS animals not recovering and MIT-T-COVID-immunized animals improving from Day 5 onward (**Figure 3B** and **Supplementary Methods**). The Comirnaty^®^ vaccine protected animals from significant weight loss and poor clinical scores.

When the yields of infectious progeny virus were measured at 2 days post infection (dpi), we noted that mice immunized with MIT-T-COVID vaccine had 6.706 ± 0.076 log10 TCID_50_/g, compared to 7.258 ± 0.367 log10 TCID_50_/g in the PBS control, representing a moderate 3.6-fold reduction in viral replication (*P* = 0.046; **Figure 3D**). In contrast, mice immunized with Comirnaty^®^ had infectious viral titers that were below the detection limits at 2 dpi. Infectious viral titer was not significant for all test articles at 7 dpi (**Figure 3D**). Lung viral mRNA levels measured by qPCR followed the same trends as infectious virus progeny in the lungs, with Comirnaty^®^ showing no significant levels. Despite a reduced content of total and sub-genomic viral RNAs associated with MIT-T-COVID vaccine samples as assessed by qPCR, the reduction was insignificant, compared to those of PBS control (**Supplementary Figure 1**).

All the Comirnaty^®^ and MIT-T-COVID-immunized mice survived to 7 dpi, when the study was terminated, with the difference in clinical scores of these two vaccine groups becoming insignificant (**Figure 3C**). In contrast, four of five PBS control mice had been euthanized because of weight loss > 20%. The survival of all five animals immunized with Comirnaty^®^ and the MIT-T-COVID, respectively, was significant, compared to that of PBS-immunized control (*P* = 0.0053, logrank test).

We noted that mice immunized with the Comirnaty^®^ vaccine elicited substantial specific antibodies capable of neutralizing Beta and WA-1 variants of SARS-CoV-2 with 100% neutralizing titers (NT_100_) of 896 ± 350 and fold 2048 ± 1887, respectively (**Figure 3E**). The higher neutralizing titer against WA-1 is expected as it is the strain matched to Comirnaty^®^. As expected for a peptide-based vaccine, no neutralizing titer could be readily detected in the serum from mice immunized with MIT-T-COVID vaccine or PBS. Total specific IgG/IgM antibodies were also measured by ELISA against a cell lysate prepared from WA-1-infected Vero E6 cells. Comirnaty^®^ has a log10 IgG/IgM titer of 4.915 ± 0.213, while the log10 titer produced by PBS was 2.452 ± 0.321 and the MIT-T-COVID vaccine produced a log10 titer of 2.266 ± 0.291 (**Figure 3F**). The low but detectable titers in PBS and MIT-T-COVID vaccine-immunized mice may represent an early IgM response to viral infection.

### MIT-T-COVID increases T cell infiltration of infected lungs

All lung samples were subjected to immunohistochemistry (IHC) staining for the SARS-CoV-2 spike protein (**Supplementary Figure 2**). We found specimens immunized with PBS exhibited extensive staining indicative of viral infection throughout the epithelium of both the bronchioles and the alveolar sacs, with the viral infection appearing more intense at 2 dpi. Although viral infection is significantly reduced by 7 dpi, viral antigen was still readily detectable throughout alveoli. In comparison, specimens immunized with the MIT-T-COVID vaccine exhibited similarly extensive viral infection at 2 dpi throughout the bronchiolar and alveolar epithelia, albeit somewhat reduced in intensity. However, by 7 dpi, viral infection was significantly reduced in both extent and intensity, with brown puncta being detected only in a few alveoli scattered throughout the tissue. Contrasted with both PBS and MIT-T-COVID-immunized specimens, the Comirnaty^®^-immunized specimens exhibited significantly reduced viral infections at both 2 and 7 dpi. Apart from a single area at 7 dpi (see **Supplementary Figure 3**), viral antigen was undetected at both timepoints in Comirnaty^®^-immunized animals.

Paraffin-embedded and H&E-stained lung specimens of differentially immunized mice, harvested at 2 and 7 dpi, were subjected to histopathological examination (**Supplementary Figure 4**). We found that at 7 dpi mice immunized with MIT-T-COVID vaccine exhibited extensive lymphocytic infiltrations in perivascular regions and spaces from around bronchi, bronchioles, to alveoli. Fewer infiltrations were found in mice immunized with either Comirnaty^®^ or PBS. Additionally, these infiltrations only localized at perivascular regions around bronchi and large bronchioles. Despite the less intensive and localized inflammatory infiltrates, we also noted widespread congestion, hemorrhage, and few foci of thromboembolism were exclusively observed within the lungs of Comirnaty^®^-immunized mice but not others (**Supplementary Figure 4**). We also noted the lung histopathology was milder but the same pattern at 2 dpi than those of 7 dpi (data not shown).

**Figure 4:**
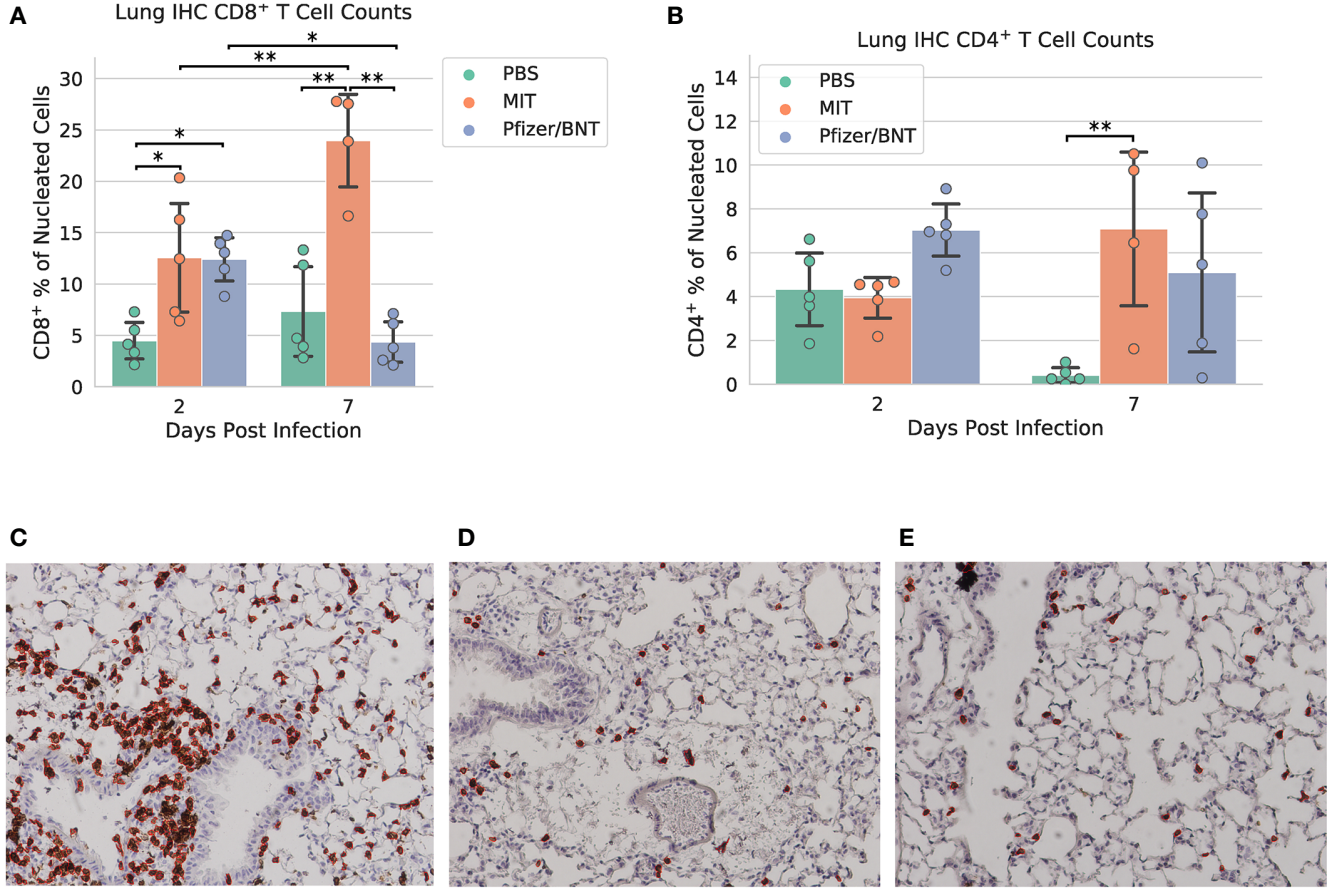
Lung immunohistochemistry for CD8^+^ and CD4^+^ cells. Counts of **(A)** CD8^+^ and **(B)** CD4^+^ T cells expressed as a percentage of all nucleated cells visible in each field from lung tissue. Example CD8^+^ stain images at 7 dpi for **(C)** MIT-T-COVID, **(D)** Pfizer/BNT, and **(E)** PBS-immunized animals. Lung samples were subjected to IHC staining for CD8 (brown) with hematoxylin counterstain (blue). Images were taken at 10x magnification. Red outlines in **(C-E)** indicate CD8^+^ cells identified and counted by CellProfiler software (**Supplementary Methods**). Error bars indicate the standard deviation around each mean. *P* values were computed by two-way ANOVA with Tukey’s test. *P < 0.05 and **P < 0.01. See also **Supplementary Figures 5–7**.

Lung specimens were subjected to IHC staining for CD8^+^ and CD4^+^ cells at both 2 and 7 dpi (**Figure 4** and **Supplementary Figures 5–7**). At 2 dpi, we found a significant increase in CD8^+^ T cells infiltrating the lungs in mice immunized with MIT-T-COVID (12.6% ± 5.91% of all nucleated cells were CD8^+^) or Comirnaty^®^ (12.4% ± 2.35%) compared to mice immunized with PBS (PBS mean 4.48% ± 1.99%; *P* = 0.044 *vs*. MIT-T-COVID, *P* = 0.050 *vs*. Comirnaty^®^; **Figure 4A**). At 7 dpi, we found a significant increase in CD8^+^ T cells infiltrating the lungs in mice immunized with MIT-T-COVID (24.0% ± 5.21% of all nucleated cells were CD8^+^) compared to mice immunized with Comirnaty^®^ (Comirnaty^®^ mean 4.35% ± 2.20%, *P* = 0.001) or PBS (PBS mean 7.32% ± 4.88%, *P* = 0.001; **Figure 4A**). We observed a significant increase in CD8^+^ T cells infiltrating the lungs in mice immunized with MIT-T-COVID between 2 and 7 dpi (*P* = 0.0039), and a significant decrease in CD8^+^ T cells infiltrating the lungs in mice immunized with Comirnaty^®^ between 2 and 7 dpi (*P* = 0.044; **Figure 4A**). At7 dpi, we also found a significant increase in CD4^+^ T cells infiltrating the lungs in mice immunized with MIT-T-COVID (7.09% ± 4.05%) compared to mice immunized with PBS (PBS mean 0.41% ± 0.39%; *P* = 0.0062; **Figure 4B**). In the unchallenged cohort of female mice, we found an increase in CD8^+^ T cells infiltrating the lungs in mice immunized with MIT-T-COVID (1.12% ± 0.59% of all nucleated cells were CD8^+^) or Comirnaty^®^ (0.87% ± 0.56%) compared to mice immunized with PBS (0.09% ± 0.10%; *P* = 0.001 *vs*. MIT-T-COVID, *P* = 0.003 *vs*. Comirnaty^®^; **Supplementary Figures 10, 11**).

## Discussion

Here we find that a T cell vaccine (“MIT-T-COVID”) that contains the human HLA-A*02:01 displayed subset of our COVID-19 T cell vaccine and additional mouse specific CD4^+^ epitopes provides effective prophylaxis against the onset of SARS-CoV-2-induced morbidity and mortality caused by SARS-CoV-2 Beta infection in transgenic mice carrying HLA-A*02:01. The vaccine consists of 11 short T cell epitopes that are unchanged over 22 presently known SARS-CoV-2 variants of concern (VOCs) (**Supplementary Methods**). We further demonstrate the MIT-T-COVID vaccine causes significant infiltration of CD8^+^ and CD4^+^ T lymphocytes in the lungs post infection. We chose the Beta variant for our challenge study as it is more pathogenic than recent variants (19) and its host range includes wild type mice (6) since murine infection with SARS-CoV-2 variants that require ectopic human ACE2 expression results in fatal encephalitis (20) which is not ideal for evaluating the efficacy of a T cell vaccine. We selected a highly pathogenic variant that models human disease to evaluate the effectiveness of our T cell vaccine in anticipation of possible future SARS-CoV-2 variants that result in severe disease. MIT-T-COVID vaccine epitopes delivered as peptides with a Poly(I:C) adjuvant failed to induce significant immune responses, supporting the value of mRNA-LNP delivery of the epitopes.

Existing Spike vaccines produce a T cell response that is thought to be important for vaccine effectiveness and durability (21). However, the T cell response induced by a Spike vaccine may be less effective in promoting durable immune responses than the T cell response induced by a pure T cell vaccine. Spike based T cell responses may contain epitopes that are subject to evolutionary change (22, 23) that dominate stable epitopes, or Spike epitopes may not be as well presented or immunogenic as epitopes drawn from a more diverse set of SARS-CoV-2 proteins. Thus, T cell augmentation strategies for Spike vaccines (11) and pure T cell vaccines will require further exploration to unravel the hierarchy of immune responses to their components, and how to structure a vaccine for optimal prophylaxis. In patients with impaired antibody responses, T cell vaccines would eliminate the burden of non-immunogenic B cell epitopes (24) and provide immune protection, at least to a certain extent, against infection.

Multiple designs for T cell vaccines for SARS-CoV-2 have been proposed (9, 10, 25, 26) and are in clinical trials (NCT05113862, NCT0488536, NCT05069623, NCT04954469), but identifying the mechanisms behind the efficacy of pure T cell vaccines remains an open question. Substantial literature suggests that T cell responses are integral to the adaptive immunity to COVID-19 (21, 27). For example, a study that ablated the B cell compartment of the immune system in Spike immunized mice found that CD8^+^ T cells alone can control viral infection (28). Pardieck et al. (26) found that vaccination with a single mouse restricted CD8^+^ T cell epitope conferred protection against mortality from the Leiden-0008/2020 SARS-CoV-2 variant (B.1) in K18-hACE2 transgenic mice, but unlike our study, required three doses for efficacy, did not engage a CD4^+^ T cell response, did not identify significant T cell infiltration of the lungs, challenged with a lower viral dose (5000 PFU *vs*. our 5 x 10^4^ TCID_50_), and used a variant of SARS-CoV-2 that is not pathogenic in wild type mice (6). In addition to specific antibody responses, COVID-19 vaccinees and convalescent patients possess SARS-CoV-2 specific CD8^+^ and CD4^+^ T cells, suggesting the contribution of the T cell compartment to the adaptive immunity to COVID-19 (29), and clinical findings have revealed vaccine-induced T cell responses in B cell-deficient patients (30). It has also been reported that vaccination by WA (Wuhan) Spike in a mouse model failed to produce antibodies fully capable of neutralizing the SA (Beta) variant of SARS-CoV-2, yet immunized mice were protected against Beta strain challenge (31). In addition, vaccination with T cell epitope-rich Nucleocapsid protein produced specific T cell responses thought to be causally associated with viral control (32). Intranasal vaccination of mice with SARS-CoV-1 Nucleocapsid followed by challenge with 10^4^ Plaque-forming units of SARS-CoV-1 prevented mortality in 75% of the mice (33). Combined Spike and Nucleocapsid vaccination improved viral control compared to Spike vaccination alone in preclinical models, while CD8^+^ T cell depletion demonstrated the role of CD8^+^ T cells in viral control and protection from weight loss (34).

The marginal IgG/IgM antibody titers that were not neutralizing elicited by mice immunized with the MIT-T-COVID vaccine indicates that the protective mechanism of the vaccine was likely based on a T cell response to the virus. The reduction in viral titer on day 2 shows that T cell responses were present early in infection. In the absence of neutralizing antibodies these responses were sufficient to rescue the immunized mice from the onset of mortality.

We expect that a T cell vaccine would provide prophylaxis, at least to certain extent, like what we have described against future SARS-CoV variants and strains that conserve the vaccine’s epitopes. We chose Beta (B.1.351) for our challenge study as it has a severe phenotype in a wild-type mouse background (6). Transgenic human ACE2 (hACE2) mice exhibit encephalitis post COVID infection that does not represent human pathology (20) and thus might not be an ideal model for assessing the efficacy of T cell-based vaccines. Instead, we chose to evaluate the HLA-A*02:01 component of our vaccine design in a HLA-A*02:01 transgenic animal model given the predominance of HLA-A02 in the human population (35).

The MIT-T-COVID vaccine induced a response where circulating T cells migrated rapidly and efficiently into the lung upon viral challenge, as evidenced by more intense and widespread lymphocytic infiltrations than other groups. Further, the degree of CD8^+^ T cell infiltration of the lungs significantly increased in mice immunized with the MIT-T-COVID vaccine between days 2 and 7 post infection and compared to that elicited by unimmunized and challenged cohorts, thereby thwarting the concern over the involvement of resident T cells of the lung.

Another finding is that widespread congestion, hemorrhage, and few foci of thromboembolism were exclusively observed within the lungs of Comirnaty^®^-immunized mice. Although this finding is unrelated to the MIT-T-COVID vaccine, it suggests the possibility of immunization-induced side-effects or immunopathology of SARS-CoV-2 vaccination and is consistent with previous reports in humans (36, 37). The exact mechanism of pulmonary embolism in this case remains currently unknown and should be explored.

Cytotoxic T cells (CD8^+^ T cells) can kill virally infected cells, and thus T cell vaccines might promote the long-term immunity to more effectively control the Long COVID. Post-acute sequelae of COVID-19 (PASC, “Long COVID”) causes persistent symptoms in ~10% of people past twelve weeks of COVID infection (38). Long COVID has been associated with the continued presence of Spike in the blood, suggesting that a tissue based viral reservoir remains in Long COVID patients (39).

Our results suggest that if one goal of a vaccine is protection against novel viral strains it may be appropriate to develop vaccines that permit symptomatic infection but protect against severe illness. Current regulatory criteria for licensing vaccines are solely based upon the prevention of symptomatic illness for vaccine matched viral strains. Further research on T cell vaccines may reveal novel vaccine designs that prevent severe illness while providing protection against viral variants as an additional tool in the fight against global pandemics.

## Data availability statement

The original contributions presented in the study are included in the article/**Supplementary Material**. Further inquiries can be directed to the corresponding authors. Original data collected during this study have been deposited to Mendeley Data: http://dx.doi.org/10.17632/p4c823jzxz.1. Source code is available on GitHub: https://github.com/gifford-lab/MIT-T-COVID-vaccine.

## Ethics statement

Animal studies were conducted at Galveston National Laboratory at University of Texas Medical Branch at Galveston, Texas, based on a protocol approved by the Institutional Animal Care and Use Committee at UTMB at Galveston.

## Author contributions

Conceptualization: BC, GL, DG. Methodology: BC, GL, PH, C-TT, PL, YT, DG. Investigation: BC, PH, GL, YL, B-HP, LM, AD, JH, VT, PS-H, JC, CK, C-TT, DG. Visualization: BC, PH. Funding acquisition: DG. Project administration: C-TT, DG. Supervision: C-TT, YT, GG, AG, QX, DG. Writing – original draft: DG, PH, BC. Writing – review and editing: PH, BC, GG, AG, C-TT, YT, DG. All authors contributed to the article and approved the submitted version.
