# Supplementary material for: A pan-variant mRNA-LNP T cell vaccine protects HLA transgenic mice from mortality after infection with SARS-CoV-2 Beta
Source: Front Immunol. 2023 Mar 9;14:1135815. doi: 10.3389/fimmu.2023.1135815 (PMC10033589; doi:10.3389/fimmu.2023.1135815)
Supplement: Supplementary file 1 [file DataSheet_1.pdf]

frontiers

Supplementary Material

A pan-variant mRNA-LNP T cell vaccine protects HLA transgenic mice from mortality after infection with SARS-CoV-2 Beta

Brandon Carter[+], Pinghan Huang[+], Ge Liu, Yuejin Liang, Paulo J. C. Lin, Bi-Hung Peng, Lindsay G. A. McKay, Alexander Dimitrakakis, Jason Hsu, Vivian Tat, Panatda Saenkham-Huntsinger, Jinjin Chen, Clarety Kaseke, Gaurav D. Gaiha, Qiaobing Xu, Anthony Griffiths, Ying K. Tam, Chien-Te K. Tseng[*], David K. Gifford[*]

[+] These authors have contributed equally to this work

*** Correspondence:** sktseng@utmb.edu, gifford@mit.edu

1 Supplementary Methods

1.1 Mice

A total of 33 male HLA-A*02:01 human transgenic mice (Taconic 9659) were used that were between 12 and 16 weeks old when they received their initial immunization. These CB6F1 background mice carry the MHC class I alleles HLA-A*02:01, H-2-Kb, H-2-Db, H-2-Kd, and H-2-Dd, and H-2-Ld. The mice carry the MHC class II alleles H-2-IAd, H-2-IEd, and H-2-IAb. We also immunized a female cohort of the same transgenic mouse strain (Taconic 9659, 3 mice per vaccine) for immunogenicity measurement, and results are shown in Supplementary Figures 8-11.

1.2 Tissue culture and virus

Vero E6 cells (ATCC, CRL:1586) were grown in minimum essential medium (EMEM, Gibco) supplemented with penicillin (100 units/mL), streptomycin (100 μg/mL) and 10% fetal bovine serum (FBS). Two strains of SARS-CoV-2 were used in this study, SARS-CoV-2 (US_WA-1/2020 isolate) and Beta (B.1.351/SA, Strain: hCoV-19/USA/MD-HP01542/2021). Both viruses were propagated and quantified in Vero E6 cells and stored at -80 °C until needed.

1.3 MIT-T-COVID vaccine design

MHC Class I epitopes. Eight peptides from SARS-CoV-2 were selected as a subset of the MHC class I *de novo* MIRA only vaccine design of Liu et al., 2021. We filtered this set of 36 peptides to the 8 peptides predicted to be displayed by HLA-A*02:01 by a combined MIRA and machine learning model of peptide-HLA immunogenicity (Liu et al., 2021). The combined model predicts which HLA molecule displayed a peptide that was observed to be immunogenic in a MIRA experiment, and uses machine learning predictions of peptide display for HLA alleles not observed or peptides not tested in MIRA data. Thus, all eight MHC class I peptides in our vaccine were previously observed to be immunogenic in data from convalescent COVID-19 patients (Snyder et al., 2020). We further validated that all peptides are predicted to bind HLA-A*02:01 with high (less than or equal to 50 nM) affinity

using the NetMHCpan-4.1 (Reynisson et al., 2020) and MHCflurry 2.0 (O'Donnell et al., 2020) machine learning models. For inclusion in the assembled construct, the eight vaccine peptides were randomly shuffled, and alternate peptides were flanked with five additional amino acids at each terminus as originally flanked in the SARS-CoV-2 proteome. The CD4-2 vaccine epitope produced a $CD8^+$ response, and this may be a consequence of a contained MHC class I epitope, with candidates including SLINTLNDL (HLA-A*02:01 predicted binding 55 nM), ASLINTLNDL (H-2-Db predicted binding 289 nM), FYTSKTTVASL (H-2-Kd predicted binding 293 nM), FYFYTSKTTV (H-2-Kd predicted binding 40 nM), and YFYTSKTTV (H-2-Kd predicted binding 56 nM).

MHC Class II epitopes. Three peptides from SARS-CoV-2 were optimized for predicted binding to H-2-IAb. We scored all SARS-CoV-2 peptides of length 13-25 using the sliding window approach of Liu et al. (2020) and a machine learning ensemble that outputs the mean predicted binding affinity (IC50) of NetMHCIIpan-4.0 (Reynisson et al., 2020) and PUFFIN (Zeng and Gifford, 2019). We selected the top three peptides by predicted binding affinity using a greedy selection strategy with a minimum edit distance constraint of 5 between peptides to avoid selecting overlapping windows. All three peptides were flanked with an additional five amino acids per terminus from the SARS-CoV-2 proteome.

The start and end amino acid positions of each vaccine peptide in its origin gene is shown in Supplementary Table 1. For SARS-CoV-2, peptides are aligned to reference proteins in UniProt (UniProt Consortium, 2019) (UniProt IDs: P0DTC2 (S), P0DTC3 (ORF3a), P0DTC5 (M), P0DTC9 (N), P0DTD1 (ORF1ab)). All of the epitopes are conserved over these variants of concern: Alpha (B.1.1.7), Beta (B.1.351), Gamma (P.1), Delta (21A, 21J, B.1.617.2), Kappa (B.1.617.1), Epsilon (B.1.427, B.1.429), Iota (B.1.526), Lambda (C.37), Mu (B.1.621) , Omicron (BA.1, BA.2, BA.4, BA.5, BA.2.12.1, BA.2.75, BQ.1, XBB, XBB.1.5), EU1 (B.1.177).

1.4 MIT-T-COVID vaccine formulation

Codon optimization for mouse expression of the MIT-T-COVID vaccine construct was performed using the IDT Codon Optimization Tool (Integrated DNA Technologies). The resulting nucleic acid sequence is provided in Supplementary Table 2. RNA was synthesized by TriLink BioTechnologies as a modified mRNA transcript with full substitution of 5-Methoxy-U, capped (Cap 1) using CleanCap® AG and polyadenylated (120A). RNA containing lipid nanoparticles were prepared as previously described (Pardi et al., 2017). Briefly, an ethanolic solution of ALC-0315 (Patent WO2017075531), cholesterol, distearoylphosphatidylcholine (DSPC), and 2-[(polyethylene glycol)-2000] N,N ditetradecylacetamide (ALC-0159, Patent Application US14732218) was rapidly mixed with an solution of RNA in citrate buffer at pH 4.0 (composition described in Patent WO2018081480). Physical properties of the LNP such as size and polydispersity were assessed by Malvern Zetasizer and encapsulation efficiency by Ribogreen assay (Life Technologies).

1.5 Animal immunization

Thirty-three HLA-A*02:01 human transgenic mice were randomly divided into three groups and immunized twice at three-week intervals with vehicle (PBS/300 mM sucrose), 10 µg of Comirnaty® vaccine or 10 µg of MIT-T-COVID vaccine. All vaccines were administered as a 50 µL intramuscular injection. The Comirnaty® vaccine was wastage vaccine that was diluted for human administration (0.9% NaCl diluent), and remaining unusable wastage vaccine in vials was flash frozen at -80 °C. This

wastage vaccine was later thawed and immediately administered without dilution (50 µL is 10 µg of mRNA). No Comirnaty® was used that could have been administered to humans. Since Comirnaty® was thawed twice, our results may not be representative of its best performance. The MIT-T-COVID vaccine was diluted to 10 µg in 50 µL with PBS with 300 mM sucrose and then administered. In the female unchallenged cohort direct peptide immunization was performed to test the importance of mRNA-LNP delivery. Three mice were immunized with an injection of 14 short synthetic peptides in the MIT-T-COVID vaccine (15 µg per peptide, 210 µg in total) adjuvanted with 50 µg of high molecular weight polyinosine-polycytidylic acid (Poly(I:C), InvivoGen, tlrl-pic) in a volume of 150 µL via intramuscular injection. These peptides include 11 MHC class I epitopes and 3 MHC class II epitopes present in the MIT mRNA vaccine (all Supplementary Table 1 epitopes except CD4-3). CD4-3 was not included during peptide/poly IC immunization and was used as a negative control for immunogenicity measurement.

1.6 Viral challenge

At two weeks post booster immunization, eight mice of each group were challenged with 5 x 10^4 $TCID_{50}$/60 µL of SARS-CoV-2 (B.1.351/SA, Strain: hCoV-19/USA/MD-HP01542/2021) via intranasal (IN) route. Mice were weighted daily and clinically observed at least once daily and scored based on a 1–4 grading system that describes the clinical wellbeing. Three mice in each group were euthanized at 2 dpi for assessing viral loads and histopathology of the lung. The remaining five mice were continued monitored for weight changes, other signs of clinical illness, and mortality (if any) for up to 7 dpi before euthanasia for assessing antibody responses within the blood and viral loads and histopathology of the lung. Animal studies were conducted at Galveston National Laboratory at University of Texas Medical Branch at Galveston, Texas, based on a protocol approved by the Institutional Animal Care and Use Committee at UTMB at Galveston.

1.7 Assessment of mortality and morbidity

Differentially immunized and challenged mice were monitored at least once each day for the morbidity and mortality and assigned the clinical scores based on the following: 1: Healthy, 2: ruffled fur, lethargic. 3: hunched posture, orbital tightening, increased respiratory rate, and/or > 15% weight loss, and 4: dyspnea and/or cyanosis, reluctance to move when stimulated or > 20% weight loss.

1.8 Immunogenicity measurements

At 14 days post booster immunization, three mice of each group were sacrificed for harvesting splenocytes in 2 mL R10 medium (RPMI, 10% FBS, 1%P/S, 10 mM HEPES). Briefly, spleens were homogenized and subjected to filtration onto 40 µm cell strainers, followed by a wash of strainers with 10 mL PBS and centrifuged at 500 g for 5 min at 4 °C. Cell pellet was resuspended by using 2 mL of 1x red blood cell lysis buffer (eBioscience) for 2-3 min, followed by a supplement of 20 mL PBS. Resulting cell suspensions were centrifuged at 500 g for 5 min, resuspended in 2 mL R10 medium before counting the numbers under a microscope. For a brief in vitro stimulation, aliquots of 10^6 cells were incubated with indicated peptide at a final concentration of 1 µg/mL in each well of a 96-well plate. GolgiPlug (5 µg/mL, BD Bioscience) was added into the culture at 1 hr post stimulation and followed by an additional 4 hrs incubation. For cell surface staining, cultured splenocytes were resuspended in 40 µL FACS buffer containing fluorochrome-conjugated antibodies and incubated 1 hr at 4 °C followed by cell fixation using Cytofix/Cytoperm buffer (BD Bioscience) for 20 min at 4 °C. Cells were further incubated with fluorochrome-conjugated cytokine antibodies overnight. The next

day, cells were washed twice using 1 × Perm/Wash buffer and resuspended in 300 µL FACS buffer for analysis. For Foxp3 staining, cells were fixed and permeabilized by Foxp3/Transcription Factor Staining Buffer Set (Thermo Fisher Scientific) according to the instruction. Fixable Viability Dye eFluor506 (Thermo Fisher Scientific) was also used in all sample staining to exclude dead cells from our data analysis. The fluorochrome-conjugated anti-mouse antibodies included: FITC-conjugated CD3 (17A2, Biolegend), efluor450-conjugated CD4 (GK1.5, eBioscience), PE-Cy7-conjugated CD8 (53-6.7, eBioscience), PE-conjugated IFN-γ (XMG1.2, eBioscience), PerCP-eFlour710- conjugated TNF-α (MP6-XV22, eBioscience), APC-conjugated IL-2 (JES6-5H4, eBioscience). For the Foxp3 staining: Pacific Blue-conjugated CD4 (GK1.5), FITC-conjugated CD25 (PC61), Percp-Cy5.5-conjugated CTLA4 (UC-10-4B9), PE-conjugated Foxp3 (FJK-16s). For the CD44+ T cell analysis (female cohort only): FITC-conjugated CD3 (17A2, Biolegend), efluor450-conjugated CD4 (GK1.5, eBioscience), PE-Cy7-conjugated CD8 (53-6.7, eBioscience), APC-conjugated mouse/human CD44 (IM7, BioLegend). Cell acquisition was performed a BD LSR Fortessa and data were analyzed using BD FACSDiva 9.0 and FlowJo 10 (FlowJo, LLC). Lymphocytes were defined by SSC-A vs. FSC-A plots. Singlet cells were defined by FSC-H vs. FSC-A plots. Dead cells were excluded by positive staining with viability dye. $CD4^+$ and $CD8^+$ T cells were gated from $CD3^+$ cells. The cytokines secreting $CD4^+$ and $CD8^+$ T cells were then identified with IFN-γ, TNF-α, and IL-2 expression. Boundaries between positive and negative cells for the given marker were defined by the fluorescence minus one (FMO) control and adjusted according to the unstimulated splenocyte group. For the Treg cell gating strategy, $CD4^+$ T cells were gated from $CD3^+$ cells and identified by the positivity of Foxp3 and CD25 staining.

1.9 Viral titer assay

For virus quantitation, the frozen lung specimens were weighed before homogenization in PBS/2% FBS solution using the TissueLyser (Qiagen), as previously described (Tseng et al., 2007). The homogenates were centrifuged to remove cellular debris. Cell debris-free homogenates were used to quantifying infectious viruses in the standard Vero E6 cell-based infectivity assays in 96-well microtiter plates, as we routinely used in the lab (Tseng et al., 2012). Titers of virus were expressed as 50% tissue culture infectious dose per gram of tissue ($TCID_{50}$/g).

1.10 Antibody neutralization assay

Sera of mice collected at 7 dpi were used for measuring specific antibody responses. Briefly, sera were heat-inactivated (56 °C) for 30 min, were stored at -80 °C until needed. For determining the SARS-CoV-2 neutralizing antibody titers, serially two-fold (starting from 1:40) and duplicate dilutions of heat-inactivated sera were incubated with 100 TCID of SARS-CoV-2 (US_WA-1/2020 isolate) or Beta (B.1.351/SA, Strain: hCoV-19/USA/MD-HP01542/2021) at 37 °C for 1 h before transferring into designated wells of confluent Vero E6 cells grown in 96-well microtiter plates. Vero E6 cells cultured with medium with or without virus were included as positive and negative controls, respectively. After incubation at 37 °C for 3 days, individual wells were observed under the microscopy for the status of virus-induced formation of cytopathic effect. The 100% neutralizing titers (NT_{100}) of sera were expressed as the lowest dilution folds capable of completely preventing the formation of viral infection-induced cytopathic effect in 100% of the wells.

1.11 Serum IgG/IgM response by ELISA

ELISA was applied to verified serum IgG and IgM responses with SARS-CoV-2 (US_WA-1/2020 isolate) infected Vero-E6 cell lysate. In brief, SARS-CoV-2 infected Vero-E6 cell lysate was coated on 96-well plate (Corning) at 1 µg/well in PBS for overnight at 4 °C. The plates were blocked using 1% BSA/PBST for 1 h at room temperature. The 5-fold serial-dilution serum from each mouse (starting at 1:100) was then added into antigen-coated plates and incubated for 1 h at 37 °C. The plates were then washed three times with PBST (PBS/0.1% Tween-20) followed by incubation with 100 µL of anti-mouse IgG and IgM HRP conjugated secondary antibody (Jackson immunoresearch) (1:2000) for 1 h at 37 °C. After three times wash using PBST, 100 µL of ABTS substrate (Seracare) was added to the plates and incubated for 30 min in the dark. After stopped by adding 1% SDS. Then absorbance at 405 nm (OD 405 nm) was measured with the plate reader (Molecular Devices) and analyzed with GraphPad Prism Version 9.1.2.

1.12 RNA extraction and quantitative RT-PCR

Lung tissues were weighted and homogenized in 1 mL of Trizol reagent (Invitrogen) using TissueLyser (Qiagen). The RNA was then extracted using Direct-zol RNA miniprep kits (Zymo research) according to the manufacturer's instructions. 500 ng total RNA was then applied to cDNA synthesis using iScript cDNA Synthesis kit (Biorad) according to the manufacturer's instructions. The viral genomic RNA, subgenomic RNA and mice 18s rRNA has then been amplified using iQ SYBR green supermix (Biorad) and performed using CFX96 real time system (Biorad). The samples were run in duplicate using the following conditions: 95 °C for 3 min then 45 cycles of 95 °C for 15 s and 58 °C for 30 s. The level of expression was then normalized with 18s rRNA and calculated using the $2^{-\Delta\Delta Ct}$ method, as we have previously described (Tseng et al., 2007; Agrawal et al., 2015).

The primer set for SARS-CoV-2 RNA amplification is nCoV-F (ACAGGTACGTTAATAGTTAATAGCGT) and nCoV-R (ATATTGCAGCAGTACGCACACA). SgLeadSARS2-F (CGATCTCTTGTAGATCTGTTCTC) and nCoV-R (ATATTGCAGCAGTACGCACACA) were used for subgenomic SARS-CoV-2 RNA amplification. Mouse 18s rRNA was served as the internal control and amplified using 18s-F (GGACCAGAGCGAAAGCATTTGCC) and 18s-R (TCAATCTCGGGTGGCTGAACGC).

1.13 Immunohistochemistry

All slides were prepared by the Histopathology Core (UTMB) into 5 µm paraffin-embedded sections for immunohistochemistry (IHC). IHC staining and analysis were performed by UTMB according to previously published protocols (Tseng et al., 2007; Yoshikawa et al., 2009). In brief, a standard IHC sequential incubation staining protocol was followed to detect the SARS-CoV-2 spike (S) protein, $CD4^+$ cells, or $CD8^+$ cells using a rabbit-raised anti-SARS-CoV-2 S protein antibody (1:5000 dilution, ab272504, Abcam plc, Cambridge UK), a rabbit monoclonal anti-CD4 antibody (1:250 dilution, ab183685, ab183685, Abcam plc, Cambridge, UK), and a rabbit monoclonal anti-CD8 antibody (1:500 dilution, ab217344, Abcam plc, Cambridge, UK) followed by peroxidase-conjugated secondary antibody and 3,3'-Diaminobenzidine (DAB) substrate kit (MP-7802, Vector Laboratories, Burlingame, CA). Slides were counterstained with hematoxylin (MHS16-500ML, Sigma-Aldrich Inc., St. Louis, MO) and antigen expression was examined under 10X and 40X magnifications using an Olympus IX71 microscope.

$CD4^+$ and $CD8^+$ cell counts were quantified using CellProfiler 4.2.4 (Stirling et al., 2021). The image analysis pipeline included the following modules: (1) UnmixColors (for each of DAB and hematoxylin

stains), (2) ImageMath (applied to DAB stain images only; subtract 0.5 from all intensity values and set values less than 0 equal to 0), and (3) IdentifyPrimaryObjects (require object diameter 20-60 pixels [DAB stain] or 15-60 pixels [hematoxylin stain]; Global threshold strategy; Otsu thresholding method with two-class thresholding, threshold smoothing scale 1.3488, threshold correction factor 1.0, lower threshold bound 0.0, upper threshold bound 1.0, no log transform before thresholding; Shape method to distinguish clumped objects and draw dividing lines between clumped objects; automatically calculate size of smoothing filter for declumping; automatically calculate minimum allowed distance between local maxima; speed up by using lower-resolution image to find local maxima; fill holes in identified objects after declumping only; continue handling objects if excessive number of objects identified).

1.14 Statistical Analysis

One-way ANOVA tests were performed in Python using the SciPy package (Virtanen et al., 2020). Two-way ANOVA and Tukey's tests were performed in Python using the statsmodels package (Seabold and Perktold, 2010). Logrank tests were performed using GraphPad Prism.

2 Supplementary Figures and Tables

2.1 Supplementary Figures

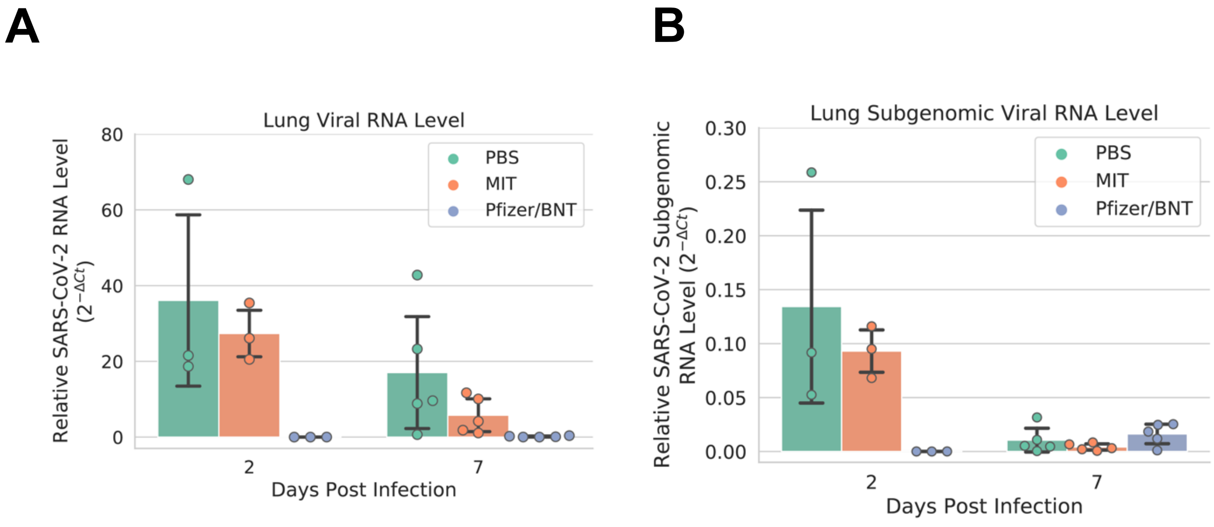

Supplementary Figure 1. (A) Lung viral RNA level, and (B) lung subgenomic viral RNA level. Error bars indicate the standard deviation around each mean.

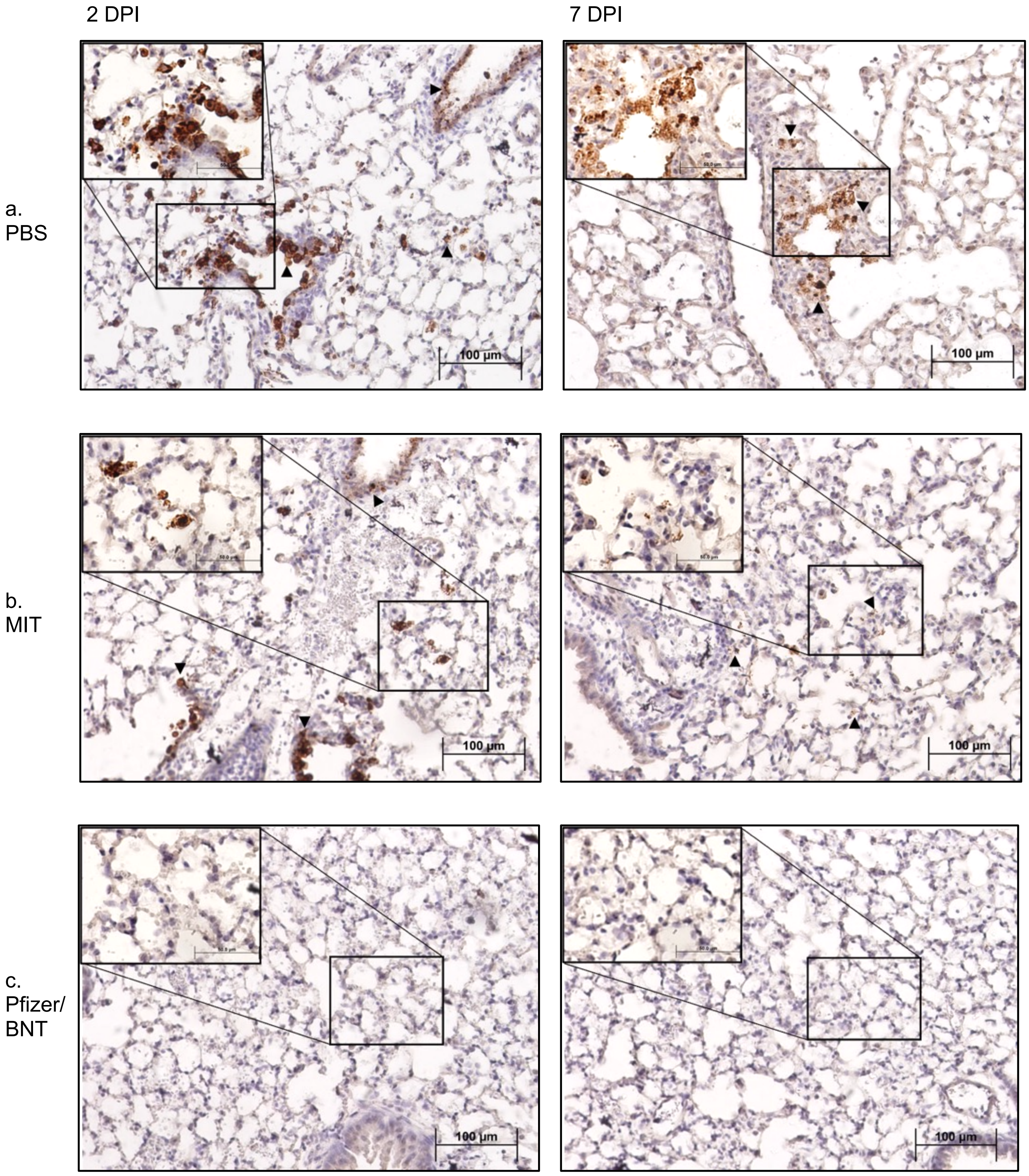
2 DPI
7 DPI
a.
PBS
b.
MIT
c.
Pfizer/
BNT
100 µm
100 µm
100 µm
100 µm
100 µm
100 µm

Supplementary Figure 2. All lung samples were subjected to IHC staining for SARS-CoV-2 spike protein (brown) with hematoxylin counterstain (blue), representative images of which are shown here. Inset images taken at 40X magnification. Black arrowheads indicate selected areas of viral infection. (a) Specimens immunized with PBS exhibited extensive staining indicative of viral infection throughout the epithelium of both the bronchioles and the alveolar sacs, with the viral infection appearing more intense at 2 days post-infection (dpi, left) than at 7 dpi (right). Although viral infection is significantly reduced by 7 dpi, viral antigen was still readily detectable throughout alveoli. (b) In comparison, specimens immunized with the MIT-T-COVID vaccine exhibited similarly extensive viral infection at 2 dpi (left) throughout the bronchiolar and alveolar epithelia, albeit somewhat reduced in intensity. However, by 7 dpi (right), viral infection was significantly reduced in both extent and intensity, with brown puncta being detected only in a few alveoli scattered throughout the tissue. (c) Contrasted with both PBS and MIT-T-COVID-immunized specimens, the Pfizer/BNT-immunized specimens exhibited significantly reduced viral infections at both 2 (left) and 7 dpi (right). With the exception of a single area at 7 dpi (see Supplementary Figure 3), viral antigen was undetected at both timepoints.

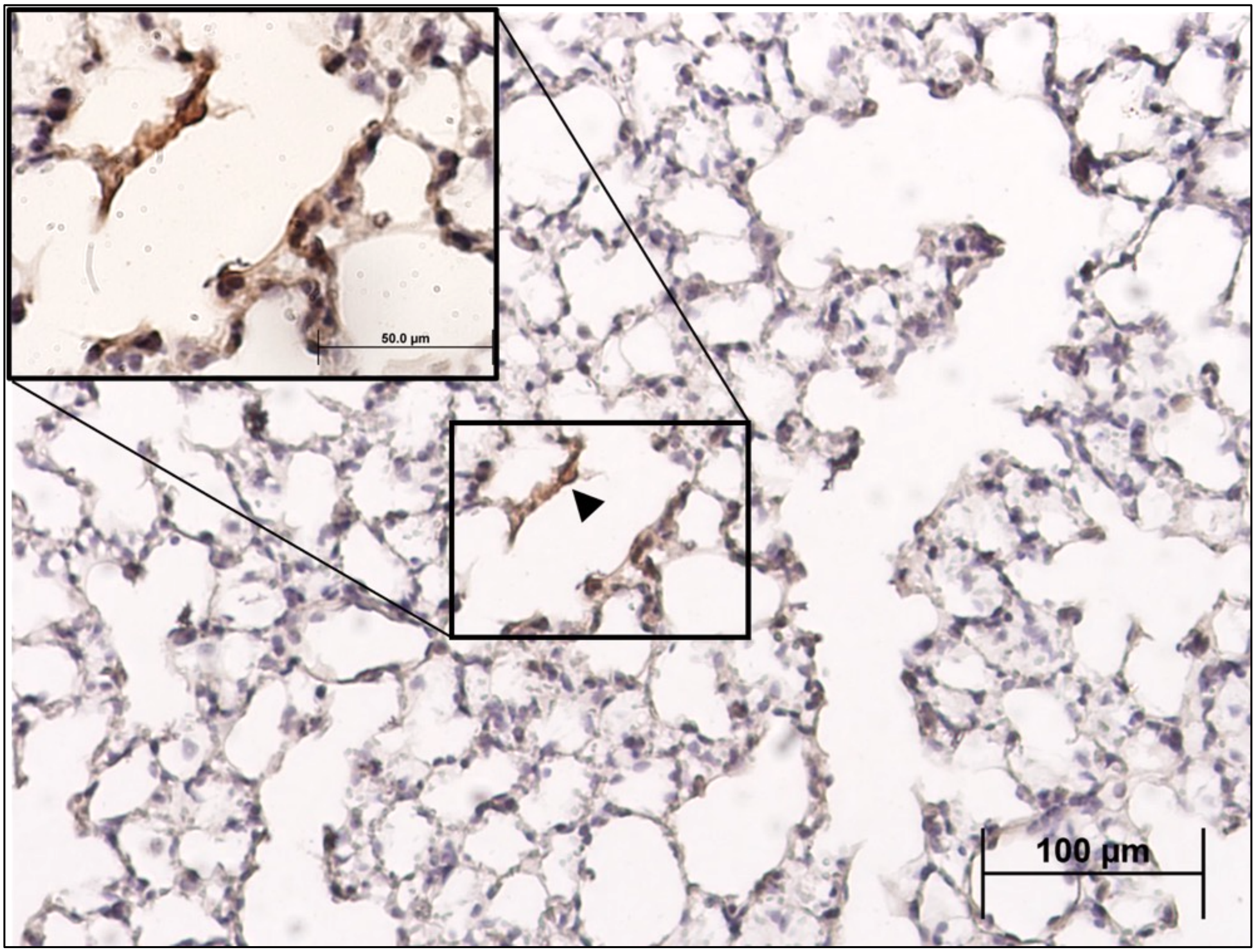

Supplementary Figure 3. Sole region of detectable SARS-CoV-2 spike antigen in alveoli of Comirnaty®-immunized lung specimens at 7 days post-infection. Black arrowhead indicates infected cell.

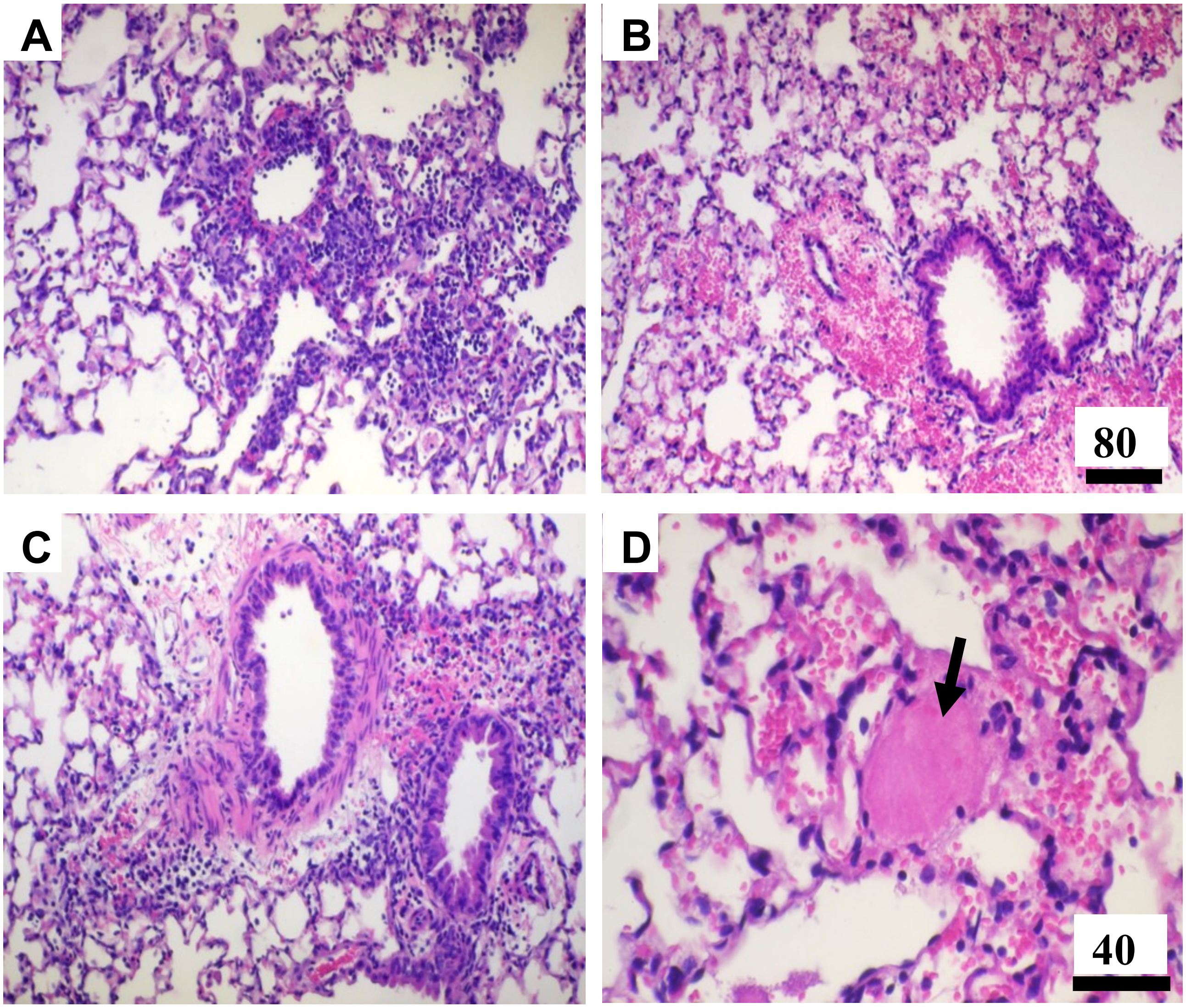

Supplementary Figure 4. Lung histopathology. Lungs of mice immunized with MIT-T-COVID (A) or Pfizer/BNT (B) are compared with those with PBS (C). At 7 dpi, the MIT-T-COVID-immunized group showed extensive lymphocytic infiltrations in perivascular regions and spaces around bronchi, bronchioles, and alveoli. There are fewer infiltrations found in the Pfizer/BNT or PBS groups and they are only localized at perivascular regions around bronchi and large bronchioles. There is widespread congestion along with hemorrhage and few foci of thromboembolism (arrow in D) seen in the Pfizer/BNT group but not others. Bar = 80 µm in A, B and C; Bar = 40 µm in D.

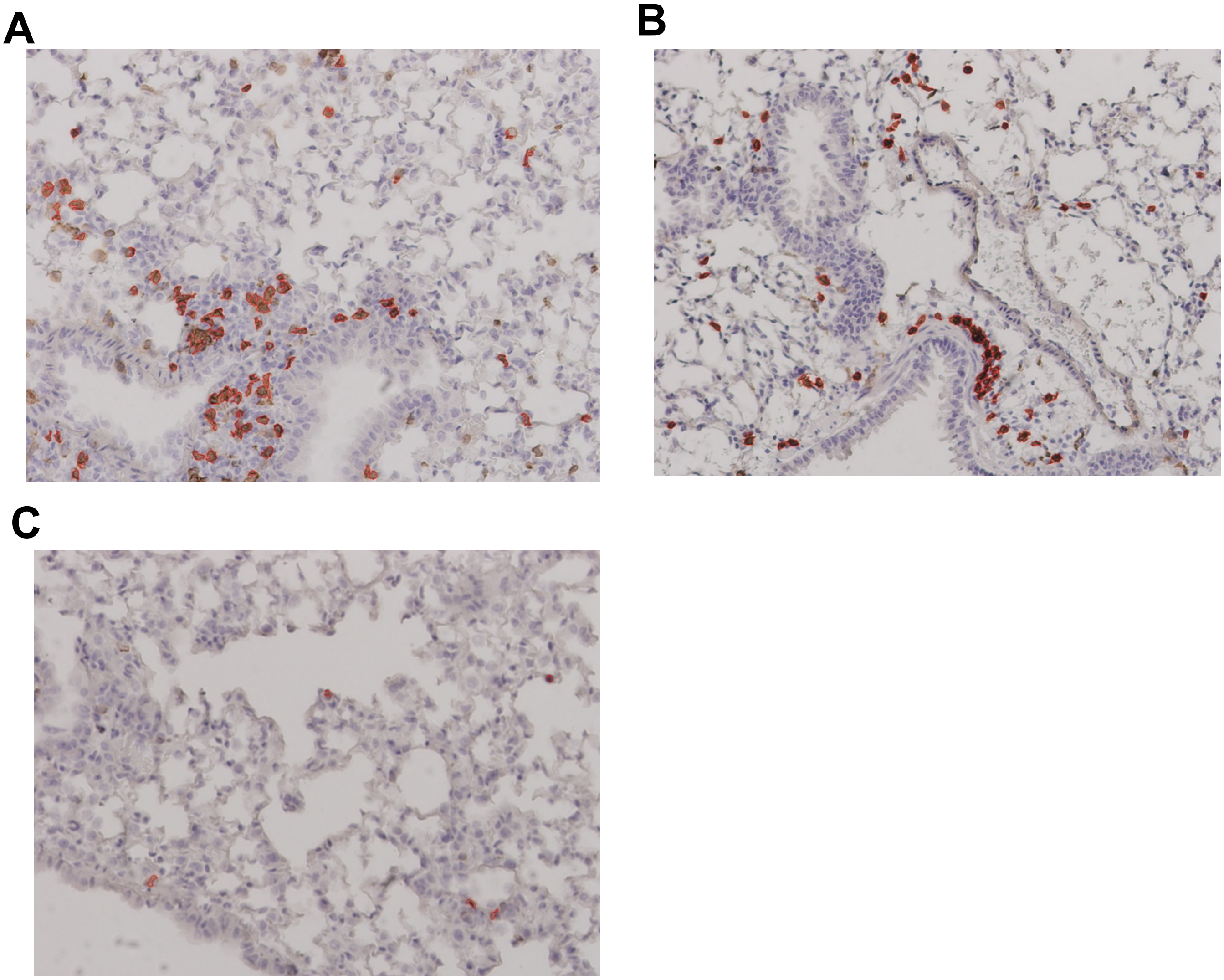

Supplementary Figure 5. Lung immunohistochemistry for $CD4^+$ cells at 7 dpi. Example $CD4^+$ stain images for (A) MIT-T-COVID, (B) Pfizer/BNT, and (C) PBS-immunized animals. Lung samples were subjected to IHC staining for CD4 (brown) with hematoxylin counterstain (blue). Images were taken at 10X magnification. Red outlines indicate $CD4^+$ cells identified and counted by CellProfiler software (Supplementary Methods). See also Figure 4 and Supplementary Figures 6-7.

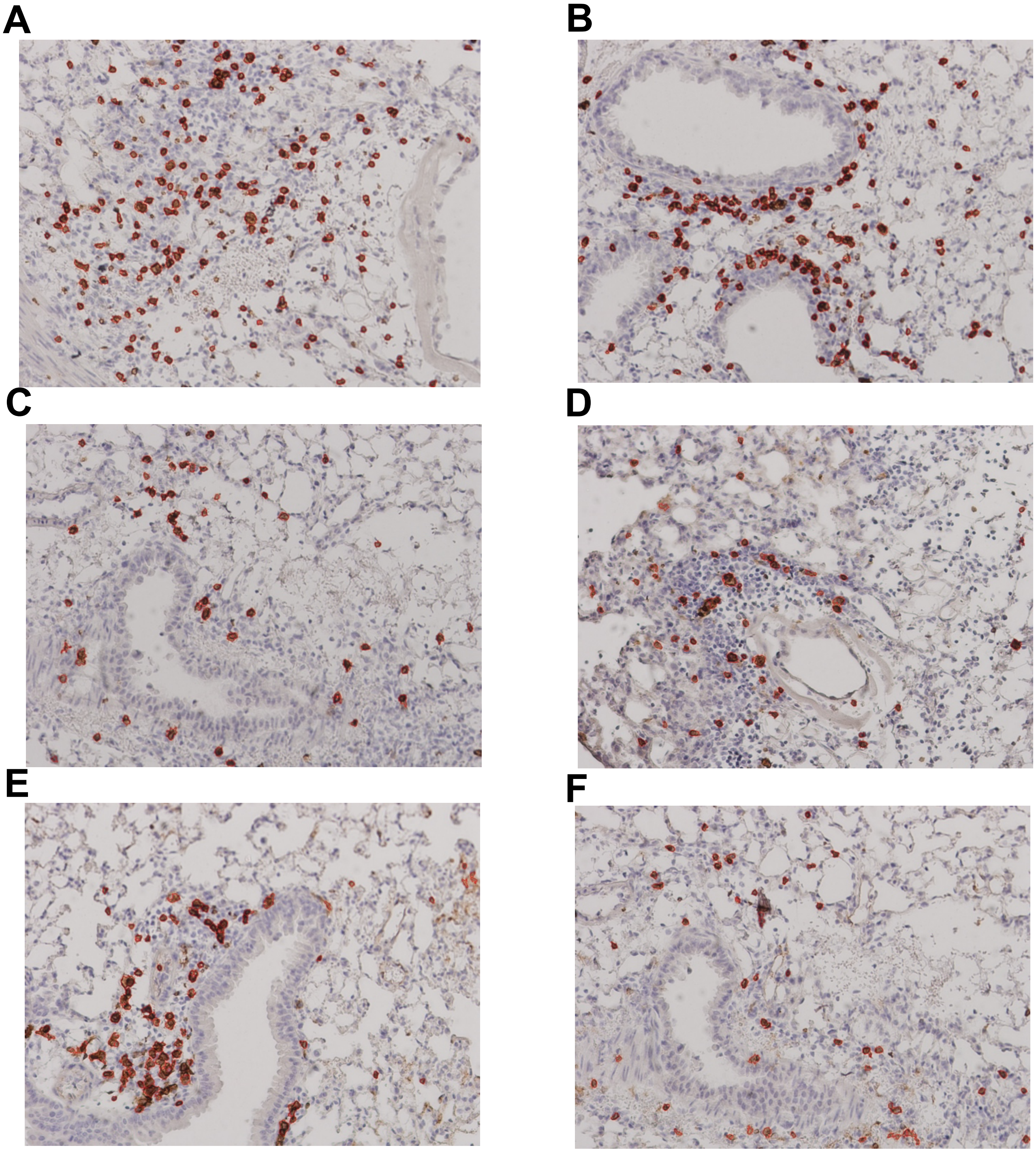

Supplementary Figure 6. Lung immunohistochemistry for $CD8^+$ and $CD4^+$ cells at 2 dpi. Example $CD8^+$ stain images for (A) MIT-T-COVID, (B) Pfizer/BNT, and (C) PBS-immunized animals. Example $CD4^+$ stain images for (D) MIT-T-COVID, (E) Pfizer/BNT, and (F) PBS-immunized animals. Lung samples were subjected to IHC staining for CD4 (brown) with hematoxylin counterstain (blue). Images were taken at 10X magnification. Red outlines indicate cells identified and counted by CellProfiler software (Supplementary Methods). See also Figure 4, Supplementary Figure 5, and Supplementary Figure 7.

Supplementary Figure 7. Complete set of cell counts in lung immunohistochemistry images for $CD8^+$ and $CD4^+$ cells at 2 and 7 dpi. Lung samples were subjected to IHC staining for CD4 or CD8 (brown) with hematoxylin counterstain (blue). Images were taken at 10X magnification. Red outlines indicate cells identified by CellProfiler software (Supplementary Methods). Mouse and image field replicates are identified as Mouse # - Field #. See also Figure 4 and Supplementary Figures 5-6.

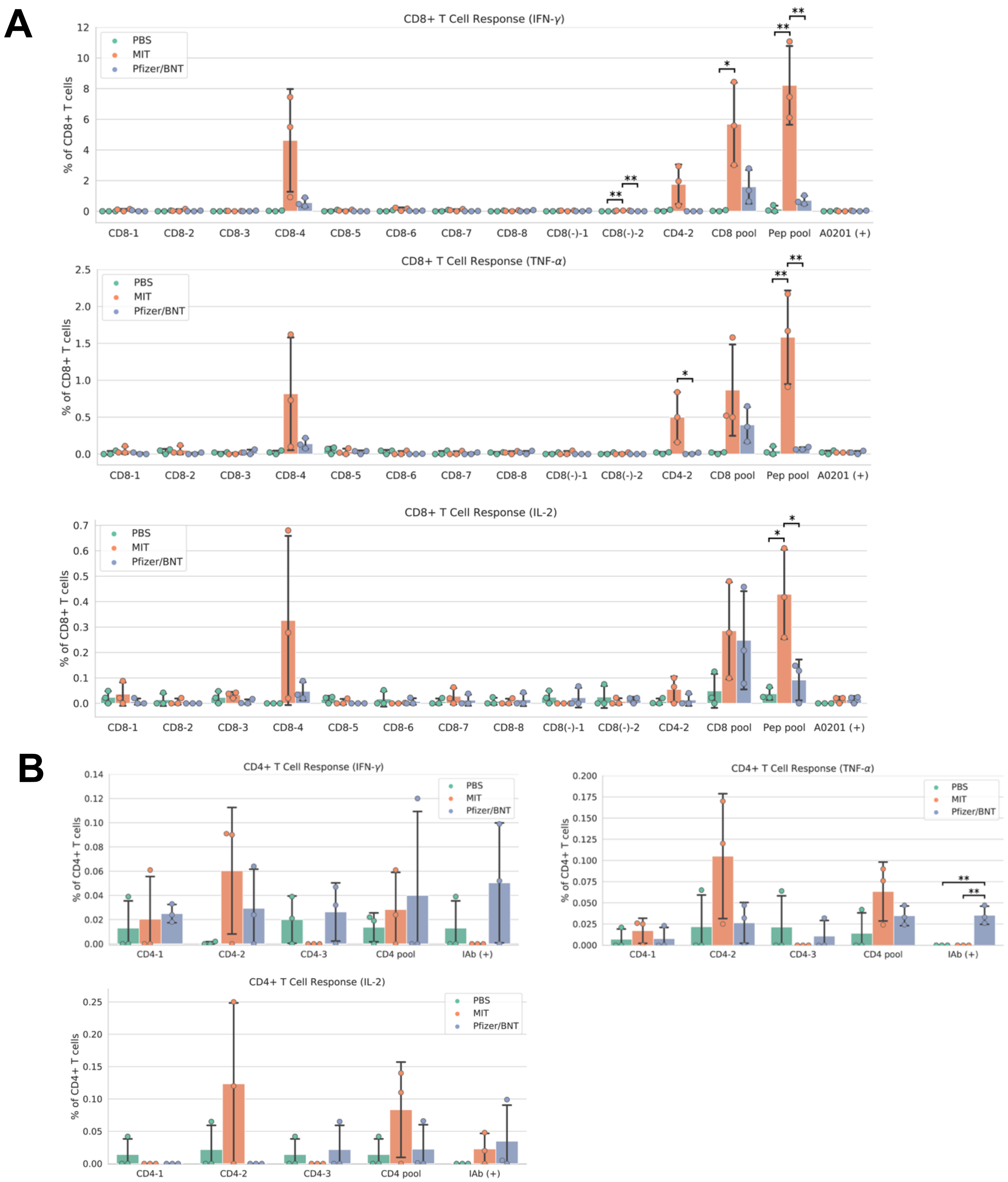

Supplementary Figure 8. Vaccine immunogenicity in female mouse cohort (Supplementary Methods). (A) $CD8^+$ T cell responses, (B) $CD4^+$ T cell responses. The CD8 pool includes MHC class I peptides CD8-1—CD8-8 (Supplementary Table 1). The CD4 pool includes MHC class II peptides CD4-1, CD4-2, and CD4-3. The Pep pool includes all query peptides in Supplementary Table 1. Error bars indicate the standard deviation around each mean. *P* values were computed by one-way ANOVA with Tukey's test. $*P < 0.05$, $**P < 0.01$. See also Supplementary Figure 9.

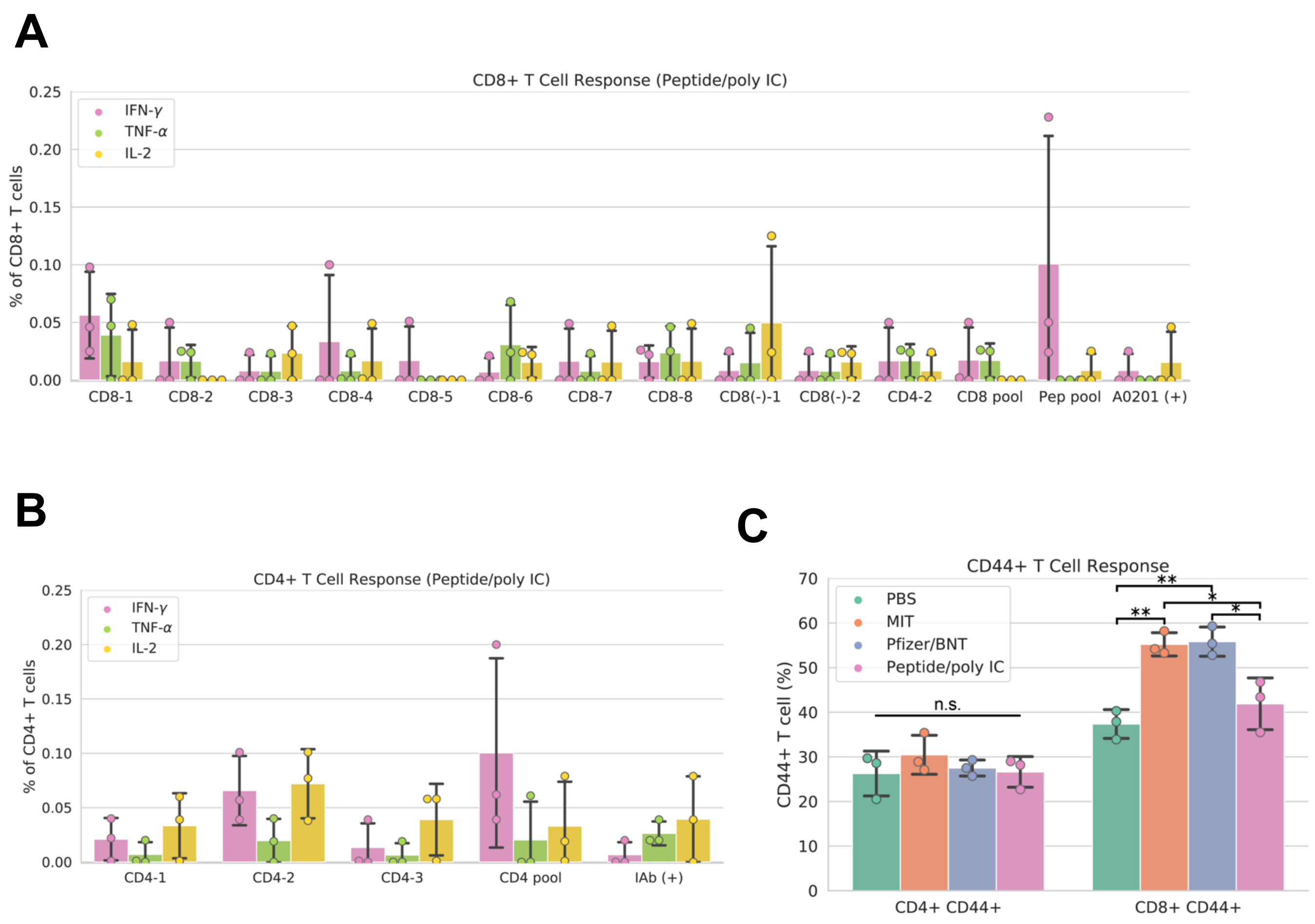

Supplementary Figure 9. Immunogenicity of Peptide/poly IC immunization in female mouse cohort (Supplementary Methods). (A) CD8$^+$ T cell responses, (B) CD4$^+$ T cell responses, and (C) CD44$^+$ T cell responses. The CD8 pool includes MHC class I peptides CD8-1—CD8-8 (Supplementary Table 1). Mice were immunized with all Supplementary Table 1 epitopes except CD4-3 (negative control). The CD4 pool includes MHC class II peptides CD4-1, CD4-2, and CD4-3. The Pep pool includes all query peptides in Supplementary Table 1. Error bars indicate the standard deviation around each mean. See also Supplementary Figure 8.

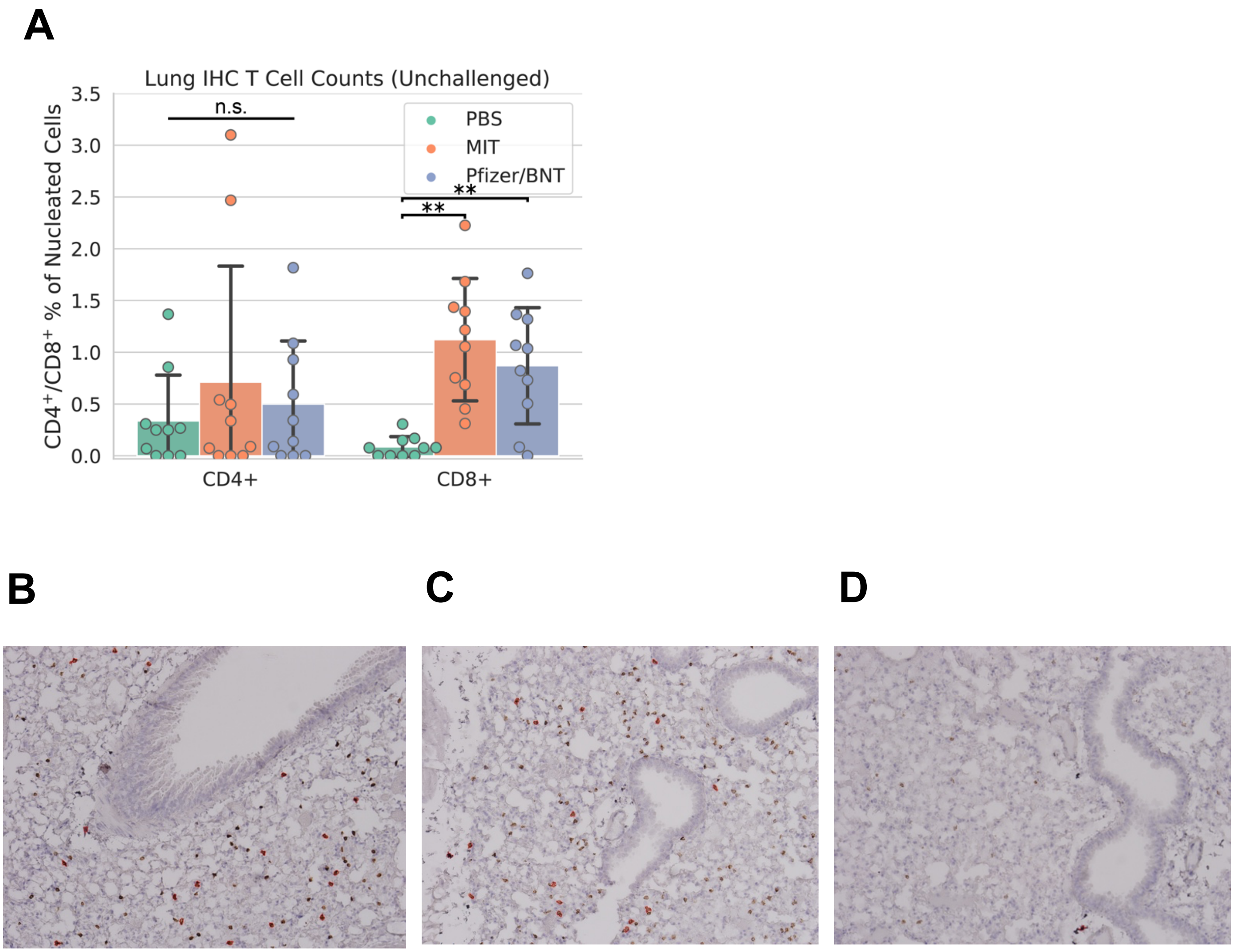

Supplementary Figure 10. Lung immunohistochemistry for CD8$^+$ and CD4$^+$ cells in unchallenged female mouse cohort. (A) Counts of CD8$^+$ and CD4$^+$ T cells expressed as a percentage of all nucleated cells visible in each field from lung tissue. Example CD8$^+$ stain images for (B) MIT-T-COVID, (C) Pfizer/BNT, and (D) PBS-immunized animals. Lung samples were subjected to IHC staining for CD8 (brown) with hematoxylin counterstain (blue). Images were taken at 10x magnification. Red outlines in (B)-(D) indicate CD8$^+$ cells identified and counted by CellProfiler software (Supplementary Methods). Error bars indicate the standard deviation around each mean. *P* values were computed by one-way ANOVA with Tukey's test. $^{**}P < 0.01$, n.s. = not significant. See also Supplementary Figure 11.

Supplementary Figure 11. Complete set of cell counts in lung immunohistochemistry images for $CD8^+$ and $CD4^+$ cells from the unchallenged female mouse cohort. Lung samples were subjected to IHC staining for CD4 or CD8 (brown) with hematoxylin counterstain (blue). Images were taken at 10X magnification. Red outlines indicate cells identified by CellProfiler software (Supplementary Methods). Mouse and image field replicates are identified as Mouse # - Field #. See also Supplementary Figure 10.

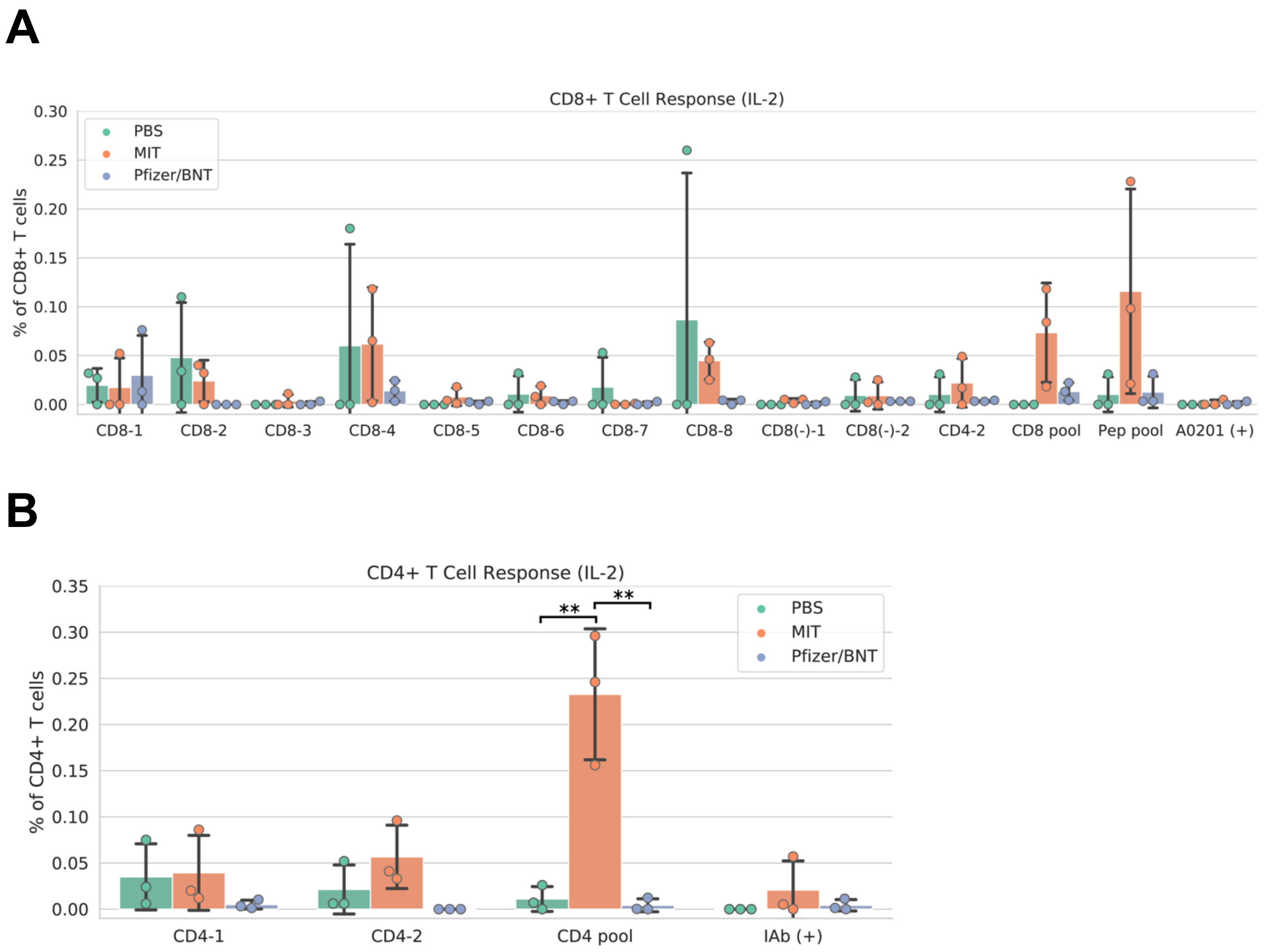

Supplementary Figure 12. Vaccine immunogenicity interleukin-2 (IL-2) measurements (male cohort). (A) $CD8^+$ T cell responses, (B) $CD4^+$ T cell responses, The CD8 pool includes MHC class I peptides CD8-1—CD8-8 (Supplementary Table 1). The CD4 pool includes MHC class II peptides CD4-1 and CD4-2. The Pep pool includes all query peptides in Supplementary Table 1 except CD4-3. Error bars indicate the standard deviation around each mean. *P* values were computed by one-way ANOVA with Tukey's test. $^{**}P < 0.01$. See also Figure 2.

2.2 Supplementary Tables

MHC Class	Vaccine Peptide	Query Peptide	Query ID	Organism	Gene	Vaccine Peptide Start-End	Query Peptide Start-End	MIRA	MCMT
1	YLYALVYFL	YLYALVYFL	CD8-1	SARS-CoV-2	ORF3a	107-115	107-115	0.86	0.19
1	RSKNPLLYDANYFLCWHTN	LLYDANYFL	CD8-2	SARS-CoV-2	ORF3a	134-152	139-147	0.70	0.44
1	FVDGVPFVV	FVDGVPFVV	CD8-3	SARS-CoV-2	ORF1ab	4726-4734	4726-4734	0.68	
1	AYYVGYLQPRTFLLKYNEN	YLQPRTFLL	CD8-4	SARS-CoV-2	S	264-282	269-277	0.66	0.62
1	FLNRFTTTL	FLNRFTTTL	CD8-5	SARS-CoV-2	ORF1ab	3482-3490	3482-3490	0.53	
1	RLTKYTMADLVYALRHFDE	TMADLVYAL	CD8-6	SARS-CoV-2	ORF1ab	4510-4528	4515-4523	0.44	
1	SIIAYTMSL	SIIAYTMSL	CD8-7	SARS-CoV-2	S	691-699	691-699	0.59	
1	LLLFVTVYSHLLLVAAGLE	TVYSHLLLV	CD8-8	SARS-CoV-2	ORF3a	84-102	89-97	0.61	
1	ATSRTLSYY	ATSRTLSYY	CD8(-)-1	SARS-CoV-2	M	171-179	171-179		
1	KTFPPTEPK	KTFPPTEPK	CD8(-)-2	SARS-CoV-2	N	361-369	361-369		
1	NLVPMVATV	NLVPMVATV	A0201 (+)	CMV	pp65	495-503	495-503		
2	KSILSPLYAFASEAARVVRSIFSRTL	PLYAFASEAARVVRSI	CD4-1	SARS-CoV-2	ORF1ab	527-552	532-547		
2	VDYGARFYFYTSKTTVASLINTLNDL	RFYFYTSKTTVASLIN	CD4-2	SARS-Cov-2	ORF1ab	1416-1441	1421-1436		
2	EEIAIILASFSASTSAFVETVKGLDY	ILASFSASTSAFVETV	CD4-3 (Female cohort only)	SARS-CoV-2	ORF1ab	471-496	476-491		
2	KPVSKMRMATPLLMQAL	KPVSKMRMATPLLMQAL	IAb (+)	Homo sapiens	CD74	102-118	102-118		

Supplementary Table 1. MIT-T-COVID vaccine peptides, query peptides (vaccine immunogenicity), peptide origin, and appearance probability in HLA-A*02:01 convalescent COVID-19 patients in Snyder et al. (2020) (MIRA column) and Kared et al. (2021) (MCMT). Selected peptides were tested by other studies for their immunogenicity in convalescent COVID-19 patients whose HLA type included HLA-A*02:01. The study by Snyder et al. (2020) included 80 HLA-A*02:01 convalescent COVID-19 patients and tested peptides individually or in small pools with the Multiplex Identification of T-cell Receptor Antigen Specificity (MIRA) assay. Query peptides were first filtered to only consider those with predicted HLA-A*02:01 binding affinity less than or equal to 25 nM. The MIRA fraction in Supplementary Table 1 is the number of individuals positive for a pool containing a query peptide divided by 80. The Kared et al. (2021) study evaluated 16 HLA-A*02:01 convalescent COVID-19 patients by mass cytometry–based multiplexed tetramer (MCMT) staining. The MCMT fraction in Supplementary Table 1 is the number of individuals positive for a query peptide divided by 16.

ATG AGG GTC ACA GCT CCT CGG ACC TTG ATC CTC CTT TTG TCT GGT GCT CTT
GCA CTG ACT GAG ACT TGG GCC GGG TCA GGA GGC AGT GGA GGA GGA GGA TCC
GGG GGT TAT TTG TAT GCT CTG GTT TAT TTT CTG GGC GGG TCC GGA GGC GGT
GGC TCT GGC GGG AGG TCC AAG AAT CCA CTT CTC TAC GAC GCA AAC TAT TTC
TTG TGT TGG CAC ACC AAT GGG GGG AGC GGT GGC GGA GGA AGC GGT GGG TTC
GTG GAC GGA GTT CCC TTT GTT GTT GGT GGG TCA GGC GGA GGA GGC TCT GGC
GGG GCT TAC TAT GTA GGG TAC CTG CAG CCC CGA ACA TTC CTT TTG AAA TAC
AAC GAG AAC GGT GGA TCC GGT GGG GGA GGA AGT GGA GGG TTT CTG AAT AGA
TTC ACC ACC ACT CTG GGA GGT TCT GGC GGC GGG GGT TCT GGT GGA CGG CTG
ACT AAA TAC ACA ATG GCC GAT CTT GTT TAC GCA TTG CGG CAT TTT GAT GAG
GGA GGC AGT GGC GGA GGG GGA TCC GGC GGC AGC ATA ATA GCT TAC ACC ATG
TCA CTG GGA GGG AGC GGA GGG GGC GGG AGC GGC GGT TTG CTC CTT TTC GTG
ACA GTG TAT AGC CAT CTC CTT CTG GTG GCA GCT GGC CTT GAA GGG GGG AGC
GGT GGA GGA GGT AGC GGT GGC GCC ACT TCT AGG ACA TTG AGT TAC TAT GGG
GGC AGC GGA GGA GGA GGT TCT GGA GGC AAG ACC TTC CCC CCT ACA GAG CCC
AAG GGA GGT TCC GGC GGC GGG GGC AGT GGT GGG GAA GAG ATC GCC ATT ATC
TTG GCT TCC TTT AGT GCT TCA ACA AGC GCT TTT GTA GAG ACC GTA AAG GGC
CTC GAT TAT GGA GGT TCA GGG GGA GGA GGC TCA GGT GGG AAG TCA ATA CTG
TCT CCT CTT TAT GCA TTT GCA TCA GAG GCT GCA AGA GTT GTC CGA TCC ATT TTT
TCT CGC ACT CTT GGG GGG TCC GGC GGA GGC GGG TCT GGC GGT GTG GAT TAT
GGT GCT AGG TTT TAT TTT TAC ACT TCC AAA ACC ACT GTT GCC TCT CTC ATA AAT
ACC CTC AAT GAC CTG GGA GGG TCT GGT GGC GGG GGG AGT GGC GGG AAT CTT
GTT CCT ATG GTT GCA ACA GTA GGA GGT TCC GGA GGG GGT GGC AGC GGA GGA
AAG CCA GTG TCC AAG ATG AGA ATG GCA ACC CCT TTG CTG ATG CAG GCC CTG
GGT GGC AGT CTG GGA GGT GGT GGC TCC GGC ATC GTA GGT ATA GTC GCC GGA
CTT GCA GTT TTG GCC GTG GTA GTG ATA GGC GCA GTT GTT GCC ACC GTT ATG
TGC CGA CGA AAG AGT TCA GGC GGC AAG GGT GGT TCT TAC TCC CAA GCT GCA
AGC TCC GAC TCC GCT CAG GGG AGT GAT GTT AGC TTG ACT GCA TGA

Supplementary Table 2. Nucleic acid sequence for assembled vaccine construct of Figure 1A.

Additional References for Supplementary Material

1. Snyder TM, Gittelman RM, Klinger M, May DH, Osborne EJ, Taniguchi R, Zahid HJ, Kaplan IM, Dines JN, Noakes MT, Pandya R, Chen X, Elasady S, Svejnoha E, Ebert P, Pesesky MW, De Almeida P, O'Donnell H, DeGottardi Q, Keitany G, Lu J, Vong A, Elyanow R, Fields P, Greissl J, Baldo L, Semprini S, Cerchione C, Nicolini F, Mazza M, Delmonte OM, Dobbs K, Laguna-Goya R, Carreño-Tarragona G, Barrio S, Imberti L, Sottini A, Quiros-Roldan E, Rossi C, Biondi A, Bettini LR, D'Angio M, Bonfanti P, Tompkins MF, Alba C, Dalgard C, Sambri V, Martinelli G, Goldman JD, Heath JR, Su HC, Notarangelo LD, Paz-Artal E, Martinez-Lopez J, Carlson JM, Robins HS. Magnitude and Dynamics of the T-Cell Response to SARS-CoV-2 Infection at Both Individual and Population Levels. medRxiv [Preprint]. 2020 Sep 17:2020.07.31.20165647. doi: 10.1101/2020.07.31.20165647. PMID: 32793919; PMCID: PMC7418734.

2. Reynisson B, Alvarez B, Paul S, Peters B, Nielsen M. NetMHCpan-4.1 and NetMHCIIpan-4.0: improved predictions of MHC antigen presentation by concurrent motif deconvolution and integration of MS MHC eluted ligand data. Nucleic Acids Res. 2020 Jul 2;48(W1):W449-W454. doi: 10.1093/nar/gkaa379. PMID: 32406916; PMCID: PMC7319546.

3. O'Donnell TJ, Rubinsteyn A, Laserson U. MHCflurry 2.0: Improved Pan-Allele Prediction of MHC Class I-Presented Peptides by Incorporating Antigen Processing. Cell Syst. 2020 Jul 22;11(1):42-48.e7. doi: 10.1016/j.cels.2020.06.010. Epub 2020 Jul 14. Erratum in: Cell Syst. 2020 Oct 21;11(4):418-419. PMID: 32711842.

4. Zeng H, Gifford DK. Quantification of Uncertainty in Peptide-MHC Binding Prediction Improves High-Affinity Peptide Selection for Therapeutic Design. Cell Syst. 2019 Aug 28;9(2):159-166.e3. doi: 10.1016/j.cels.2019.05.004. Epub 2019 Jun 5. PMID: 31176619; PMCID: PMC6715517.

5. UniProt Consortium. UniProt: a worldwide hub of protein knowledge. Nucleic Acids Res. 2019 Jan 8;47(D1):D506-D515. doi: 10.1093/nar/gky1049. PMID: 30395287; PMCID: PMC6323992.

6. Pardi N, Hogan MJ, Pelc RS, Muramatsu H, Andersen H, DeMaso CR, Dowd KA, Sutherland LL, Scearce RM, Parks R, Wagner W, Granados A, Greenhouse J, Walker M, Willis E, Yu JS, McGee CE, Sempowski GD, Mui BL, Tam YK, Huang YJ, Vanlandingham D, Holmes VM, Balachandran H, Sahu S, Lifton M, Higgs S, Hensley SE, Madden TD, Hope MJ, Karikó K, Santra S, Graham BS, Lewis MG, Pierson TC, Haynes BF, Weissman D. Zika virus protection by a single low-dose nucleoside-modified mRNA vaccination. Nature. 2017 Mar 9;543(7644):248-251. doi: 10.1038/nature21428. Epub 2017 Feb 2. PMID: 28151488; PMCID: PMC5344708.

7. Tseng CTK, Huang C, Newman P, Wang N, Narayanan K, Watts DM, Makino S, Packard M, Zaki SR, Chan TS, and Peters CJ. 2007. SARS Coronavirus Infection of Transgenic Mice bearing the Human Angiotensin Converting Enzyme 2 (hACE2) Virus Receptor. *JVI* 81: 1162-1173.

8. Tseng CT, Sbrana E, Iwata-Yoshikawa N, Newman PC, Garron T, Atmar RL, Peters CJ, Couch RB. Immunization with SARS coronavirus vaccines leads to pulmonary immunopathology on challenge with the SARS virus. PLoS One. 2012;7(4):e35421. doi: 10.1371/journal.pone.0035421. Epub 2012 Apr 20. Erratum in: PLoS One. 2012;7(8). doi:10.1371/annotation/2965cfae-b77d-4014-8b7b-236e01a35492. PMID: 22536382; PMCID: PMC3335060.

9. Agrawal AS, Garron T, Tao X, Peng BH, Wakamiya M, Chan TS, Couch RB, Tseng CT. Generation of a transgenic mouse model of Middle East respiratory syndrome coronavirus infection and disease. J Virol. 2015 Apr;89(7):3659-70. doi: 10.1128/JVI.03427-14. Epub 2015 Jan 14. PMID: 25589660; PMCID: PMC4403411.

10. Yoshikawa N, Yoshikawa T, Hill T, Huang C, Watts DM, Makino S, Milligan G, Chan T, Peters CJ, Tseng CT. Differential virological and immunological outcome of severe acute respiratory syndrome coronavirus infection in susceptible and resistant transgenic mice expressing human angiotensin-converting enzyme 2. J Virol. 2009 Jun;83(11):5451-65. doi: 10.1128/JVI.02272-08. Epub 2009 Mar 18. PMID: 19297479; PMCID: PMC2681954.

11. Stirling DR, Swain-Bowden MJ, Lucas AM, Carpenter AE, Cimini BA, Goodman A. CellProfiler 4: improvements in speed, utility and usability. BMC Bioinformatics. 2021 Sep 10;22(1):433. doi: 10.1186/s12859-021-04344-9. PMID: 34507520; PMCID: PMC8431850.

12. Virtanen P, Gommers R, Oliphant TE, Haberland M, Reddy T, Cournapeau D, Burovski E, Peterson P, Weckesser W, Bright J, van der Walt SJ, Brett M, Wilson J, Millman KJ, Mayorov N, Nelson ARJ, Jones E, Kern R, Larson E, Carey CJ, Polat İ, Feng Y, Moore EW, VanderPlas J, Laxalde D, Perktold J, Cimrman R, Henriksen I, Quintero EA, Harris CR, Archibald AM, Ribeiro AH, Pedregosa F, van Mulbregt P; SciPy 1.0 Contributors. SciPy 1.0: fundamental algorithms for scientific computing in Python. Nat Methods. 2020 Mar;17(3):261-272. doi: 10.1038/s41592-019-0686-2. Epub 2020 Feb 3. Erratum in: Nat Methods. 2020 Feb 24;: PMID: 32015543; PMCID: PMC7056644.

13. Seabold, S., Perktold, J. Statsmodels: Econometric and statistical modeling with python. Proceedings of the 9th Python in Science Conference. 2010; 57(61).

14. Kared H, Redd AD, Bloch EM, Bonny TS, Sumatoh H, Kairi F, Carbajo D, Abel B, Newell EW, Bettinotti MP, Benner SE, Patel EU, Littlefield K, Laeyendecker O, Shoham S, Sullivan D, Casadevall A, Pekosz A, Nardin A, Fehlings M, Tobian AA, Quinn TC. SARS-CoV-2-specific CD8+ T cell responses in convalescent COVID-19 individuals. J Clin Invest. 2021 Mar 1;131(5):e145476. doi: 10.1172/JCI145476. PMID: 33427749; PMCID: PMC7919723.
