# Supplementary material for: A pan-variant mRNA-LNP T cell vaccine protects HLA transgenic mice from mortality after infection with SARS-CoV-2 Beta
Source: Front Immunol. 2023 Mar 9;14:1135815. doi: 10.3389/fimmu.2023.1135815 (PMC10033589; doi:10.3389/fimmu.2023.1135815)

Supplementary Figure 7
Page 1/29

MIT-T-COVID Lung 1-1
2 dpi

CD8$^+$/CD4$^+$ Cell Annotations

Nucleated Cell Annotations

CD8$^+$

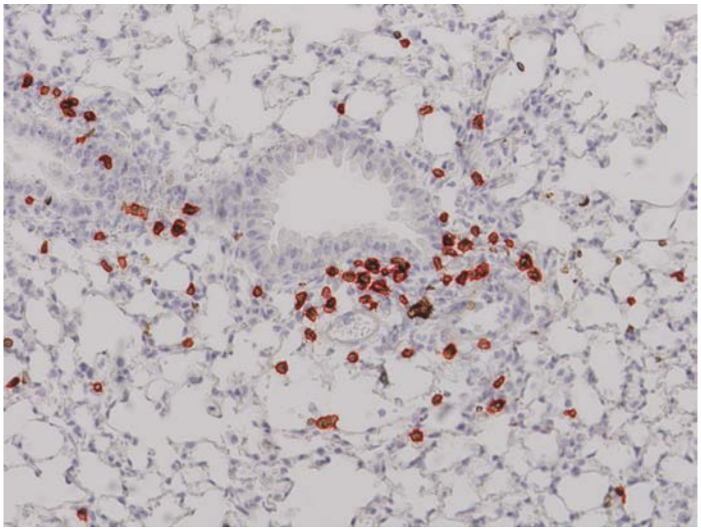

75 CD8$^+$ cells

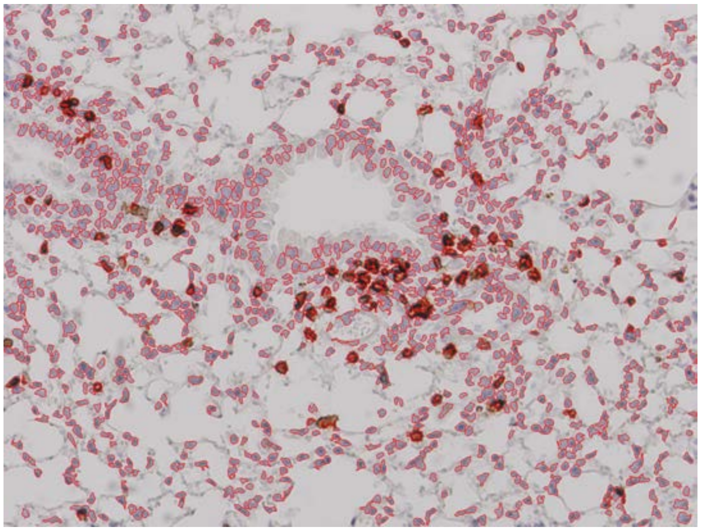

1170 nucleated cells

CD4$^+$

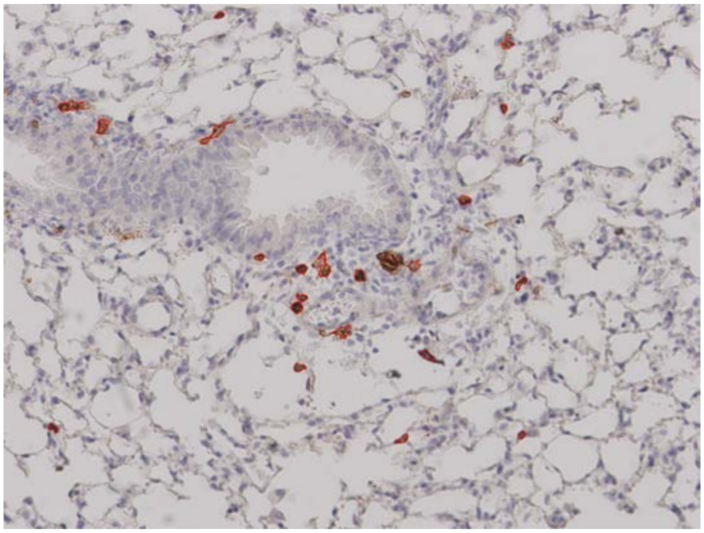

26 CD4$^+$ cells

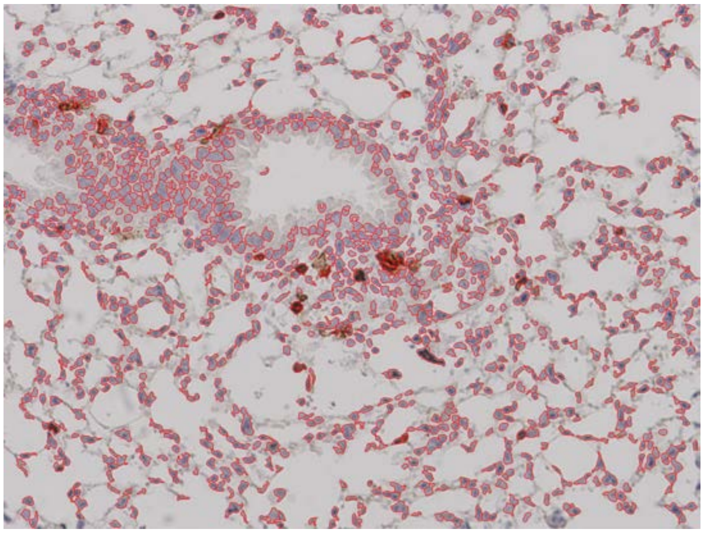

1194 nucleated cells

Supplementary Figure 7

MIT-T-COVID Lung 1-2
2 dpi

CD8$^+$/CD4$^+$ Cell Annotations | Nucleated Cell Annotations

CD8$^+$

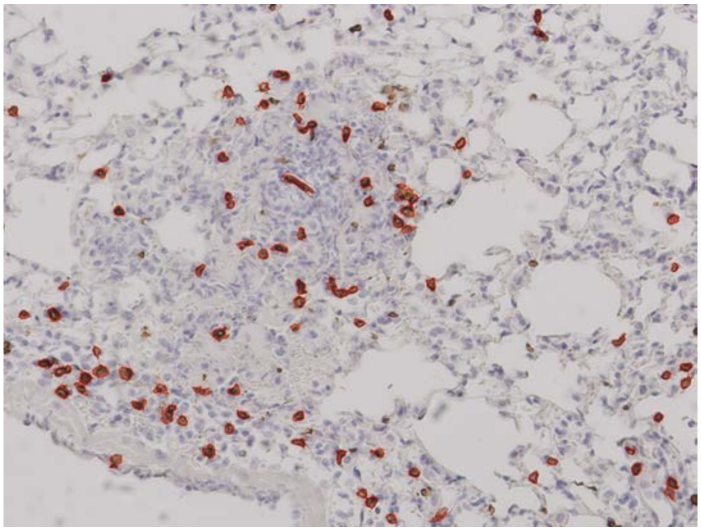

89 CD8$^+$ cells

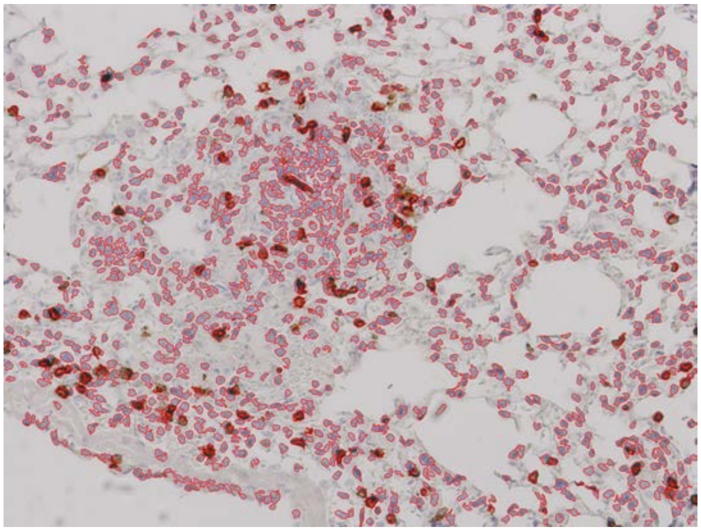

1220 nucleated cells

CD4$^+$

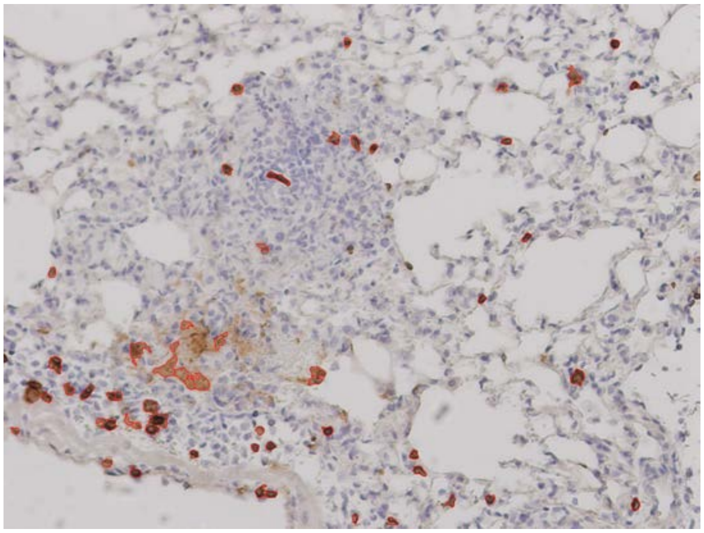

56 CD4$^+$ cells

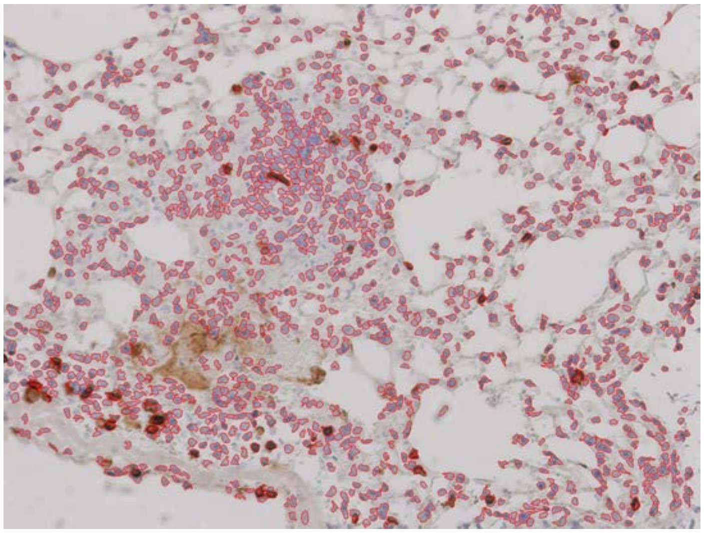

1248 nucleated cells

Supplementary Figure 7
Page 3/29

MIT-T-COVID Lung 1-3
2 dpi

CD8$^+$/CD4$^+$ Cell Annotations | Nucleated Cell Annotations

CD8$^+$

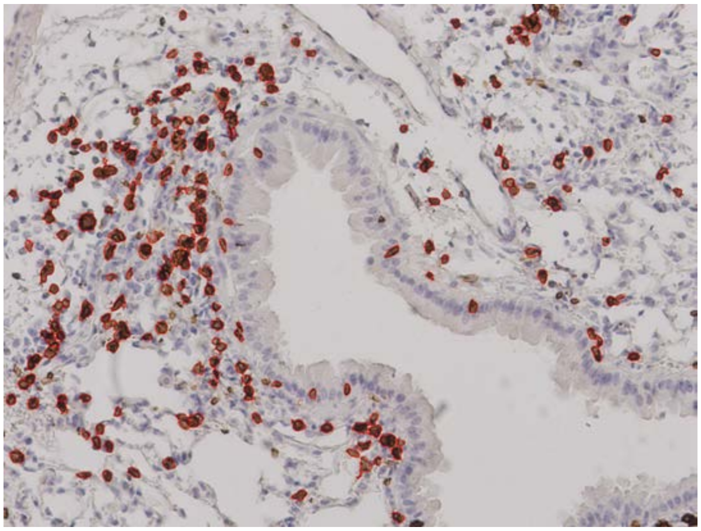

188 CD8$^+$ cells

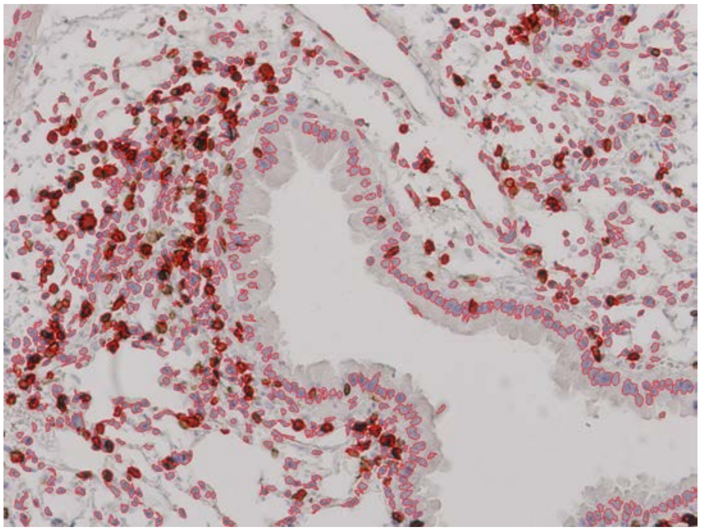

1156 nucleated cells

CD4$^+$

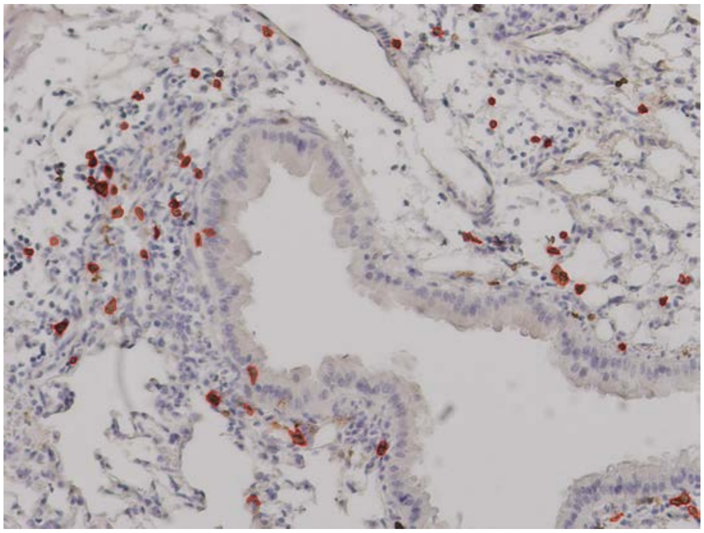

51 CD4$^+$ cells

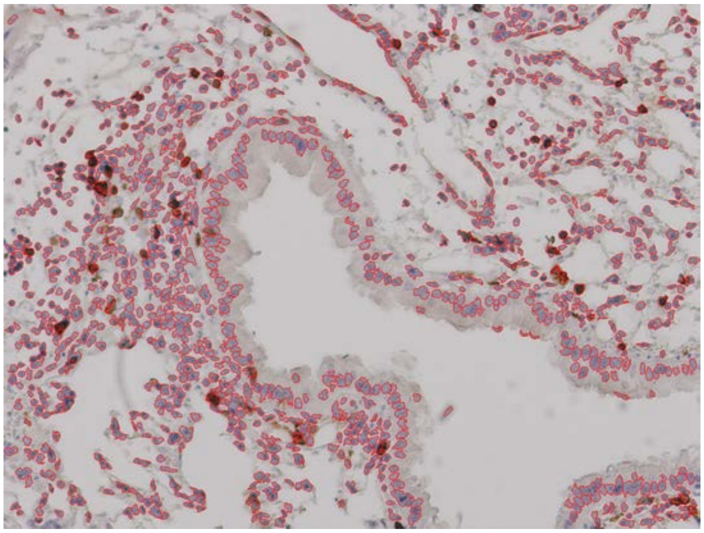

1121 nucleated cells

Supplementary Figure 7
Page 4/29

MIT-T-COVID Lung 2-1
2 dpi

CD8⁺/CD4⁺ Cell Annotations | Nucleated Cell Annotations

CD8⁺

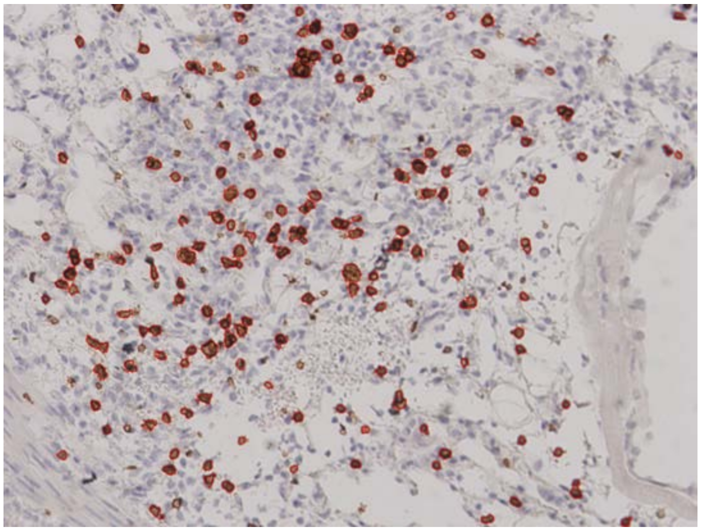

158 $CD8^+$ cells

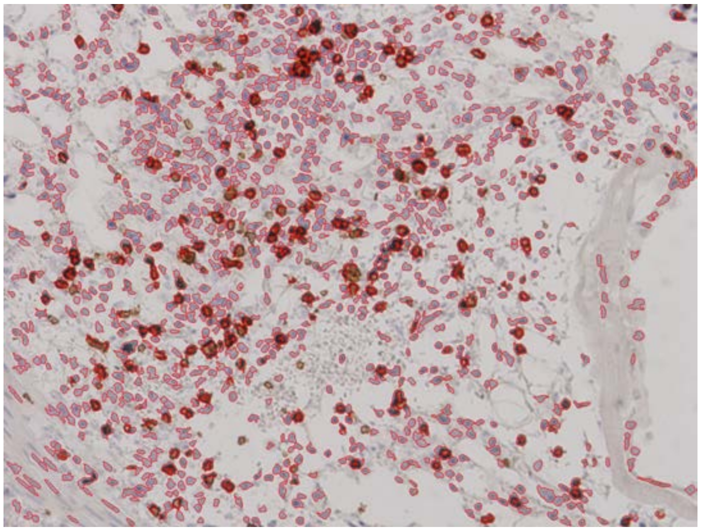

1268 nucleated cells

CD4⁺

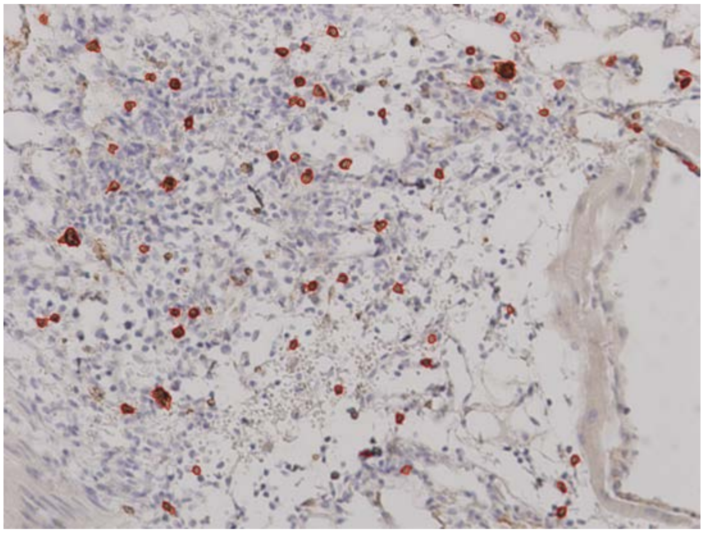

61 $CD4^+$ cells

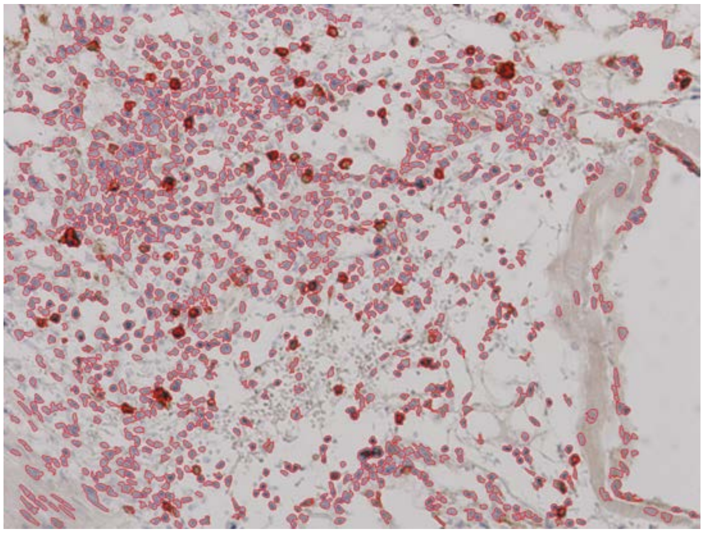

1309 nucleated cells

MIT-T-COVID Lung 3-1
2 dpi

CD8⁺/CD4⁺ Cell Annotations | Nucleated Cell Annotations

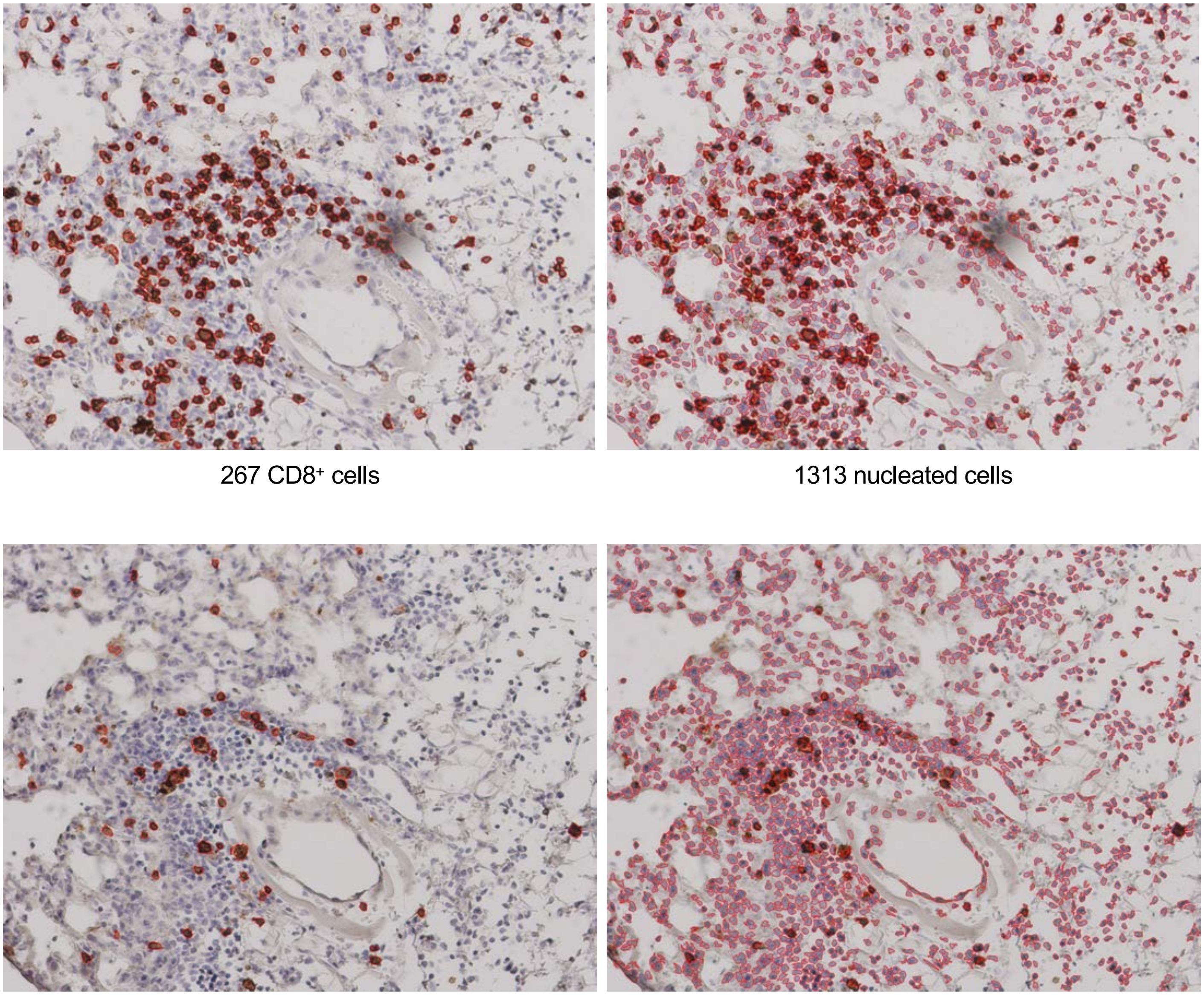

CD8$^+$: 267 CD8$^+$ cells | 1313 nucleated cells

CD4$^+$: 56 CD4$^+$ cells | 1455 nucleated cells

Supplementary Figure 7
Page 6/29

Pfizer/BNT Lung 1-1
2 dpi

CD8$^+$/CD4$^+$ Cell Annotations | Nucleated Cell Annotations

CD8$^+$

CD4$^+$

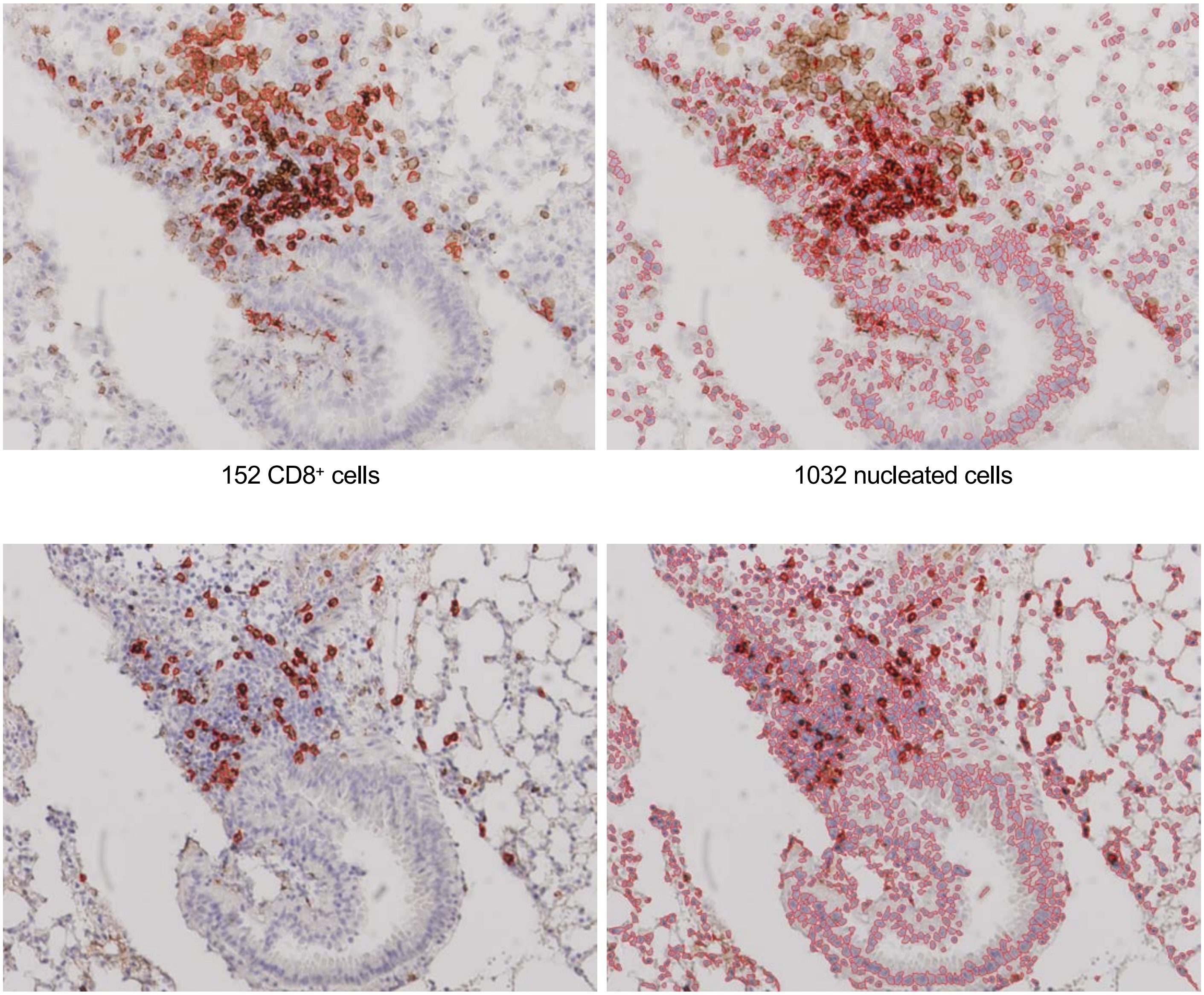

152 CD8$^+$ cells

1032 nucleated cells

72 CD4$^+$ cells

1386 nucleated cells

Pfizer/BNT Lung 2-1
2 dpi

CD8$^+$/CD4$^+$ Cell Annotations | Nucleated Cell Annotations

CD8$^+$

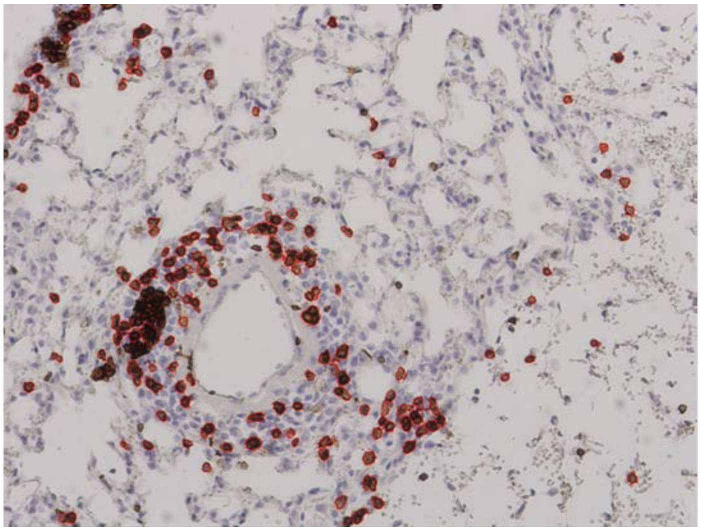

138 CD8$^+$ cells

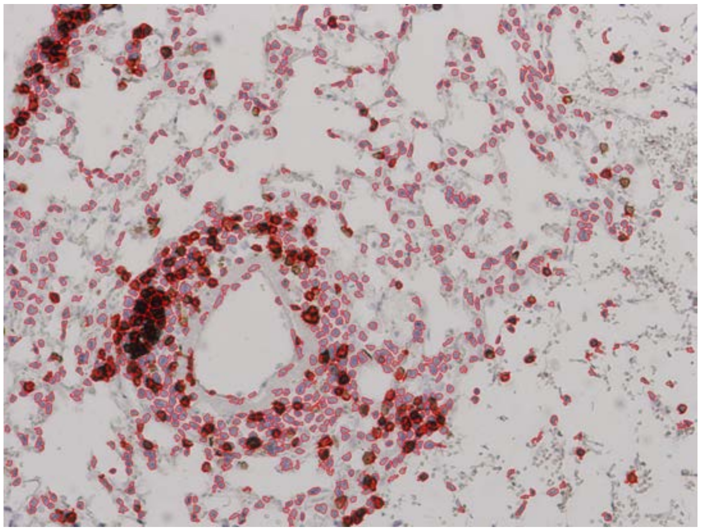

989 nucleated cells

CD4$^+$

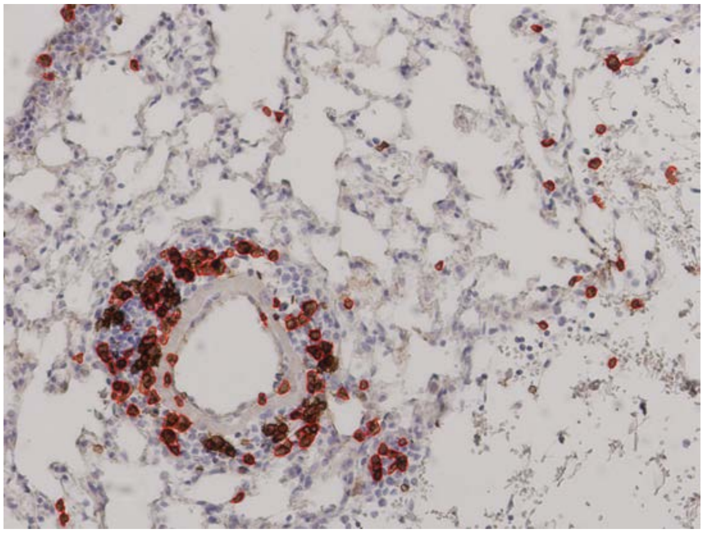

90 CD4$^+$ cells

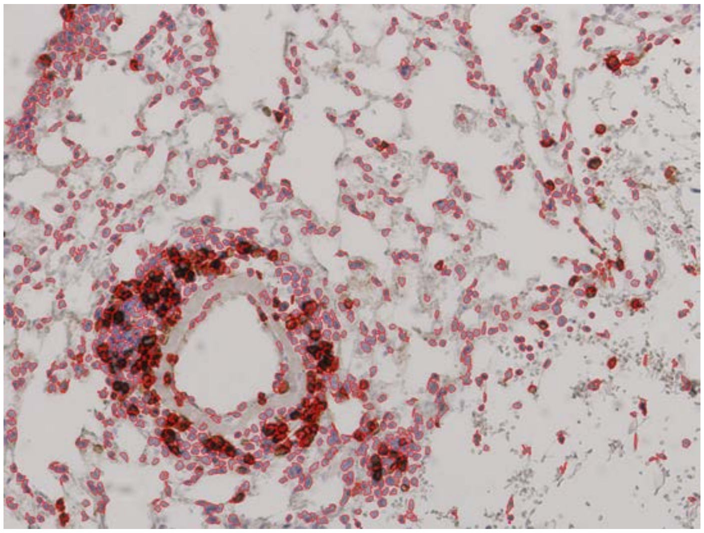

1009 nucleated cells

Supplementary Figure 7

Pfizer/BNT Lung 2-2
2 dpi

CD8$^+$/CD4$^+$ Cell Annotations | Nucleated Cell Annotations

CD8$^+$

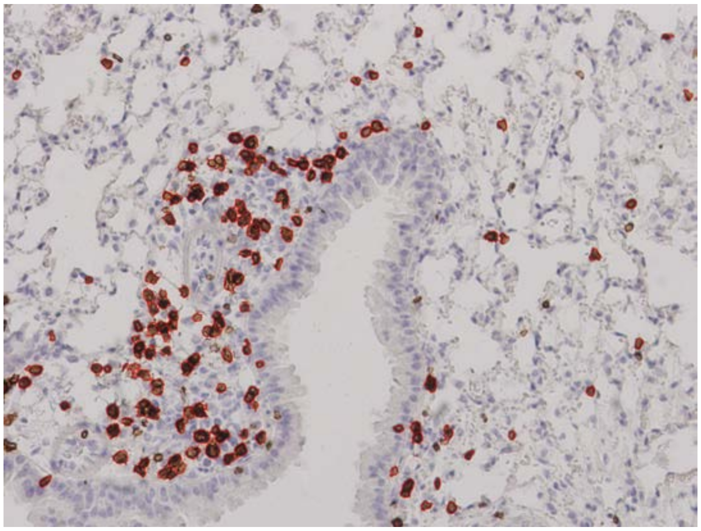

123 CD8$^+$ cells

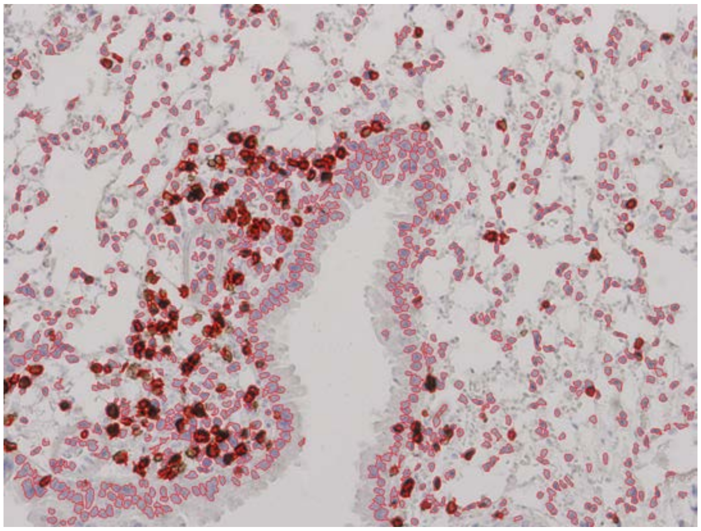

1072 nucleated cells

CD4$^+$

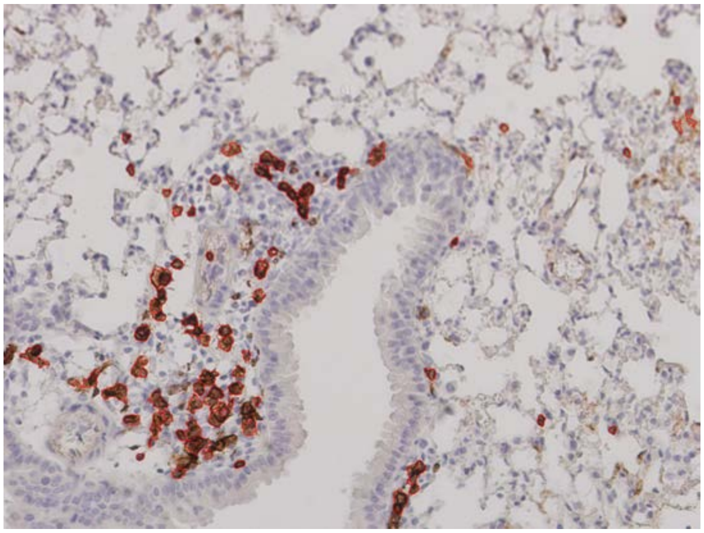

82 CD4$^+$ cells

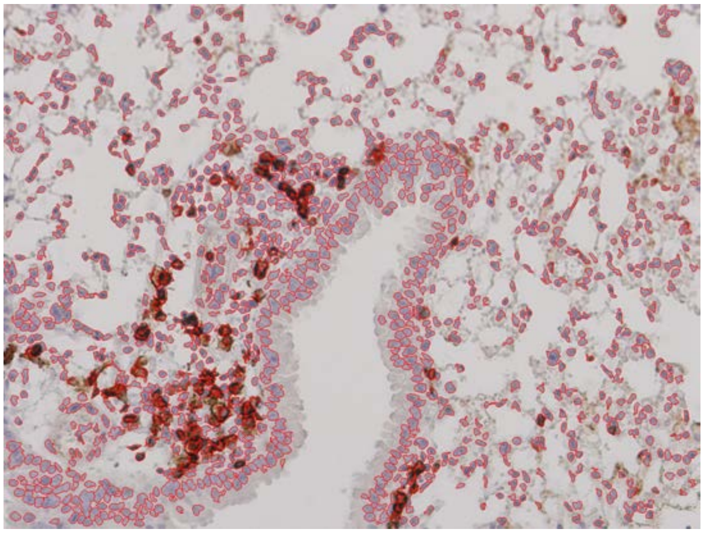

1178 nucleated cells

Supplementary Figure 7

Pfizer/BNT Lung 3-1
2 dpi

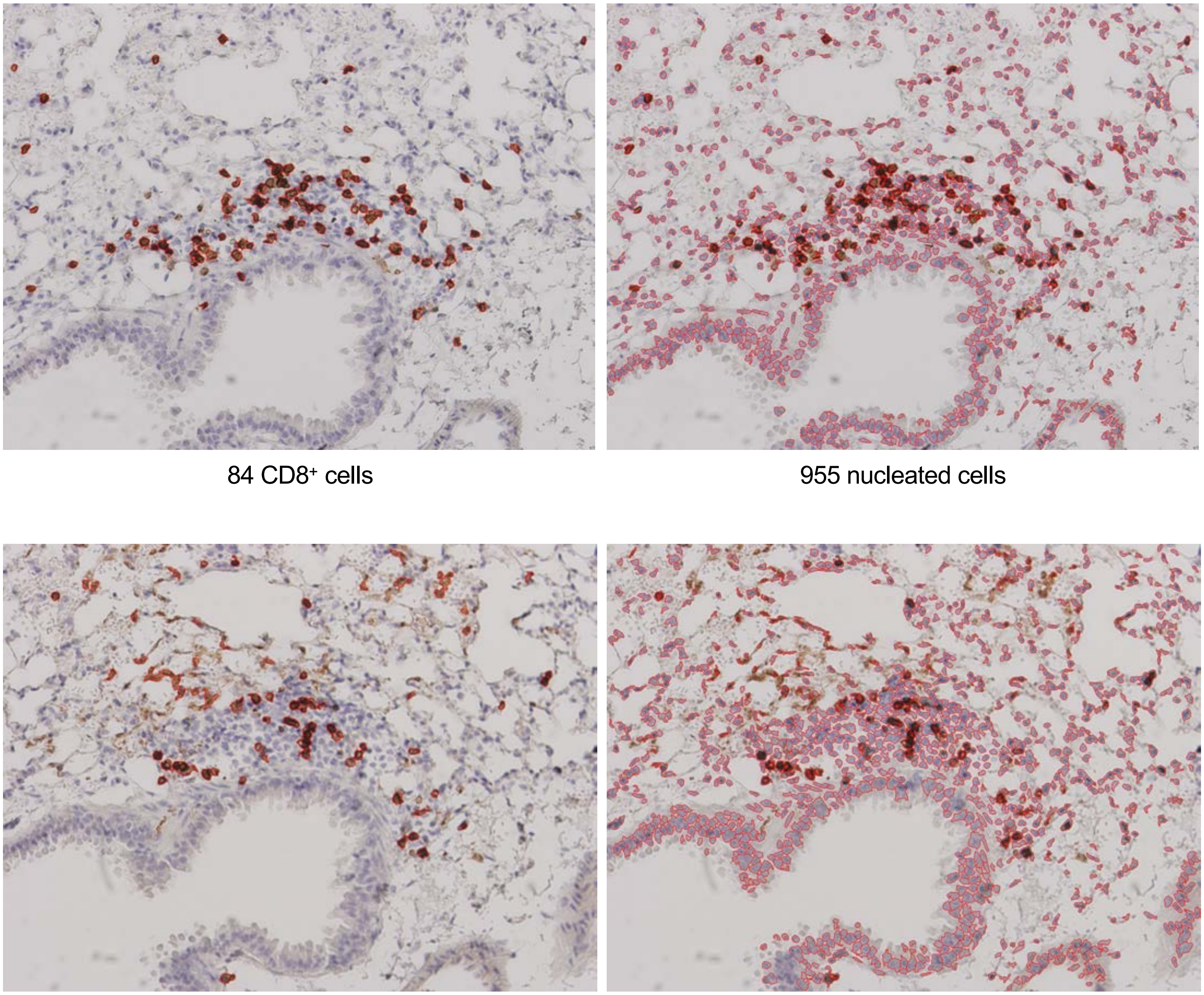

Supplementary Figure 7

Pfizer/BNT Lung 3-2
2 dpi

CD8$^+$/CD4$^+$ Cell Annotations | Nucleated Cell Annotations

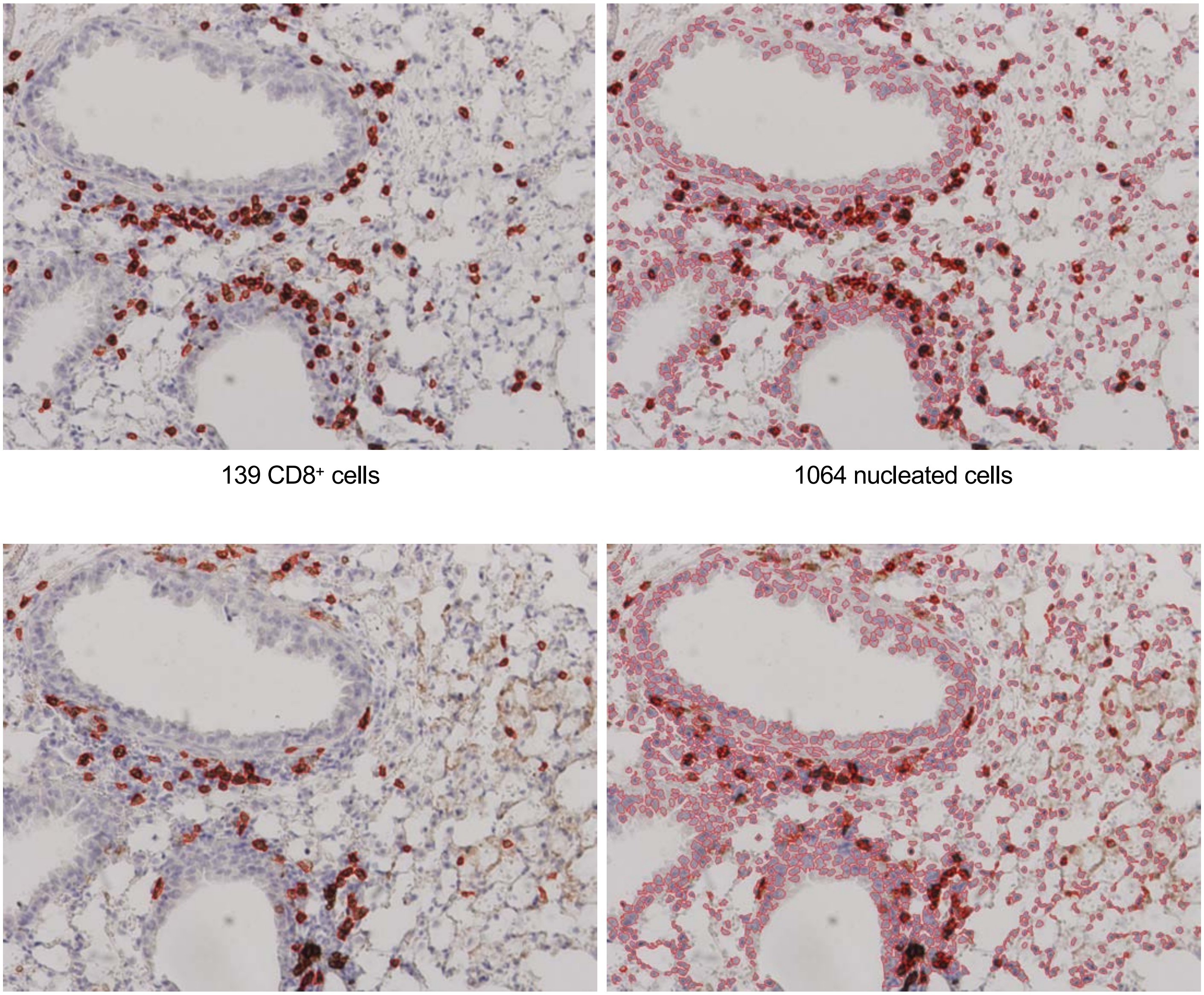

CD8$^+$

139 CD8$^+$ cells

1064 nucleated cells

CD4$^+$

85 CD4$^+$ cells

1165 nucleated cells

Supplementary Figure 7

PBS Lung 1-1
2 dpi

CD8^{+}/CD4^{+} Cell Annotations

Nucleated Cell Annotations

CD8^{+}

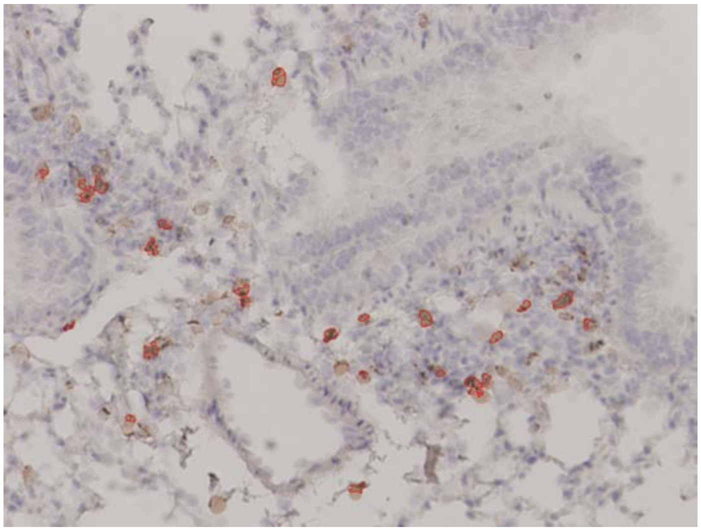

25 CD8^{+} cells

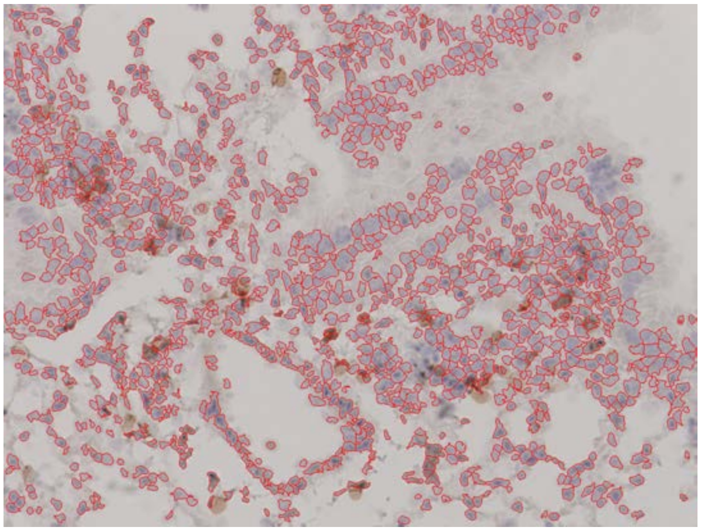

1165 nucleated cells

CD4^{+}

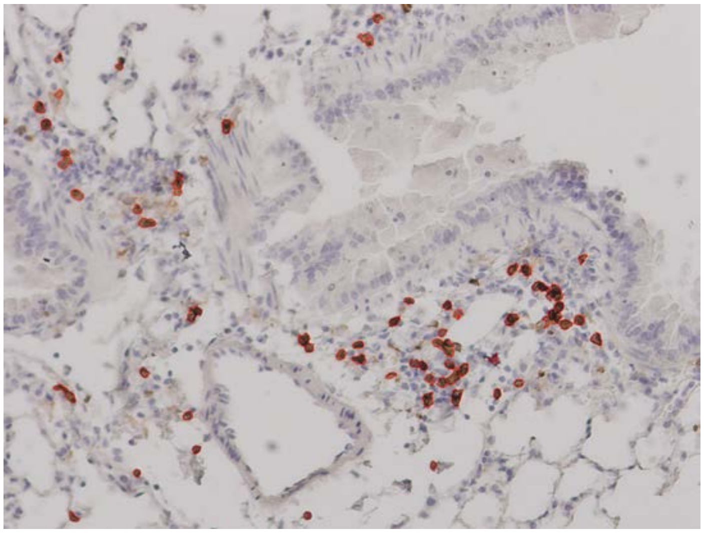

63 CD4^{+} cells

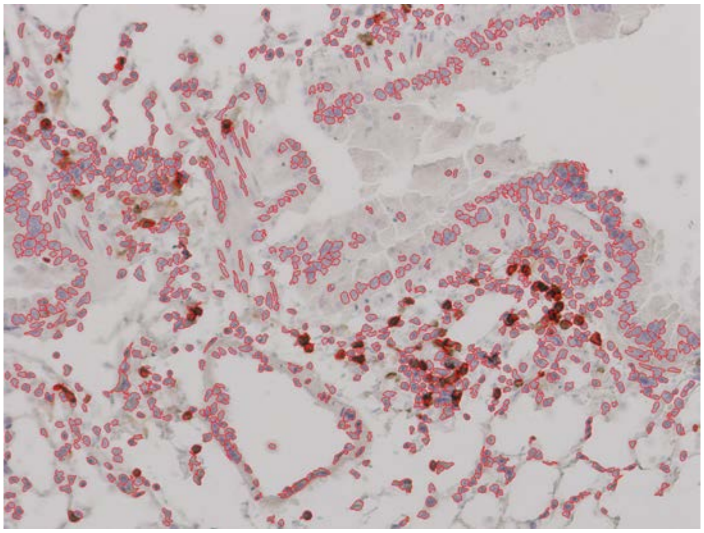

952 nucleated cells

Supplementary Figure 7

PBS Lung 2-1
2 dpi

CD8$^+$/CD4$^+$ Cell Annotations

Nucleated Cell Annotations

CD8$^+$

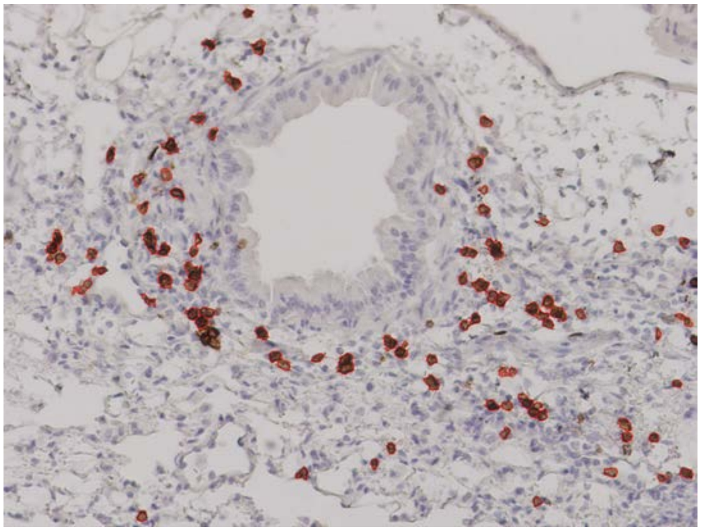

85 CD8$^+$ cells

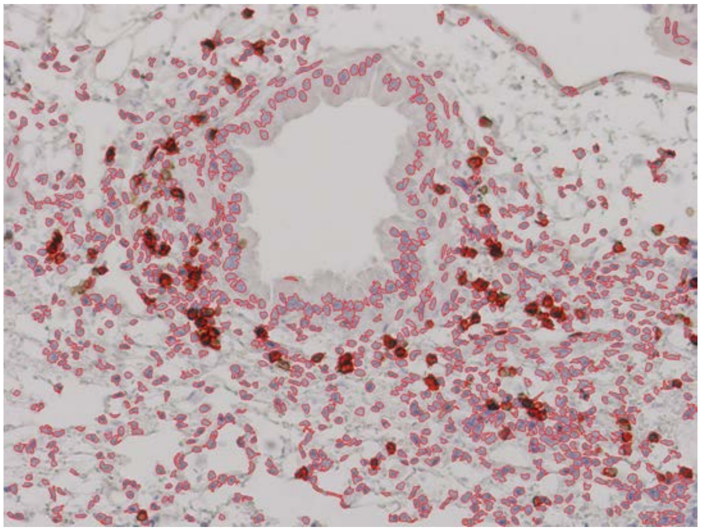

1166 nucleated cells

CD4$^+$

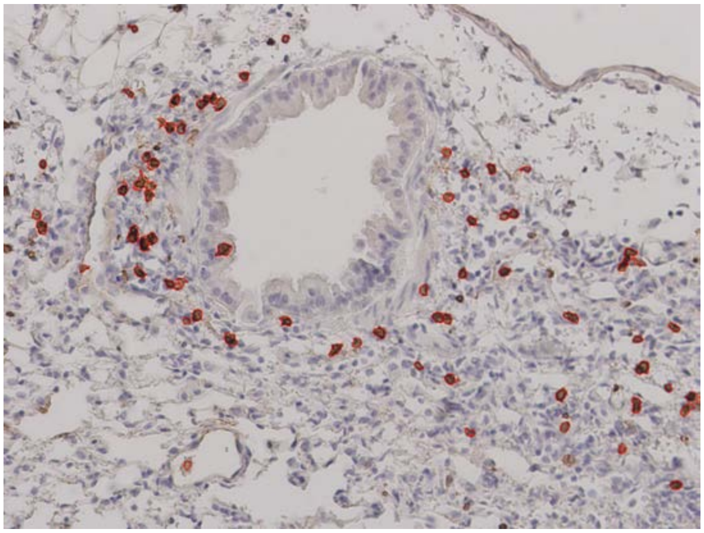

69 CD4$^+$ cells

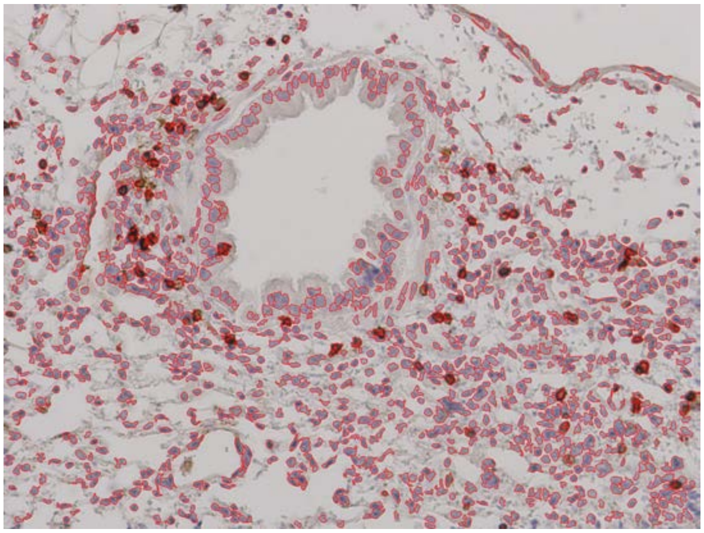

1229 nucleated cells

PBS Lung 2-2
2 dpi

CD8$^+$/CD4$^+$ Cell Annotations | Nucleated Cell Annotations

CD8$^+$

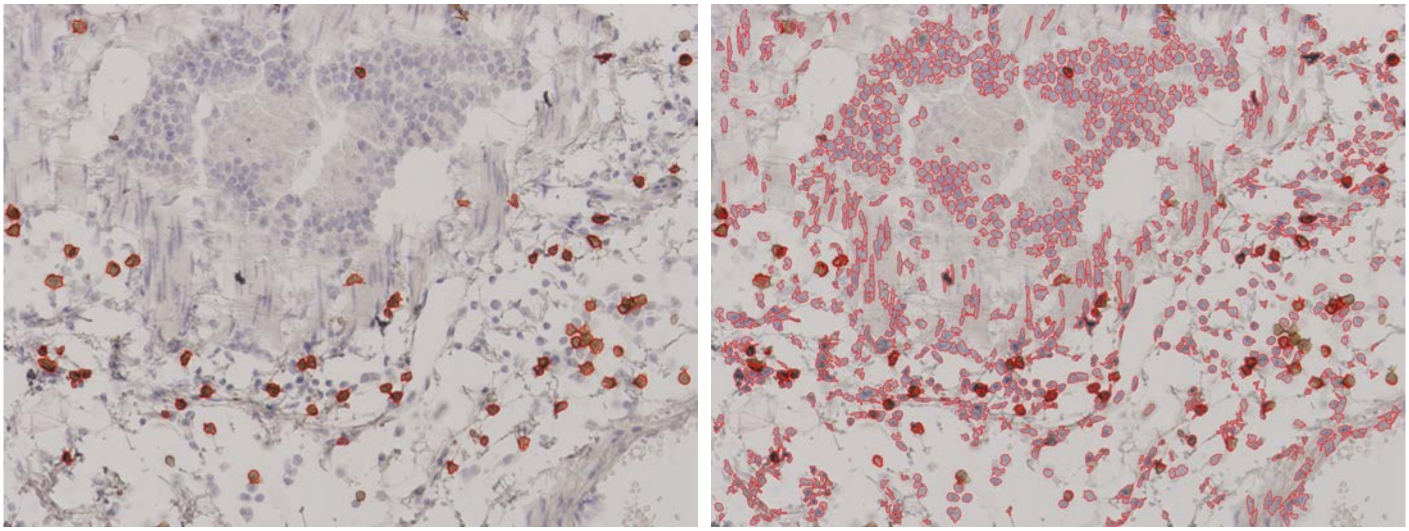

62 CD8$^+$ cells | 1126 nucleated cells

CD4$^+$

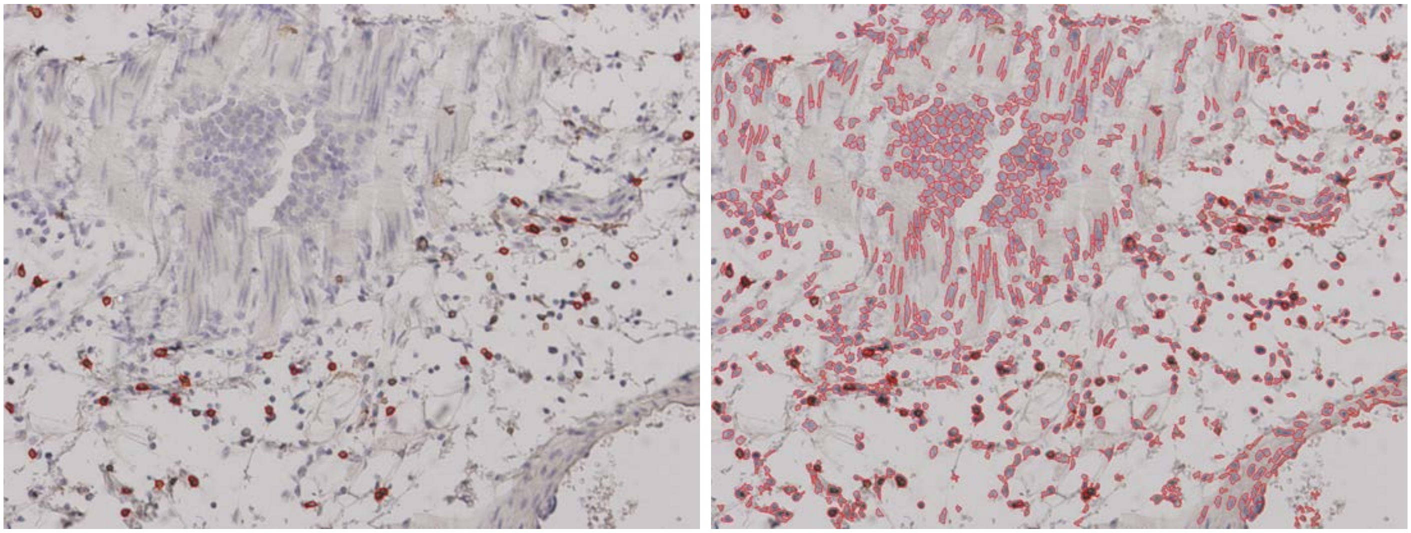

37 CD4$^+$ cells | 1034 nucleated cells

Supplementary Figure 7

PBS Lung 3-1
2 dpi

	CD8$^+$/CD4$^+$ Cell Annotations	Nucleated Cell Annotations

CD8$^+$

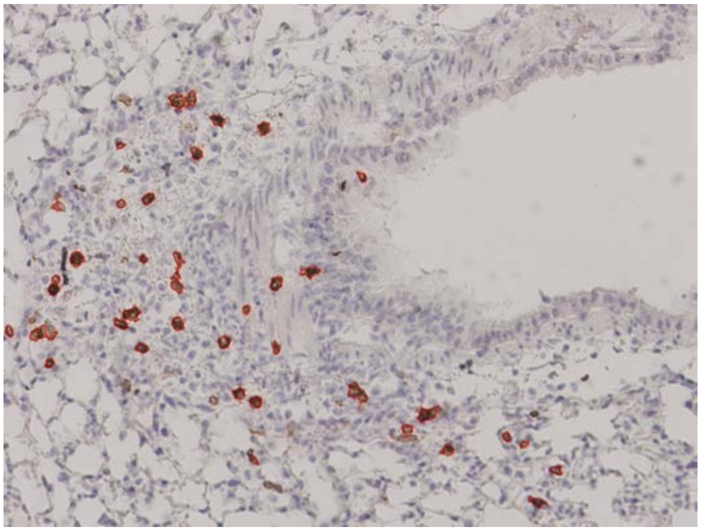

42 CD8$^+$ cells

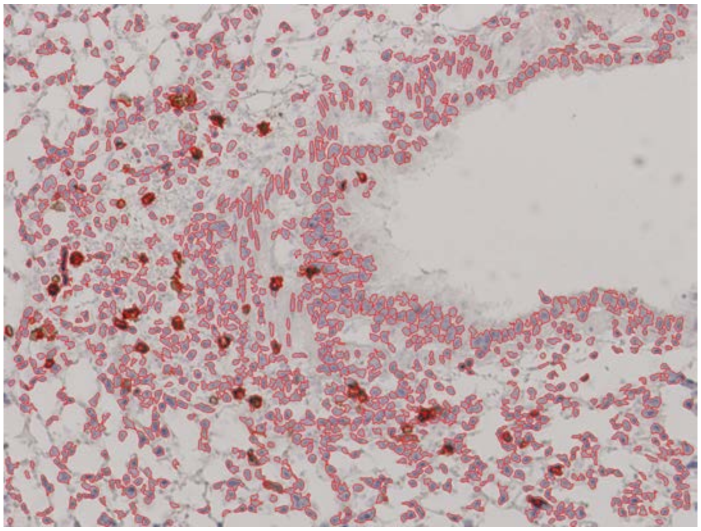

1259 nucleated cells

CD4$^+$

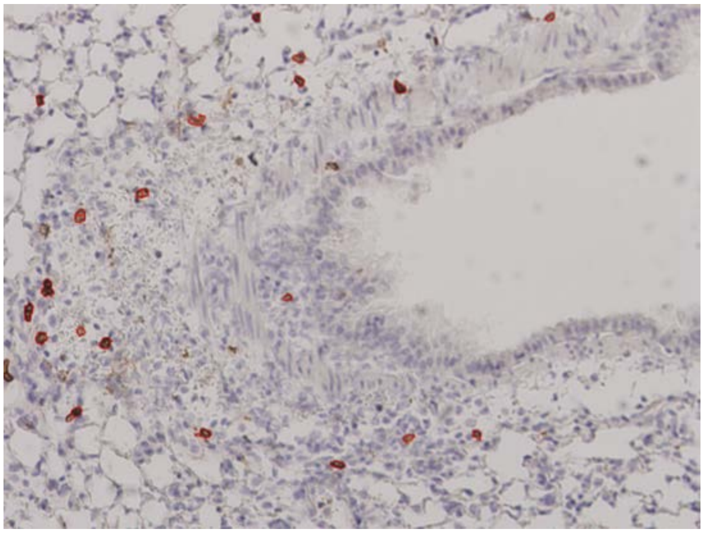

23 CD4$^+$ cells

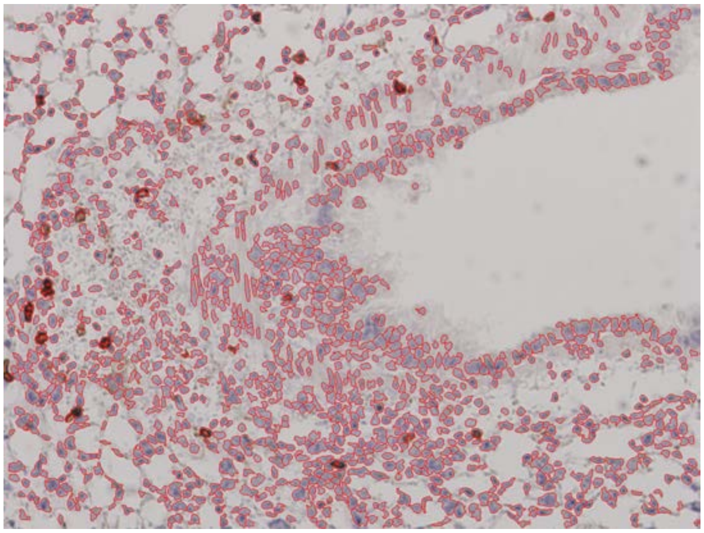

1241 nucleated cells

Supplementary Figure 7
Page 15/29

PBS Lung 3-2
2 dpi

CD8⁺/CD4⁺ Cell Annotations | Nucleated Cell Annotations

CD8⁺

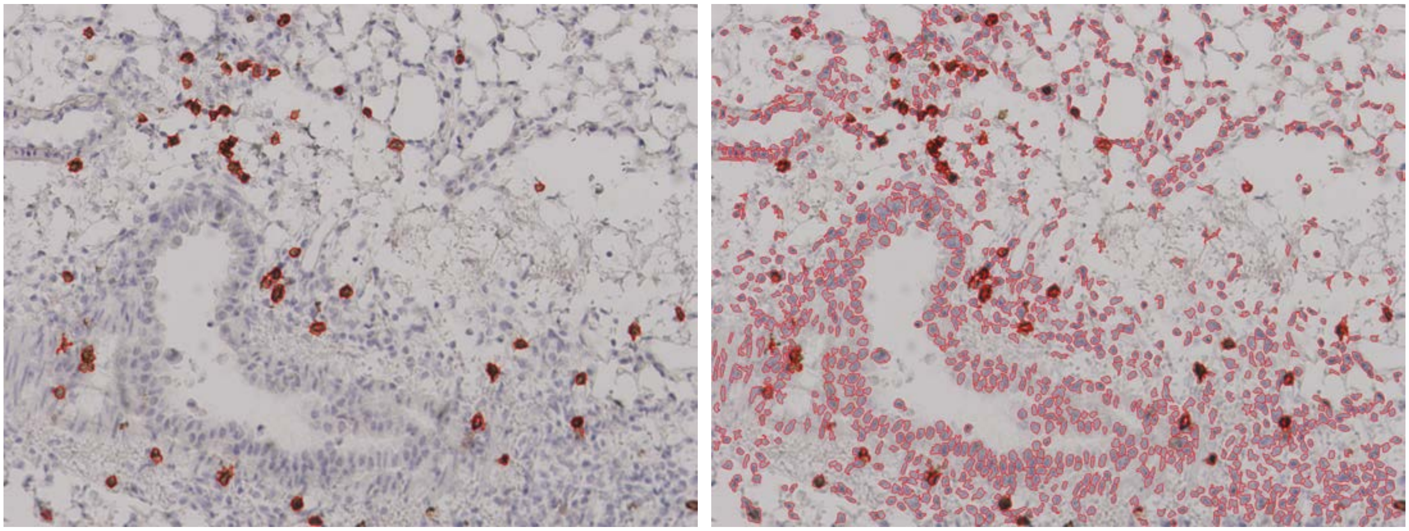

50 CD8⁺ cells | 1208 nucleated cells

CD4⁺

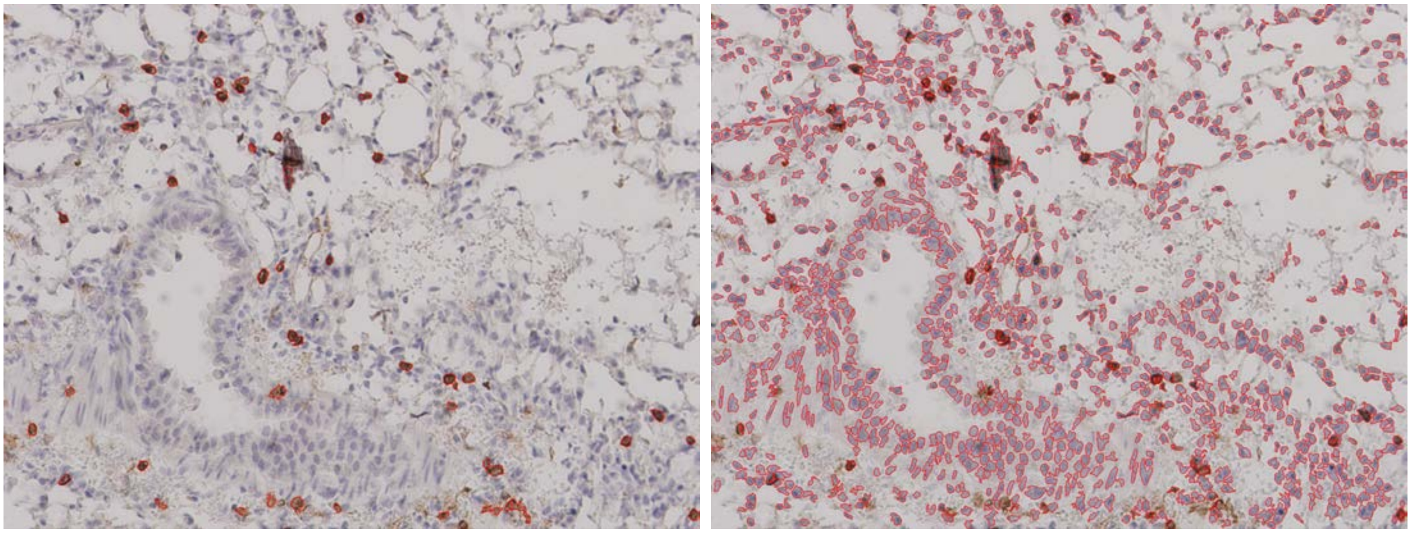

48 CD4⁺ cells | 1205 nucleated cells

Supplementary Figure 7

MIT-T-COVID Lung 1-1
7 dpi

CD8+/CD4+ Cell Annotations | Nucleated Cell Annotations

CD8+

CD4+

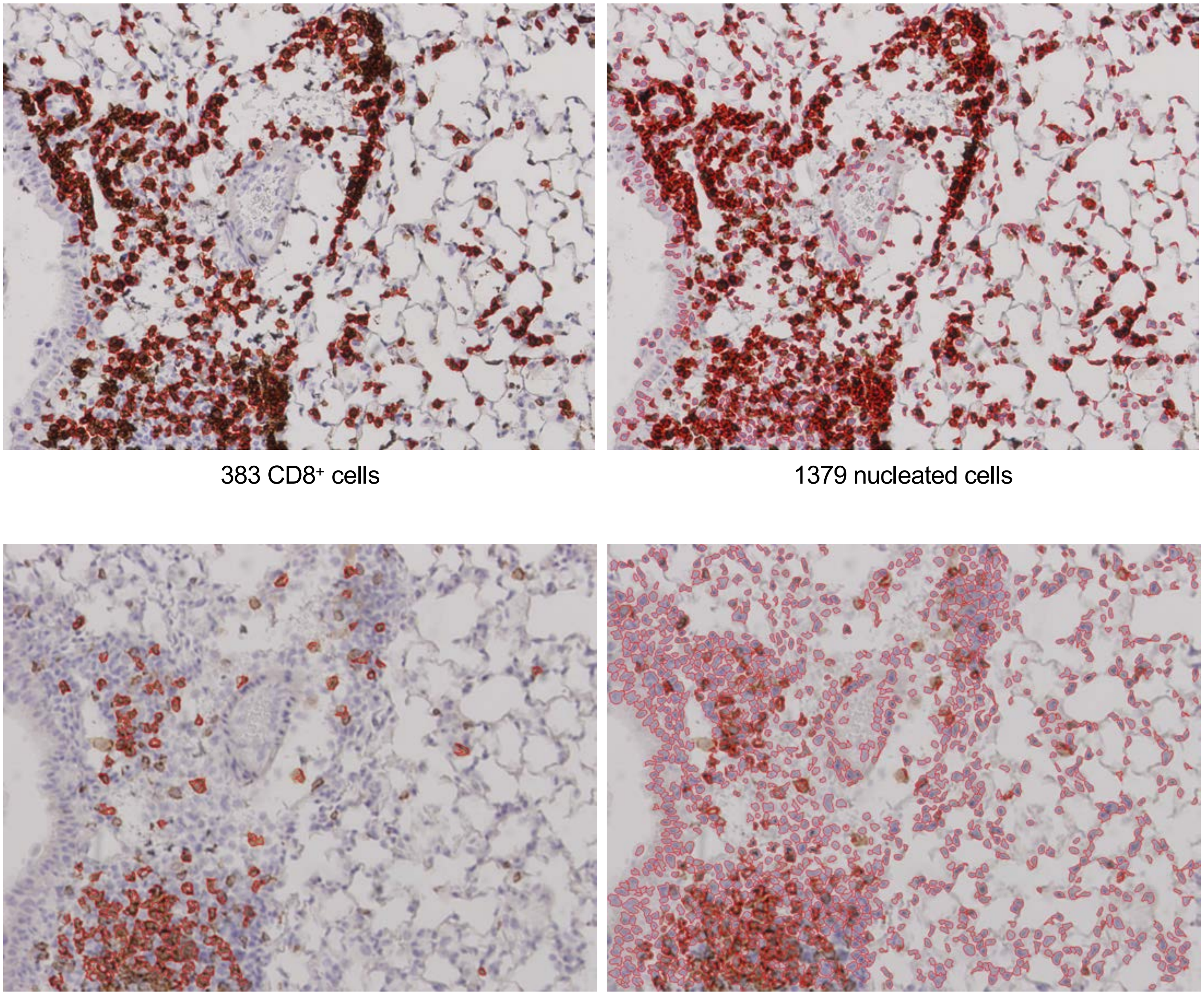

383 CD8+ cells | 1379 nucleated cells

139 CD4+ cells | 1424 nucleated cells

Supplementary Figure 7
Page 17/29

MIT-T-COVID Lung 1-2
7 dpi

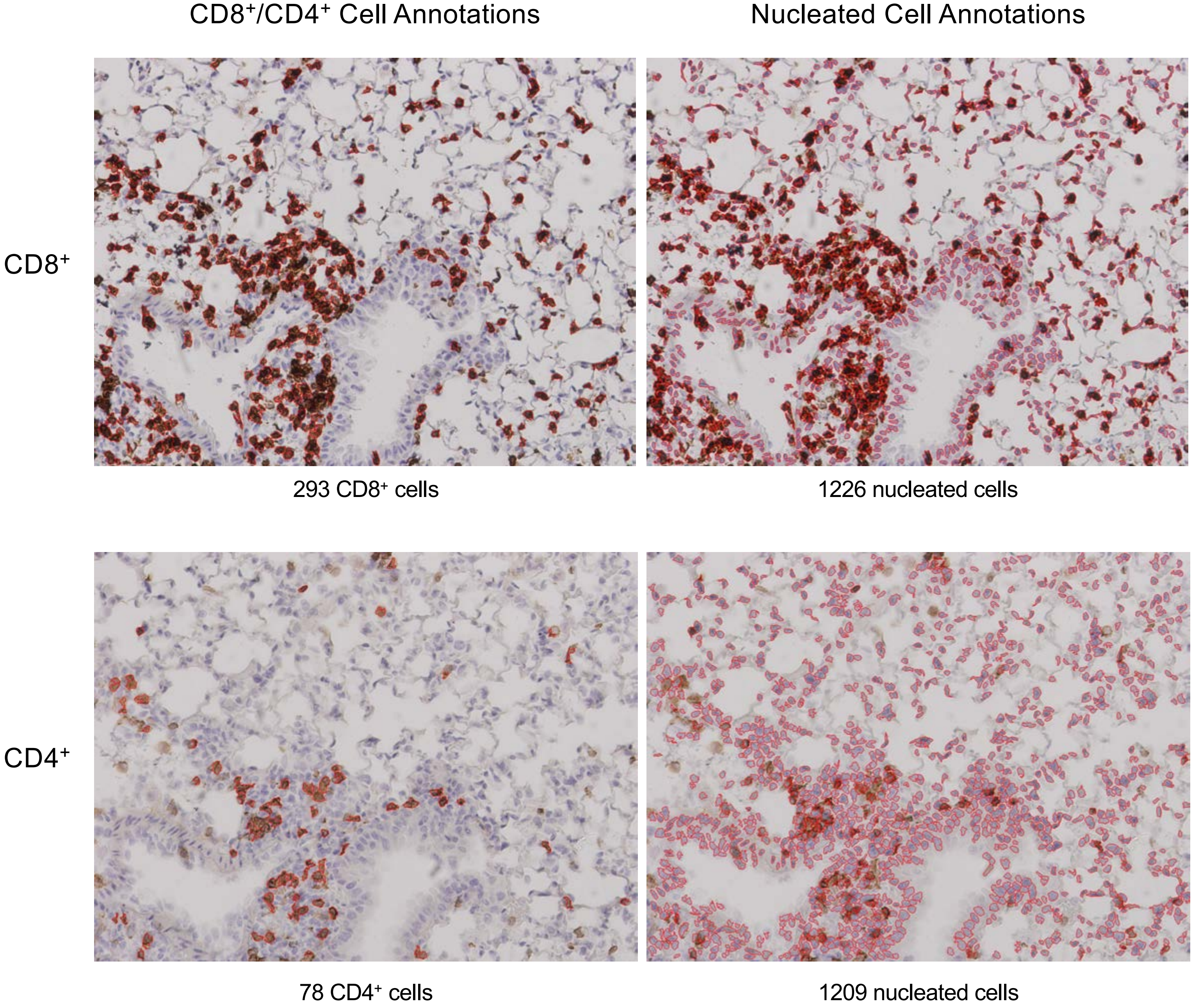

Supplementary Figure 7

MIT-T-COVID Lung 2-1
7 dpi

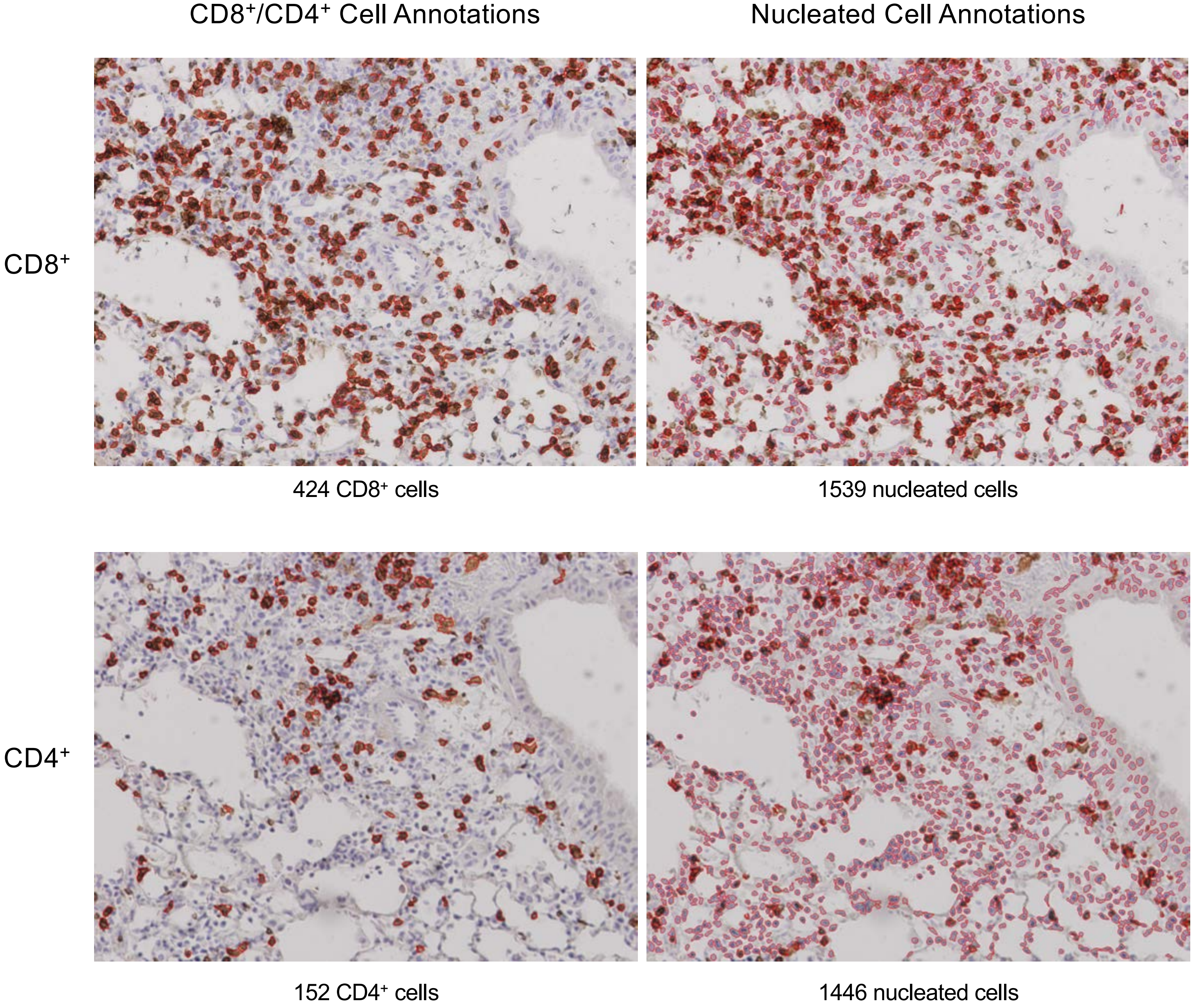

Supplementary Figure 7

MIT-T-COVID Lung 2-2
7 dpi

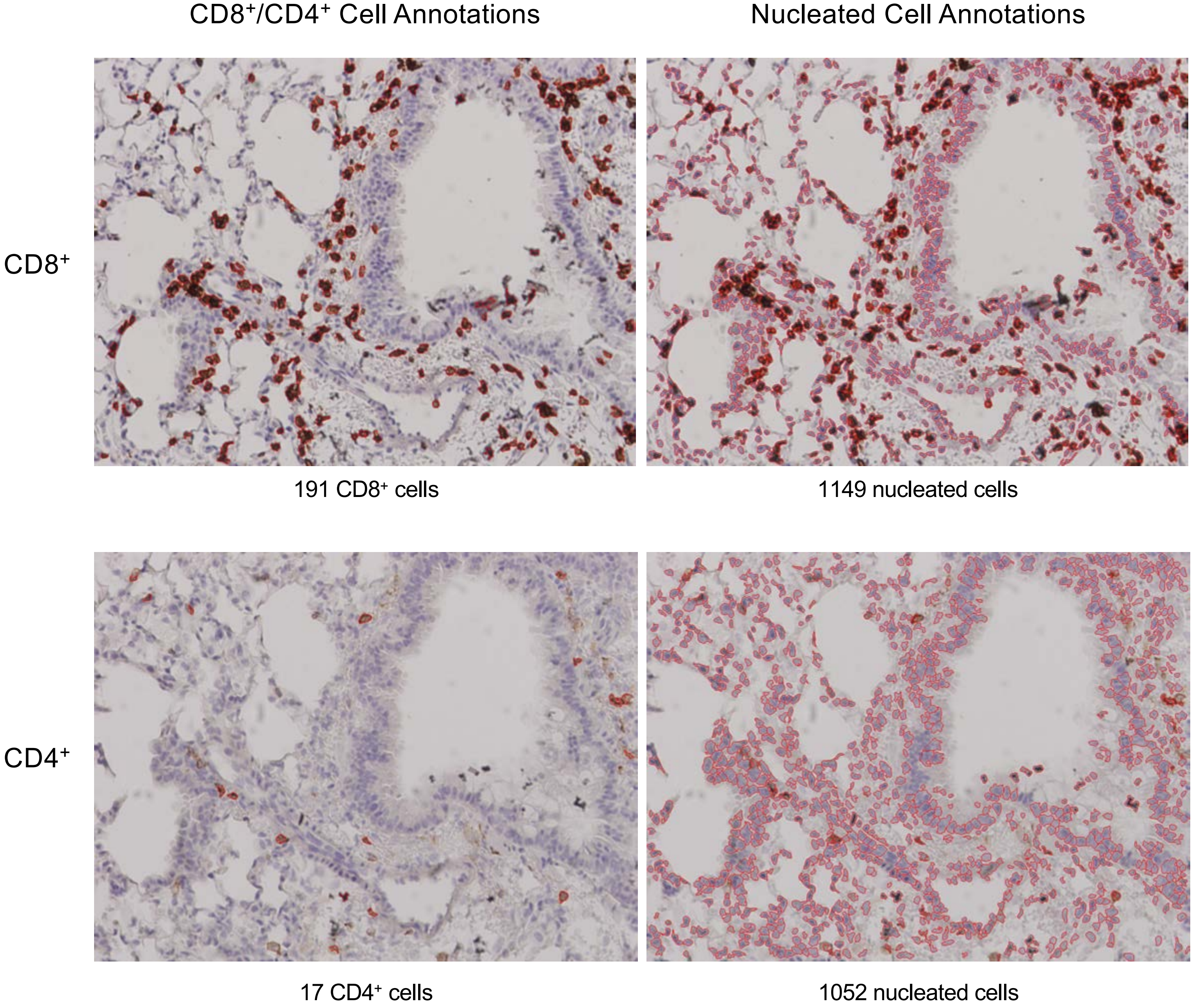

Supplementary Figure 7
Page 20/29

Pfizer/BNT Lung 1-1
7 dpi

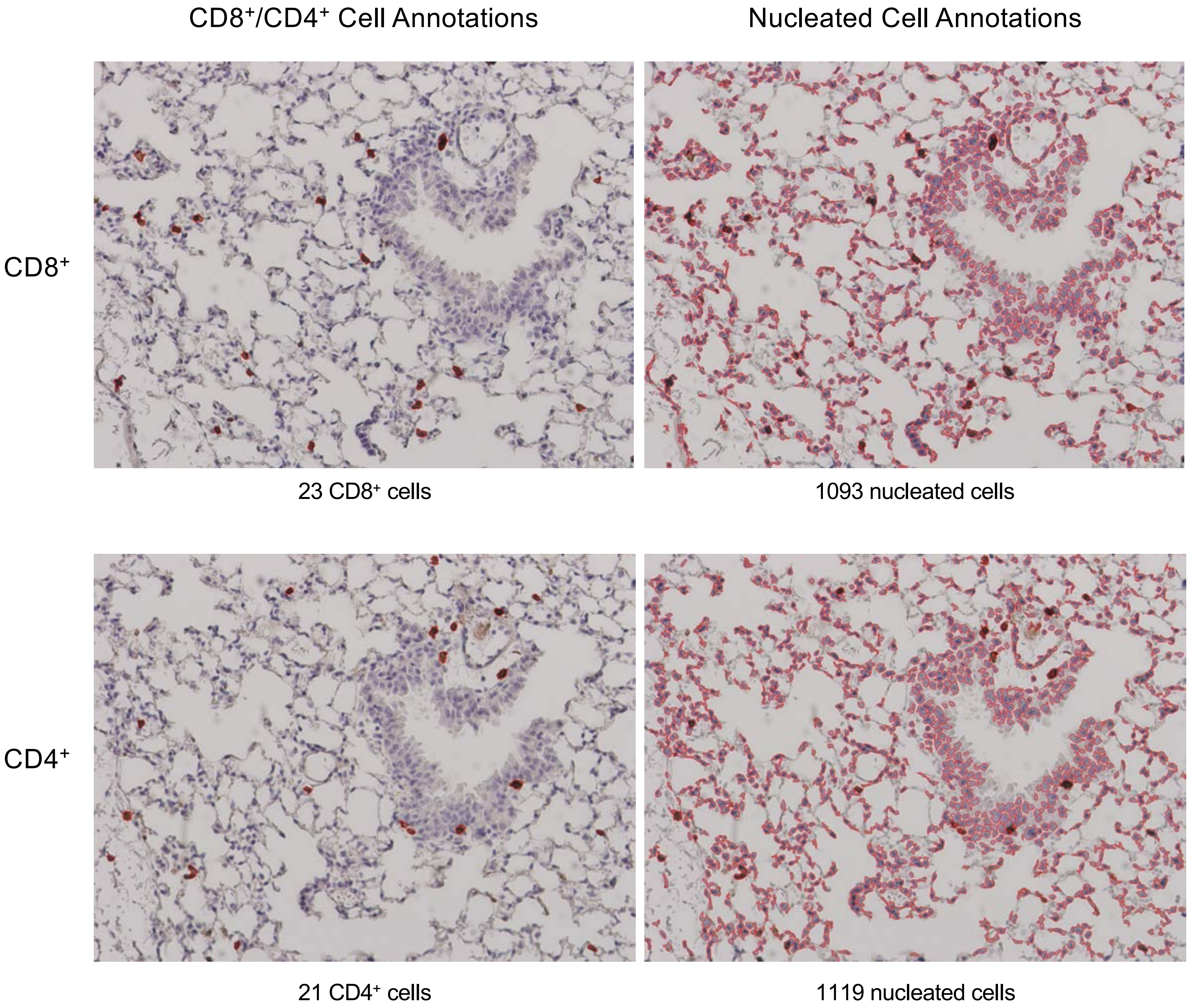

Supplementary Figure 7
Page 21/29

Pfizer/BNT Lung 1-2
7 dpi

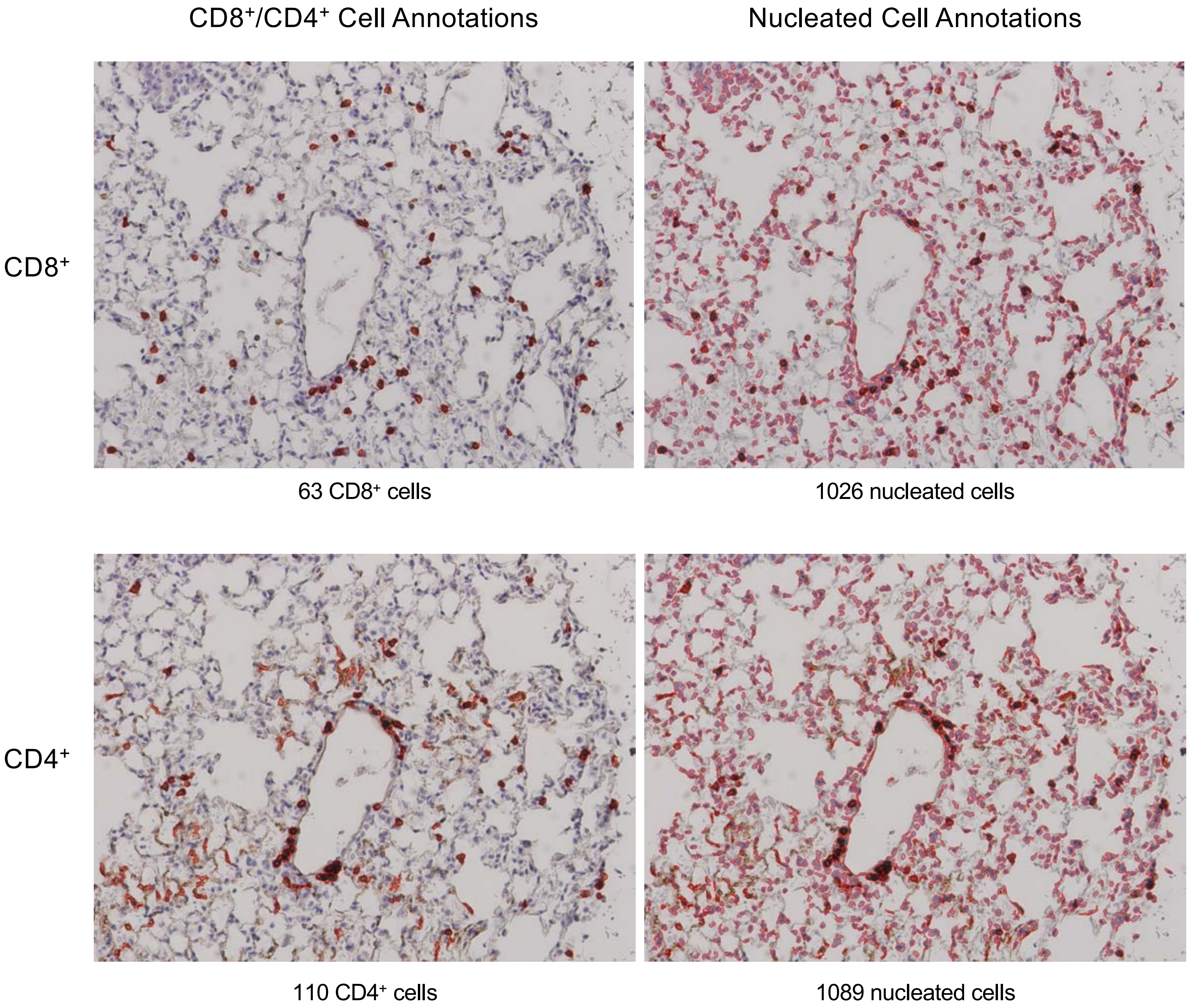

Supplementary Figure 7
Page 22/29

Pfizer/BNT Lung 1-3
7 dpi

CD8⁺/CD4⁺ Cell Annotations

Nucleated Cell Annotations

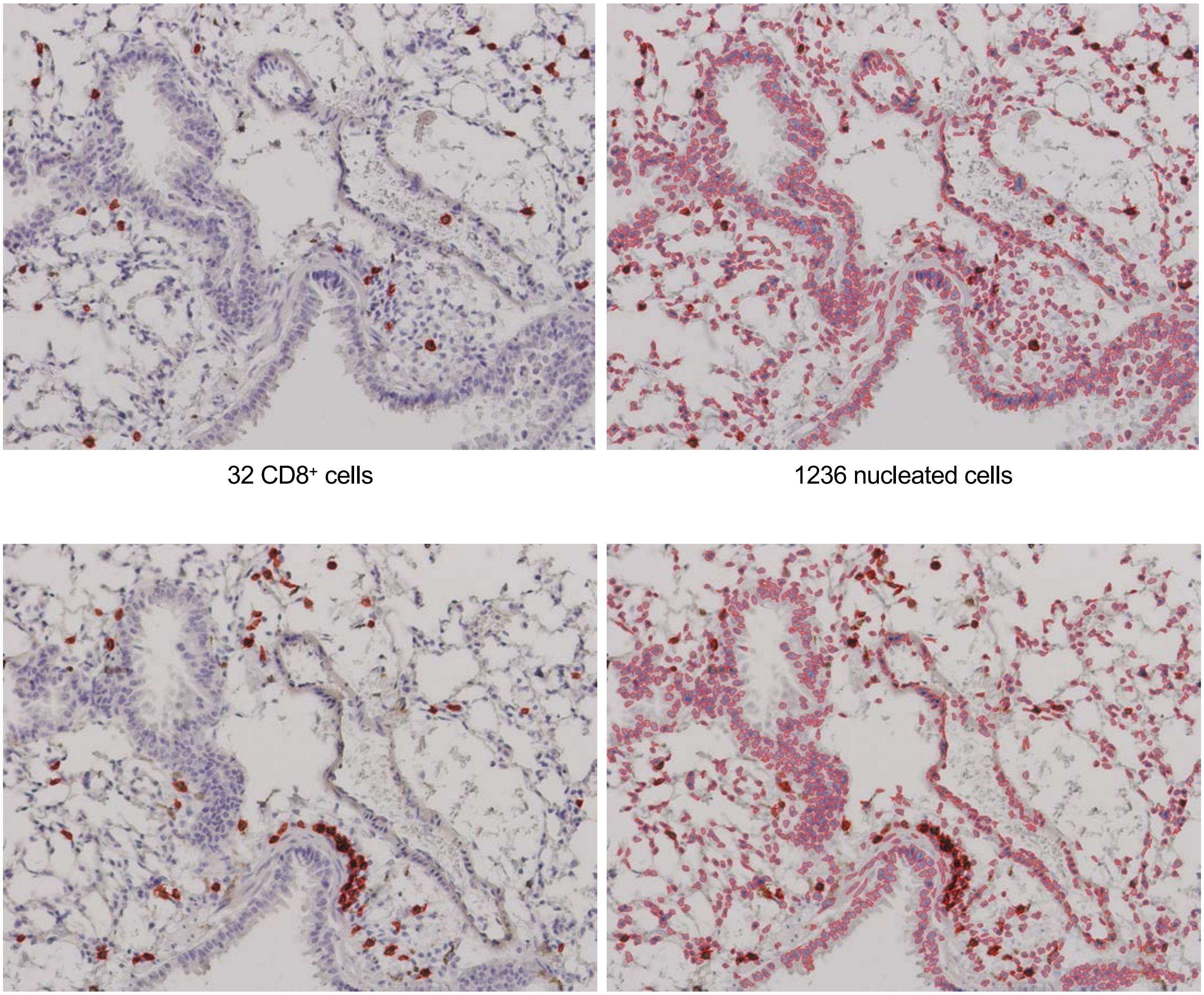

CD8⁺

32 CD8⁺ cells

1236 nucleated cells

CD4⁺

63 CD4⁺ cells

1153 nucleated cells

Supplementary Figure 7
Page 23/29

Pfizer/BNT Lung 2-1
7 dpi

CD8^{+}/CD4^{+} Cell Annotations

Nucleated Cell Annotations

CD8^{+}

CD4^{+}

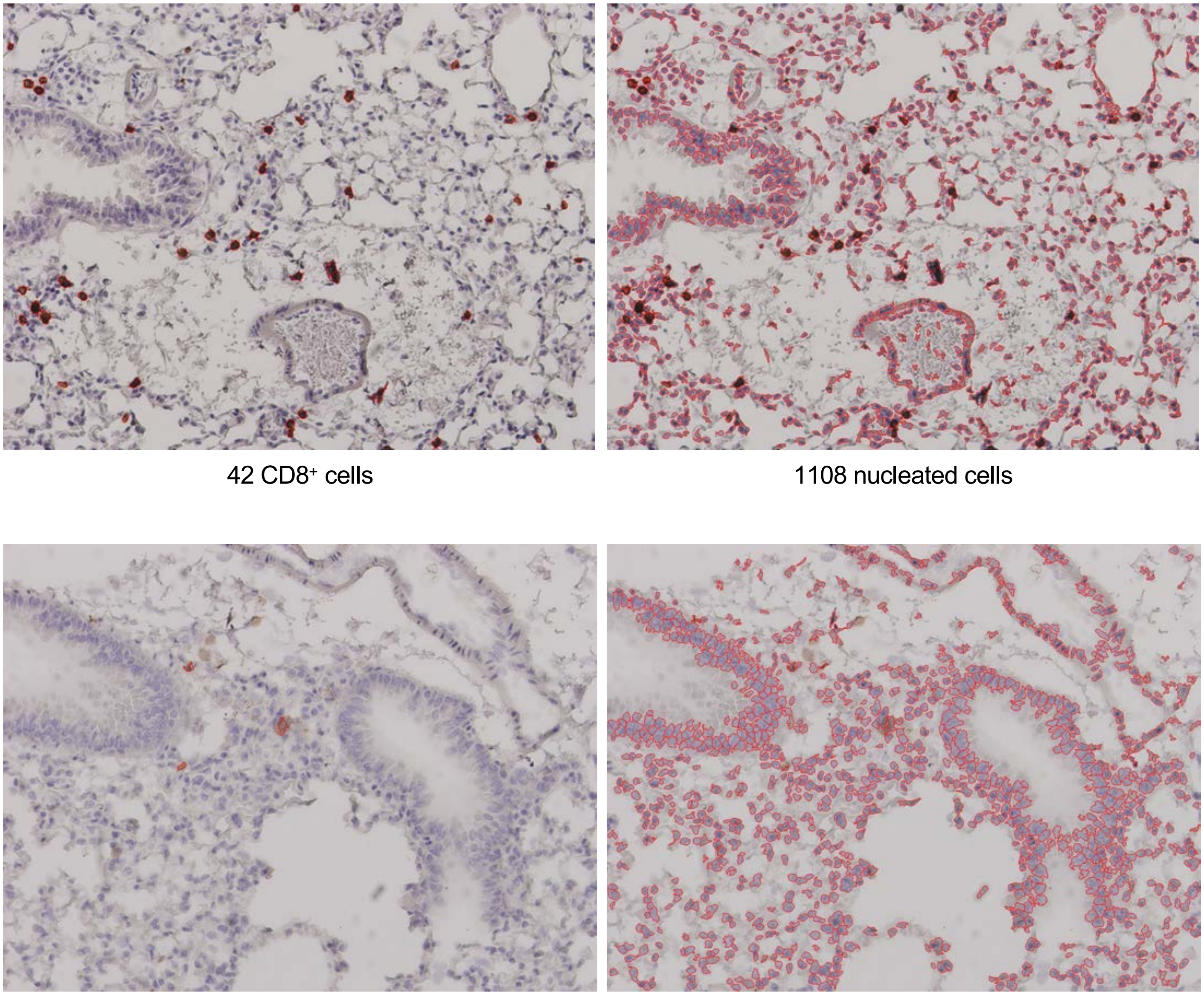

42 CD8^{+} cells

1108 nucleated cells

3 CD4^{+} cells

1036 nucleated cells

Pfizer/BNT Lung 3-1
7 dpi

CD8$^+$/CD4$^+$ Cell Annotations | Nucleated Cell Annotations

CD8$^+$

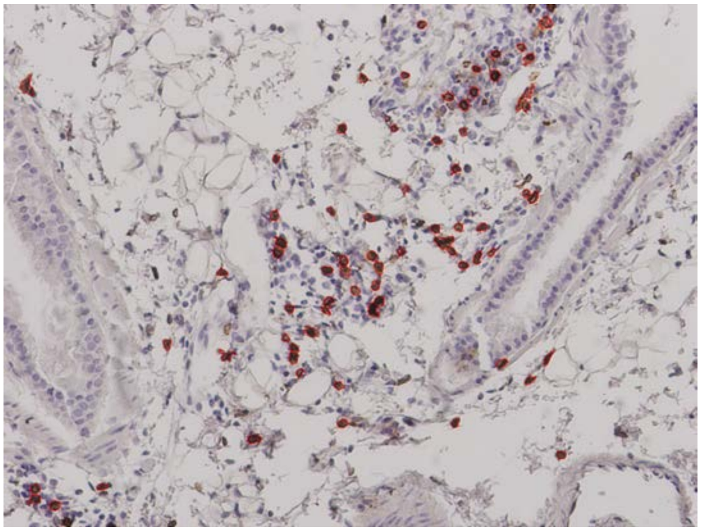

69 CD8$^+$ cells

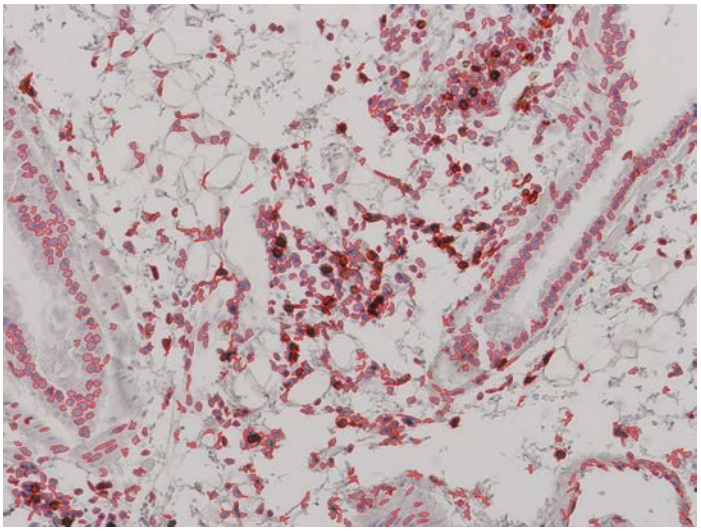

969 nucleated cells

CD4$^+$

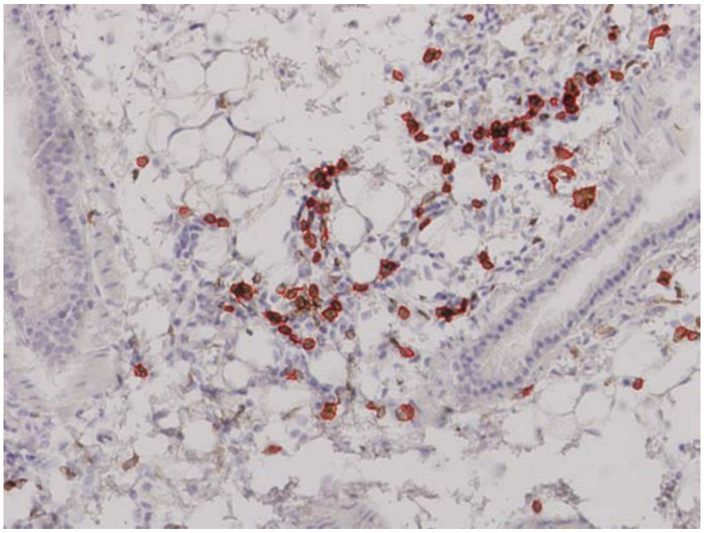

81 CD4$^+$ cells

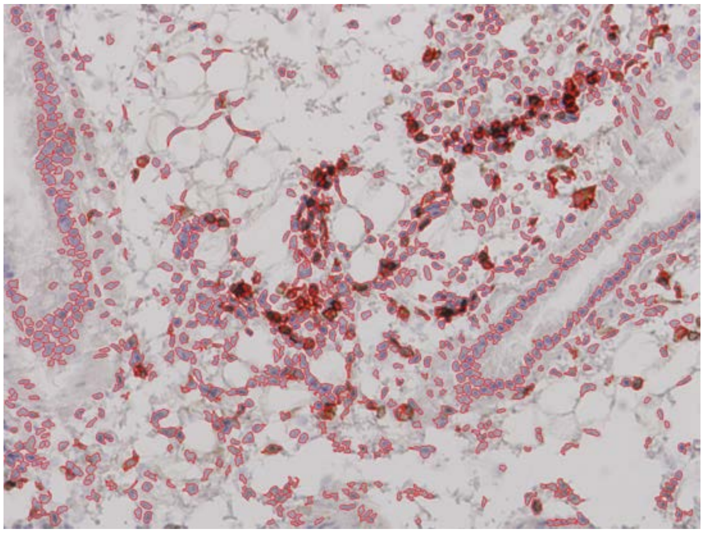

1043 nucleated cells

PBS Lung 1-1
7 dpi

CD8$^+$/CD4$^+$ Cell Annotations | Nucleated Cell Annotations

CD8$^+$

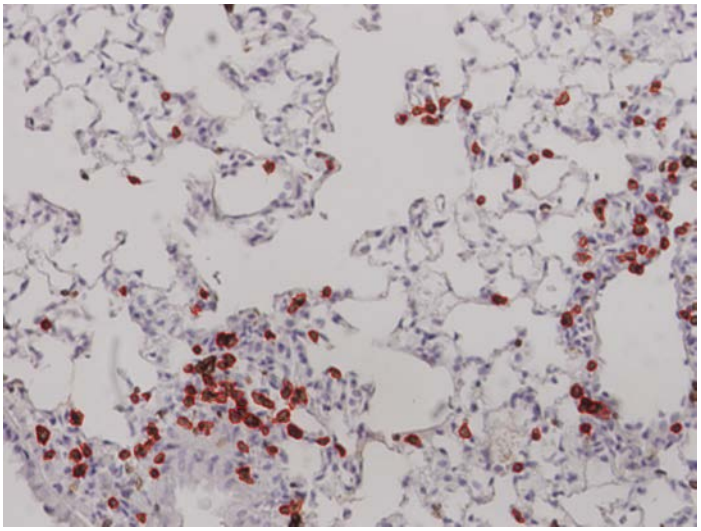

108 CD8$^+$ cells

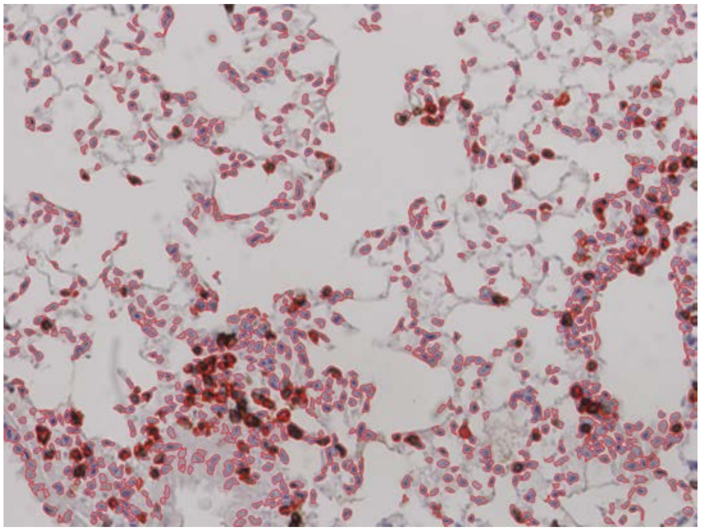

912 nucleated cells

CD4$^+$

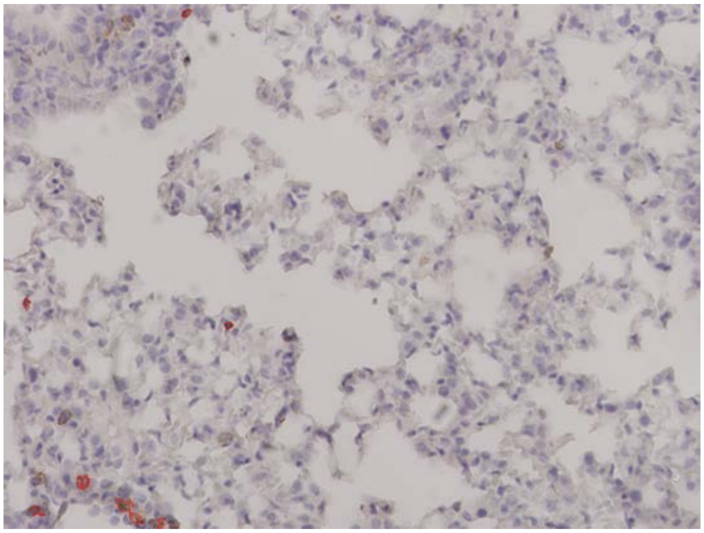

9 CD4$^+$ cells

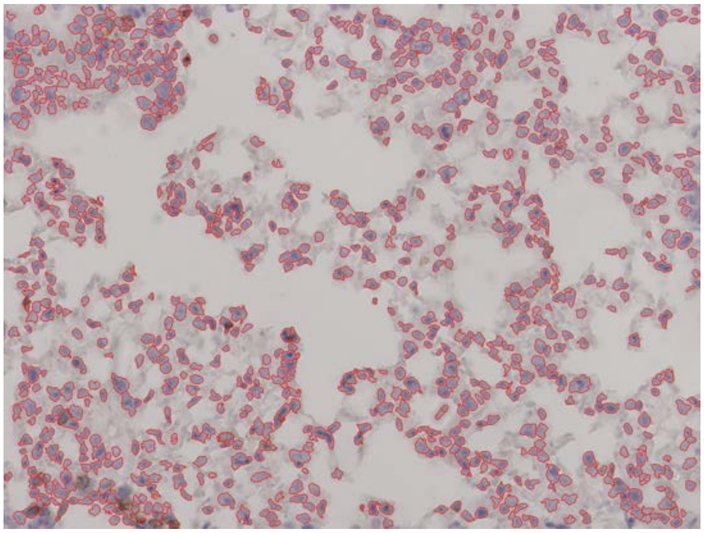

892 nucleated cells

Supplementary Figure 7

PBS Lung 2-1
7 dpi

CD8$^+$/CD4$^+$ Cell Annotations

Nucleated Cell Annotations

CD8$^+$

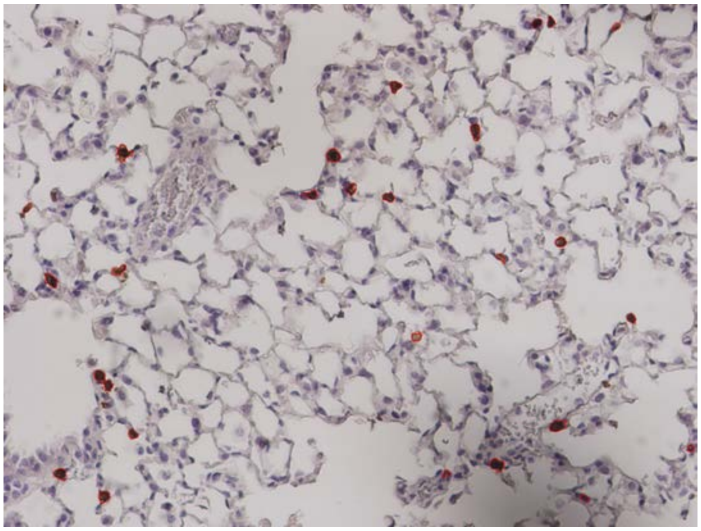

27 CD8$^+$ cells

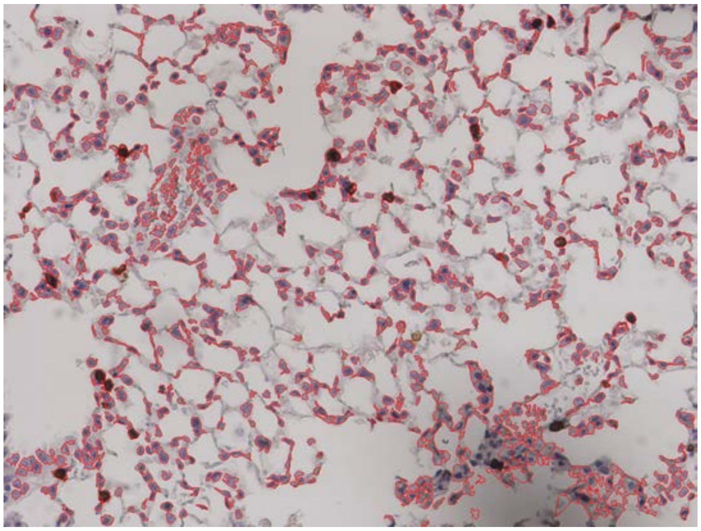

958 nucleated cells

CD4$^+$

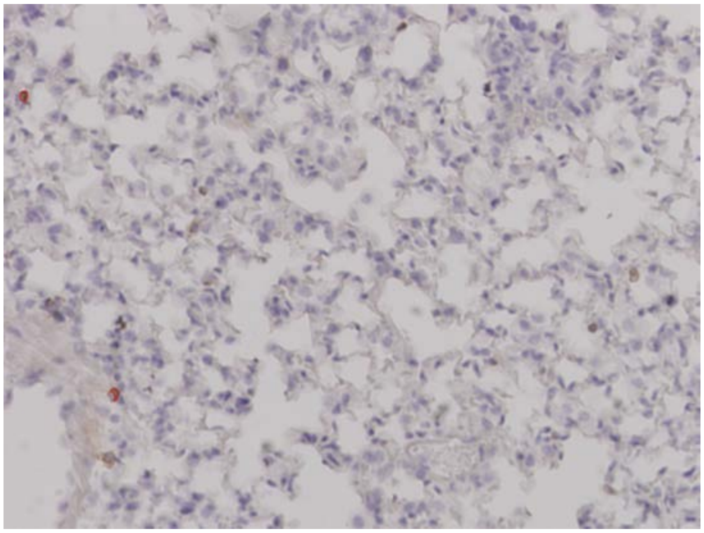

2 CD4$^+$ cells

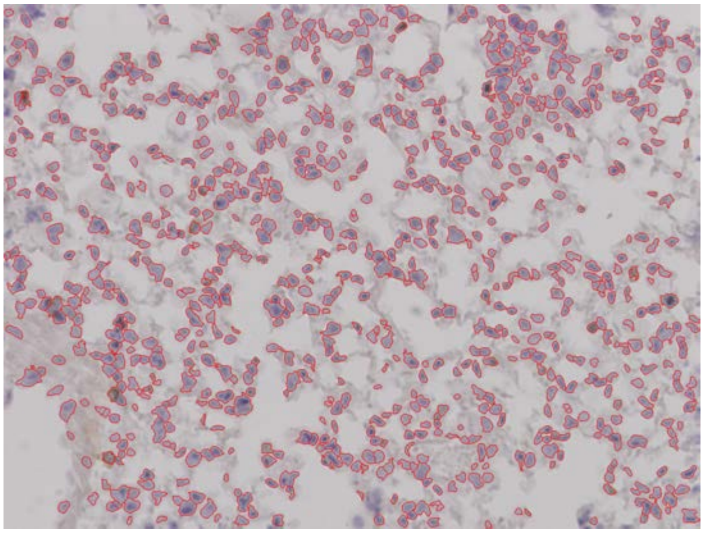

813 nucleated cells

Supplementary Figure 7

PBS Lung 2-2
7 dpi

CD8$^+$/CD4$^+$ Cell Annotations

Nucleated Cell Annotations

CD8$^+$

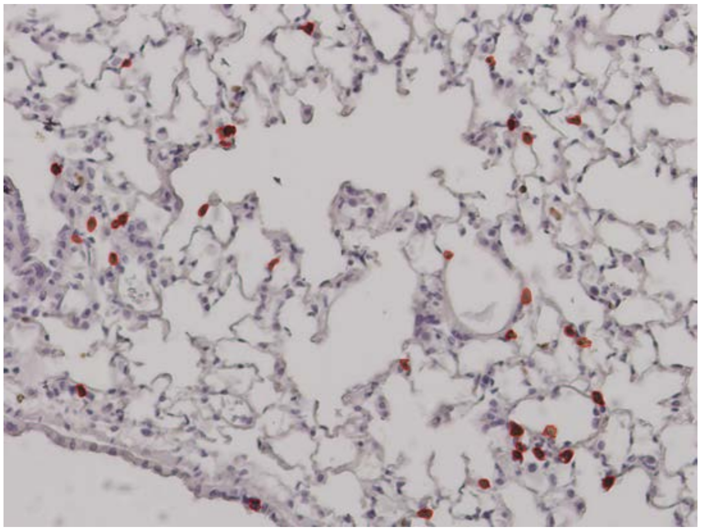

34 CD8$^+$ cells

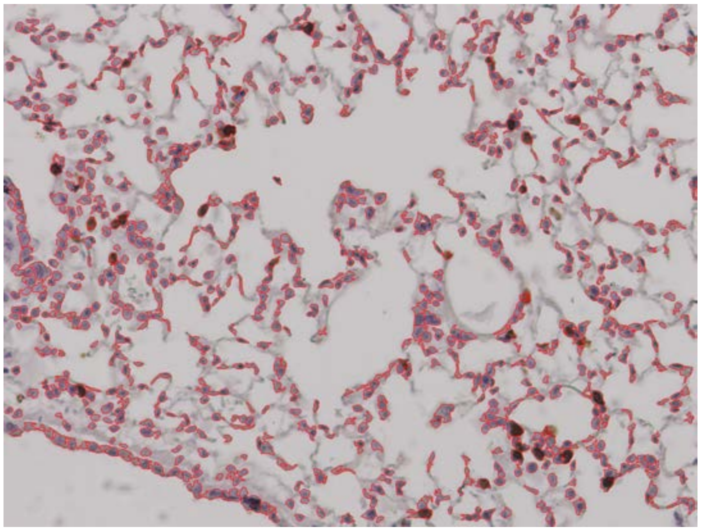

869 nucleated cells

CD4$^+$

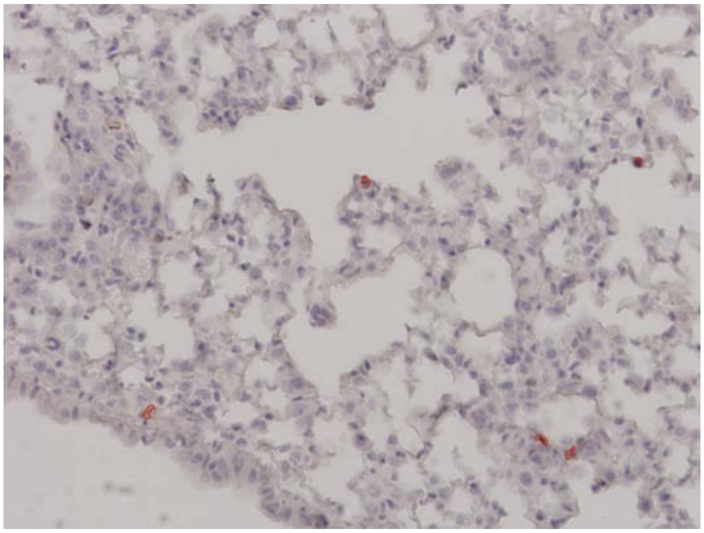

5 CD4$^+$ cells

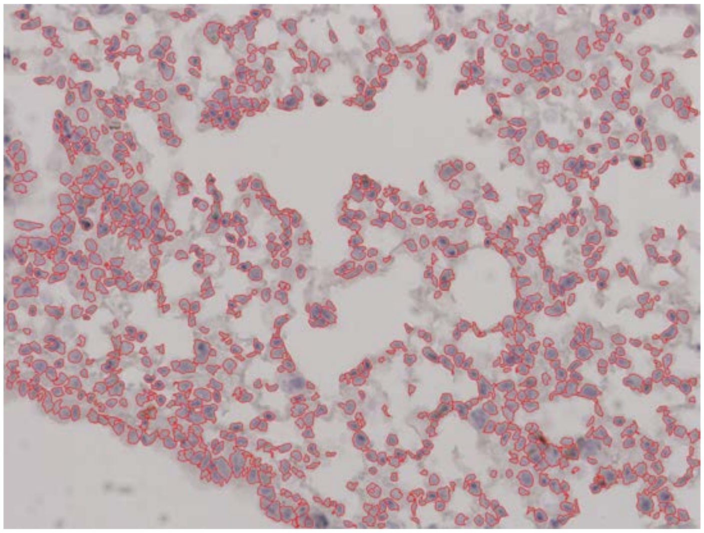

937 nucleated cells

Supplementary Figure 7

PBS Lung 4-1
7 dpi

CD8$^+$/CD4$^+$ Cell Annotations | Nucleated Cell Annotations

CD8$^+$

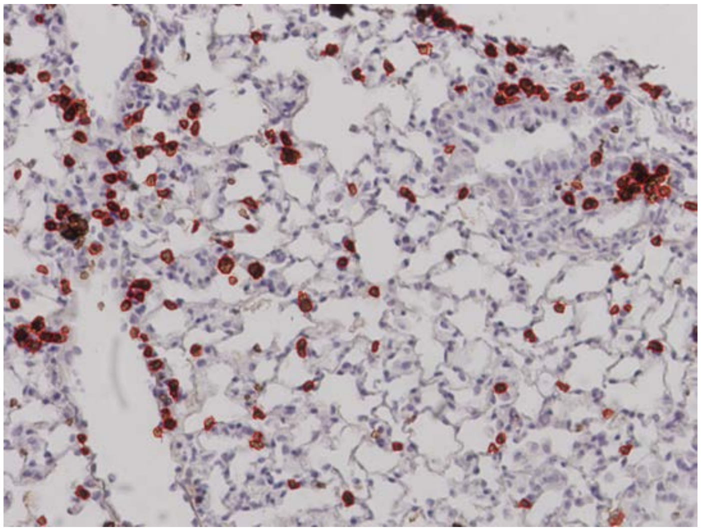

148 CD8$^+$ cells

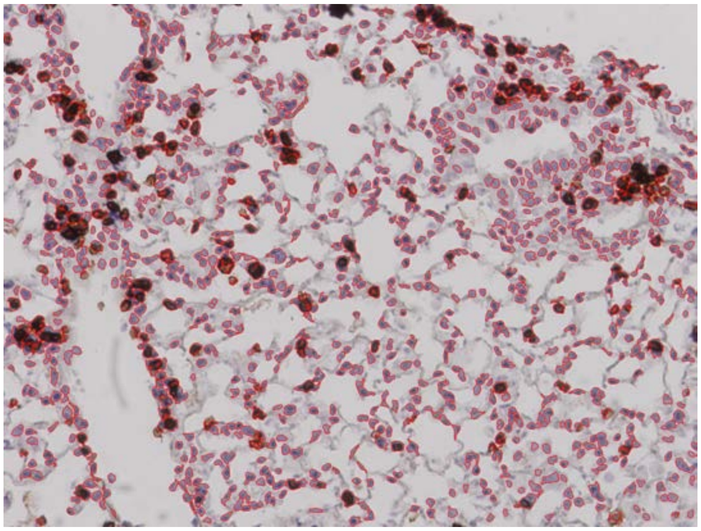

1111 nucleated cells

CD4$^+$

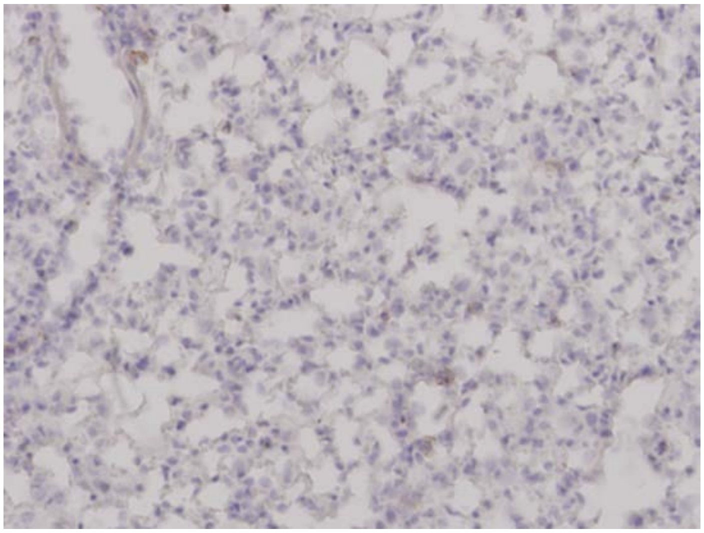

0 CD4$^+$ cells

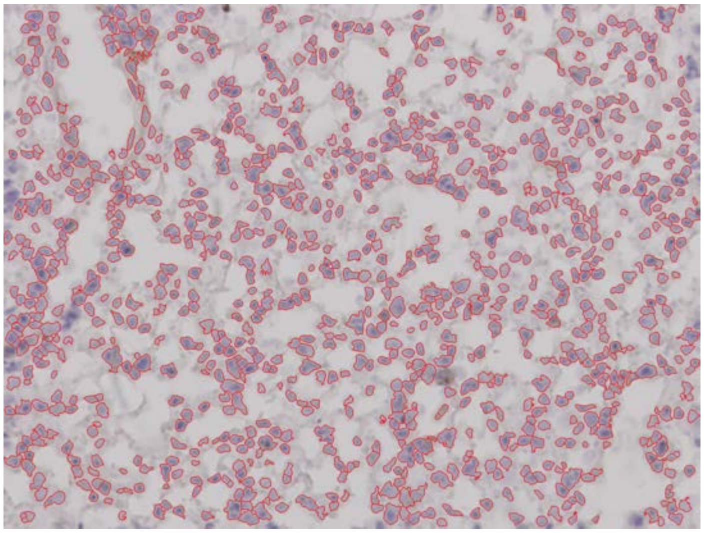

981 nucleated cells

Supplementary Figure 7
Page 29/29

PBS Lung 5-1
7 dpi

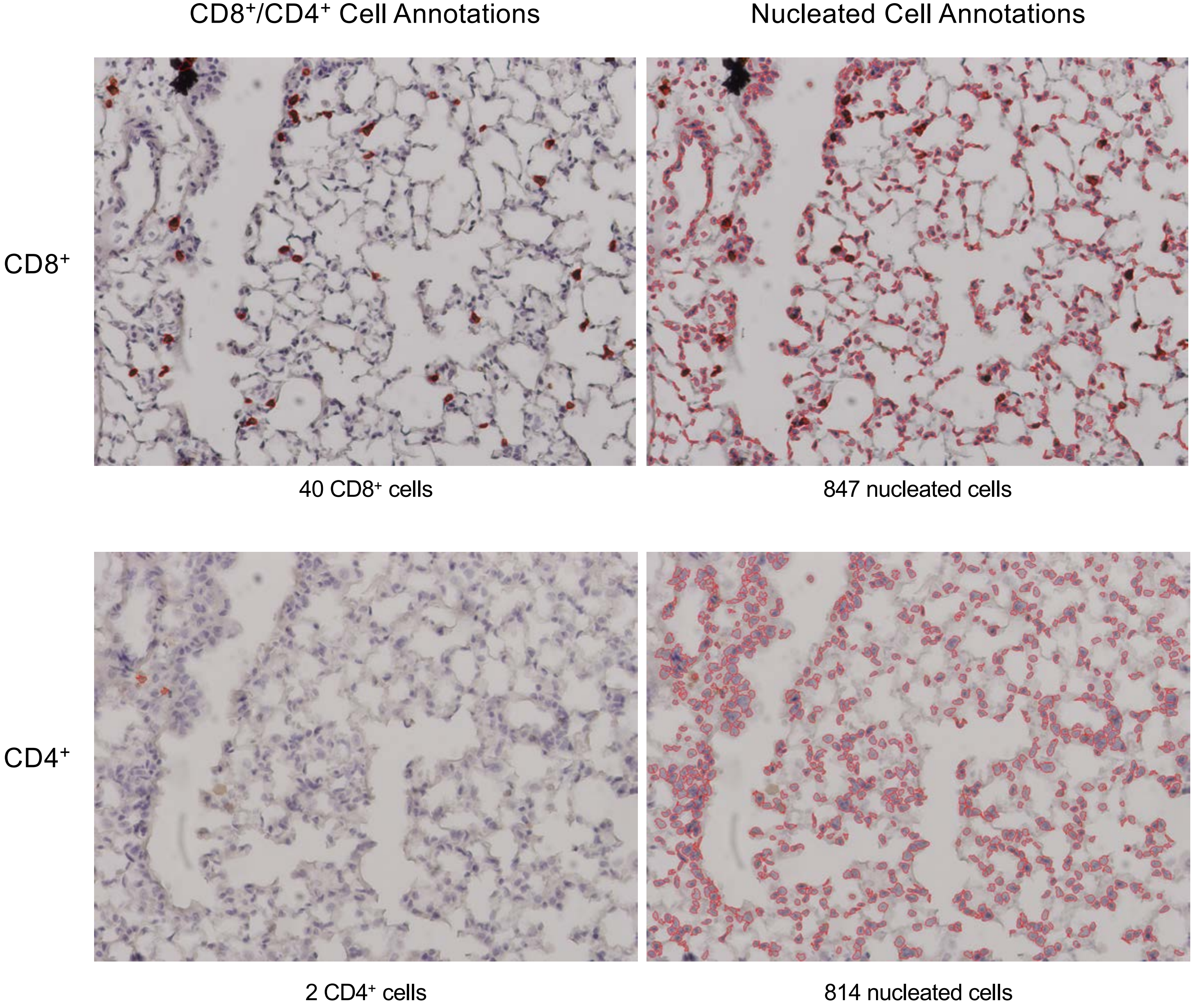

Supplement: Supplementary file 2 [file DataSheet_2.pdf]
