# Supplementary material for: A pan-variant mRNA-LNP T cell vaccine protects HLA transgenic mice from mortality after infection with SARS-CoV-2 Beta
Source: Front Immunol. 2023 Mar 9;14:1135815. doi: 10.3389/fimmu.2023.1135815 (PMC10033589; doi:10.3389/fimmu.2023.1135815)

Supplementary Figure 11
Page 1/30

MIT-T-COVID Lung 1-1
Unchallenged, Female Cohort

CD8$^+$/CD4$^+$ Cell Annotations | Nucleated Cell Annotations

CD8$^+$

CD4$^+$

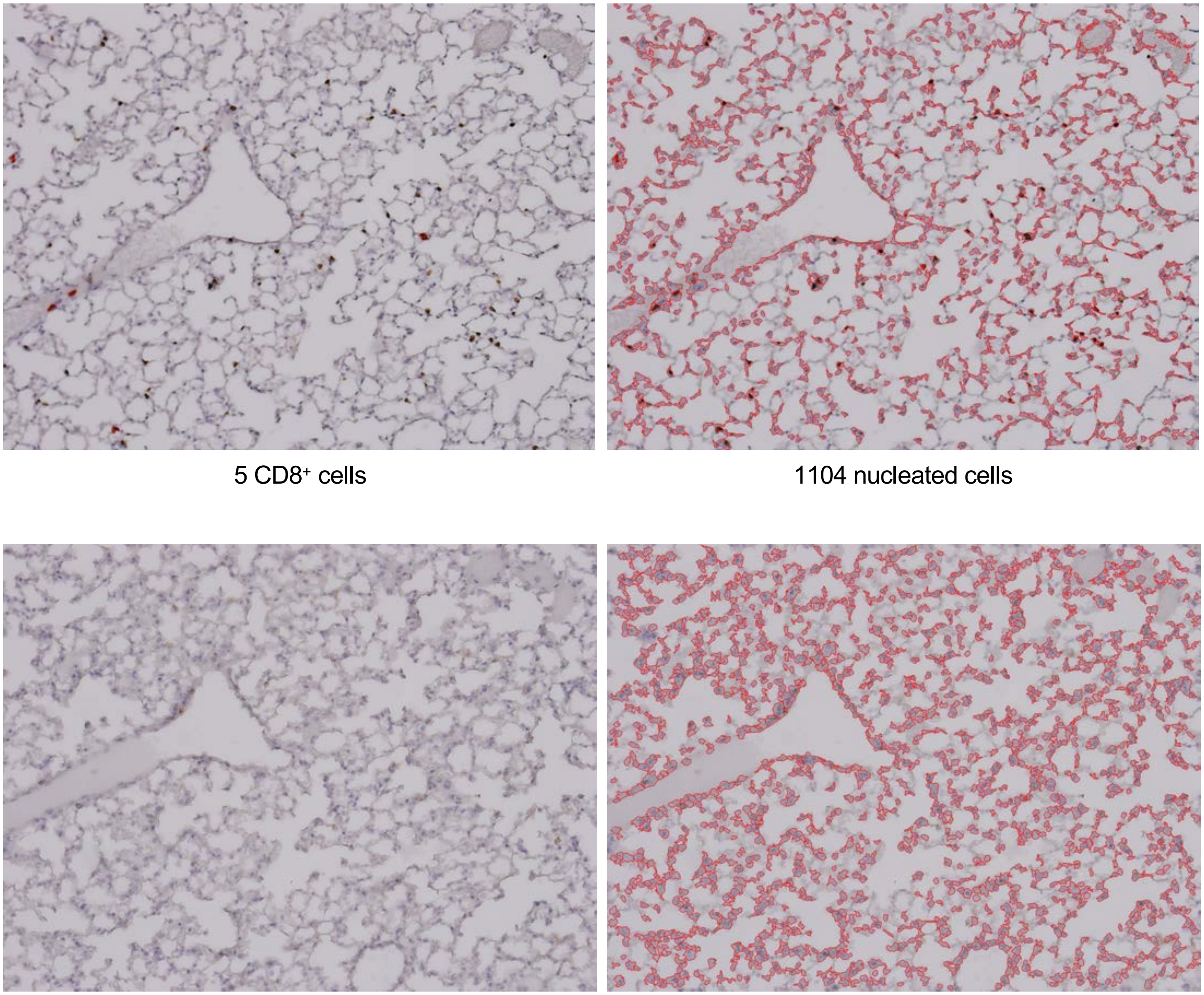

5 CD8$^+$ cells

1104 nucleated cells

0 CD4$^+$ cells

1388 nucleated cells

Supplementary Figure 11
Page 2/30

MIT-T-COVID Lung 1-2
Unchallenged, Female Cohort

CD8$^+$/CD4$^+$ Cell Annotations

Nucleated Cell Annotations

CD8$^+$

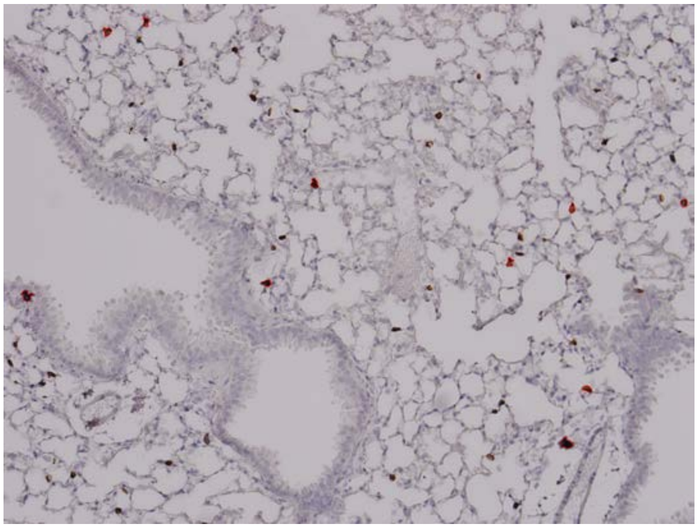

10 CD8$^+$ cells

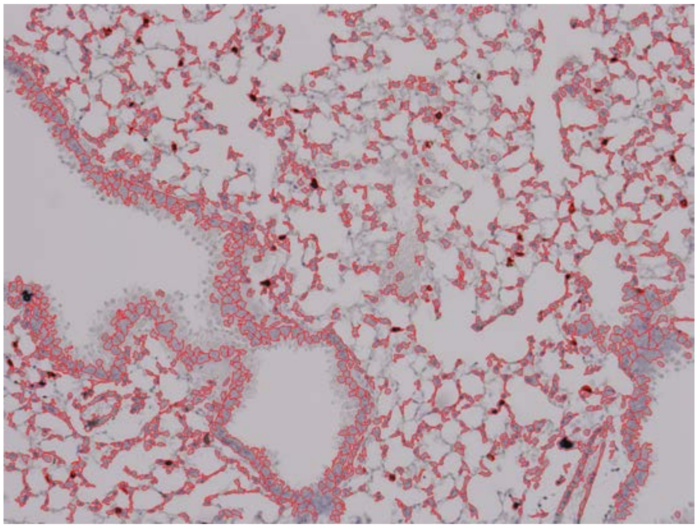

1328 nucleated cells

CD4$^+$

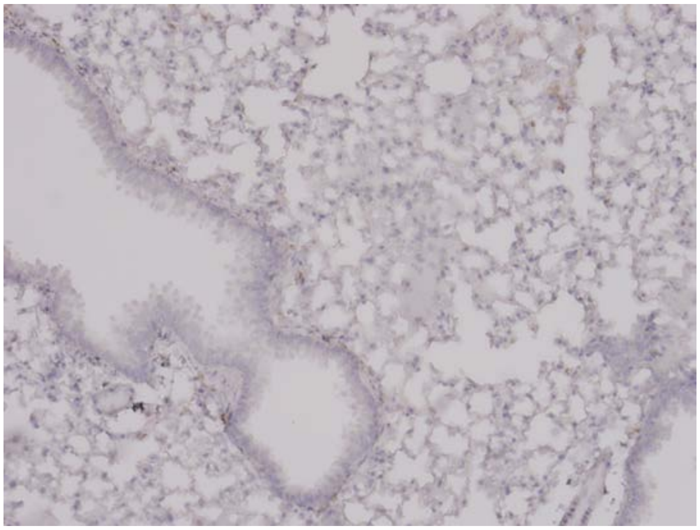

0 CD4$^+$ cells

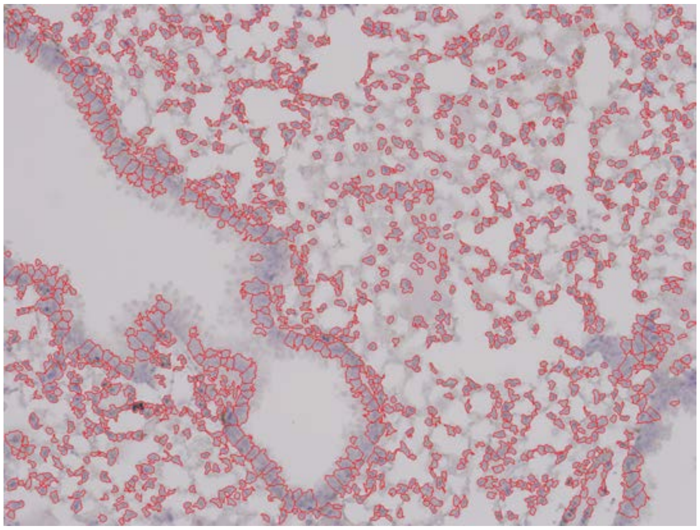

1175 nucleated cells

Supplementary Figure 11

MIT-T-COVID Lung 2-1
Unchallenged, Female Cohort

CD8$^+$/CD4$^+$ Cell Annotations | Nucleated Cell Annotations

CD8$^+$

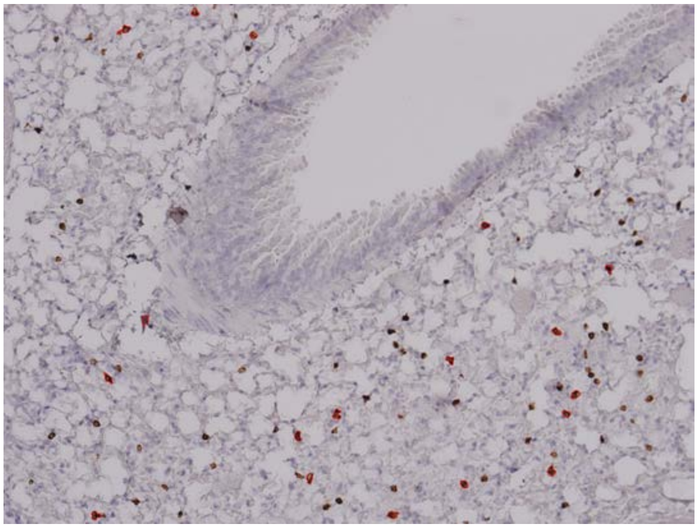

18 CD8$^+$ cells

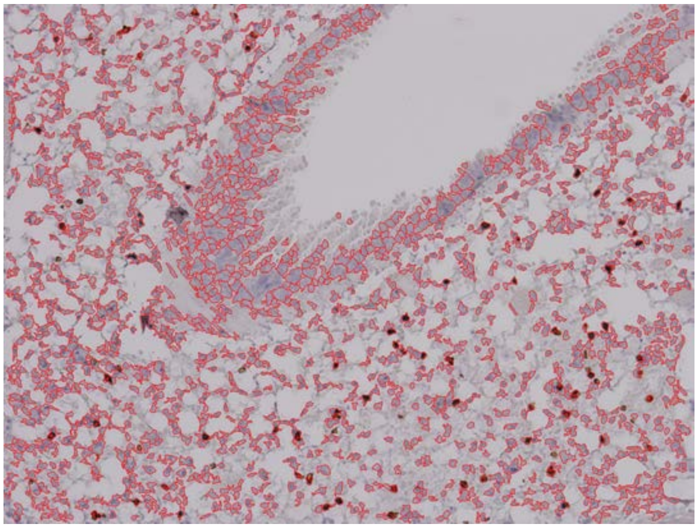

1481 nucleated cells

CD4$^+$

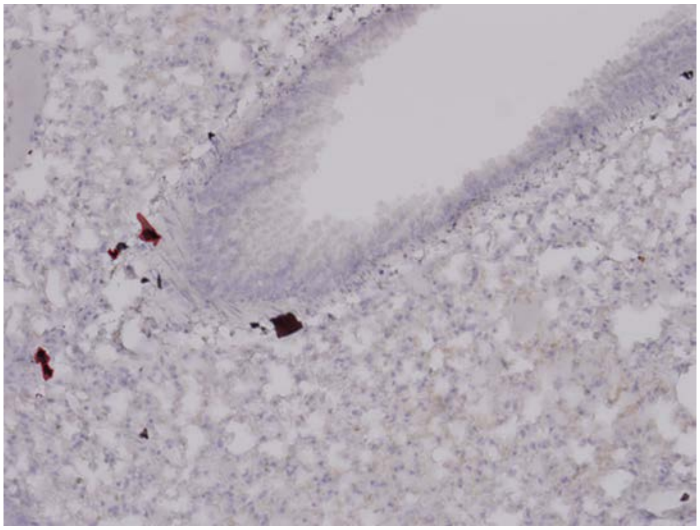

4 CD4$^+$ cells

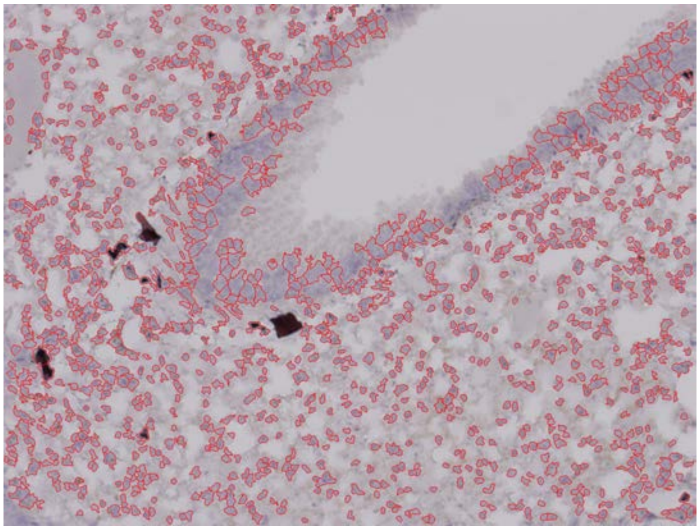

1190 nucleated cells

Supplementary Figure 11
Page 4/30

MIT-T-COVID Lung 2-2
Unchallenged, Female Cohort

CD8$^+$/CD4$^+$ Cell Annotations

Nucleated Cell Annotations

CD8$^+$

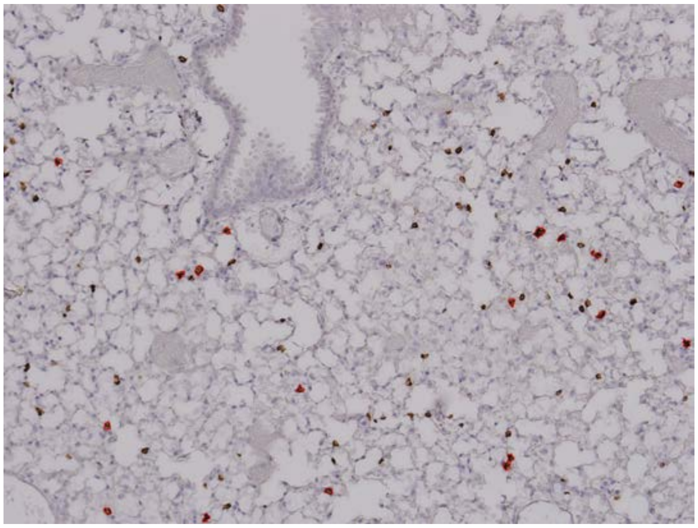

18 CD8$^+$ cells

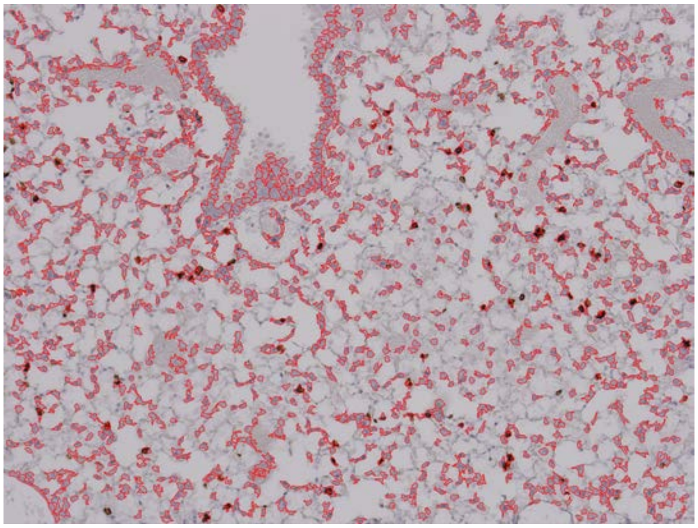

1291 nucleated cells

CD4$^+$

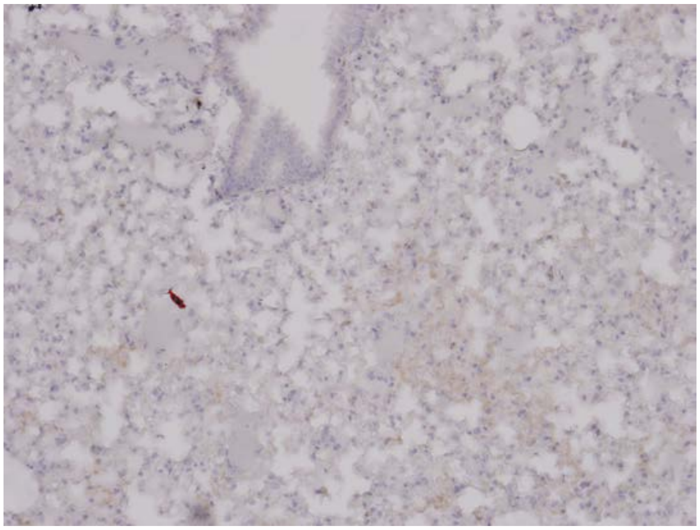

1 CD4$^+$ cells

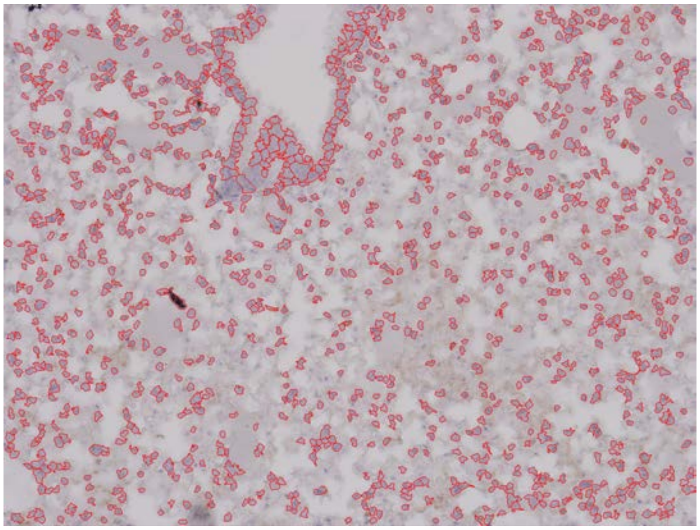

1132 nucleated cells

Supplementary Figure 11
Page 5/30

MIT-T-COVID Lung 2-3
Unchallenged, Female Cohort

	CD8$^+$/CD4$^+$ Cell Annotations	Nucleated Cell Annotations
CD8$^+$	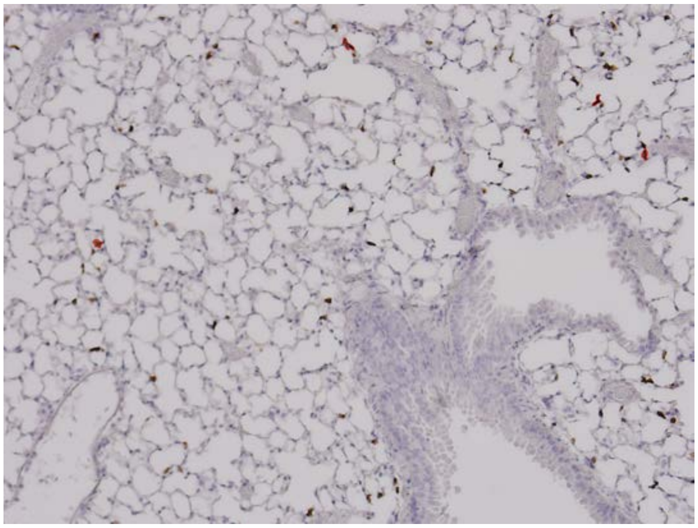	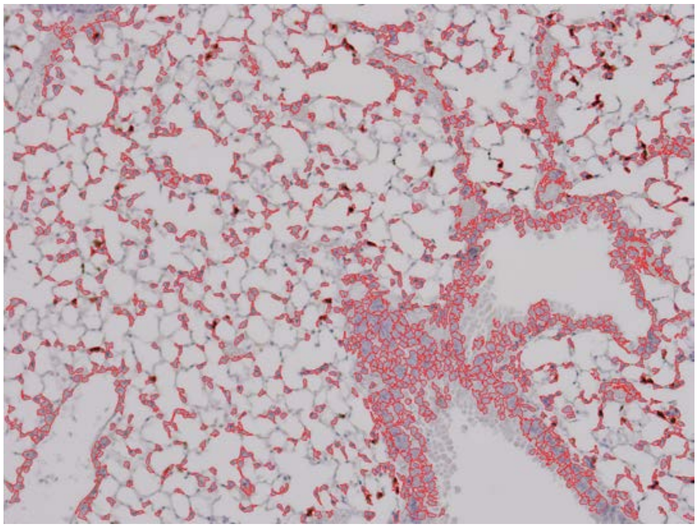
	4 CD8$^+$ cells	1284 nucleated cells
CD4$^+$	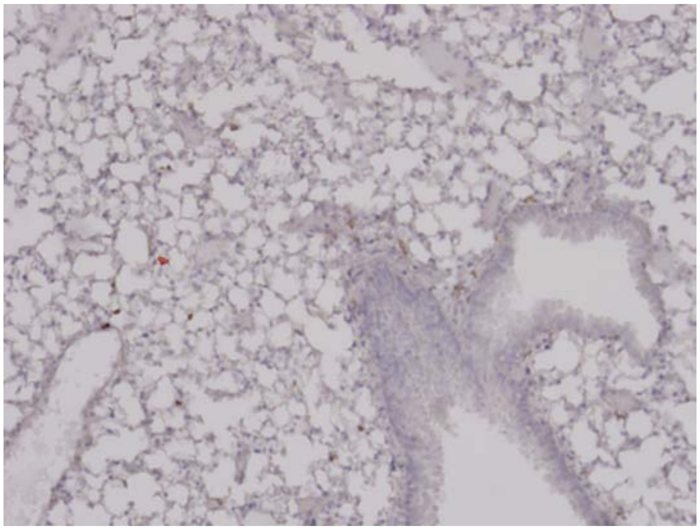	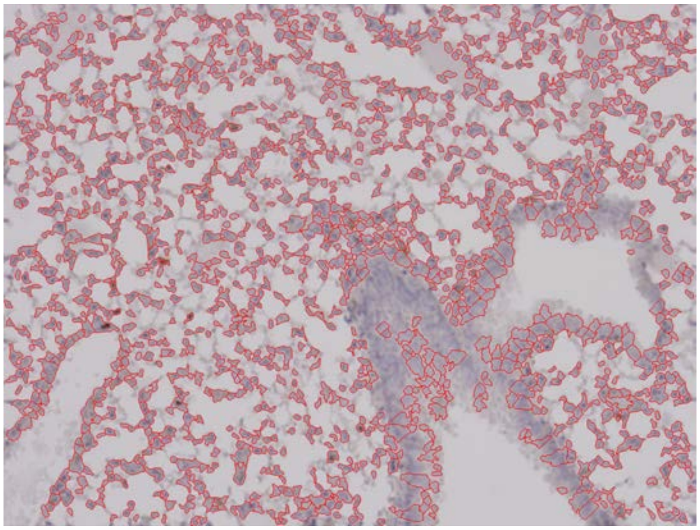
	1 CD4$^+$ cells	1368 nucleated cells

Supplementary Figure 11
Page 6/30

MIT-T-COVID Lung 2-4
Unchallenged, Female Cohort

CD8$^+$/CD4$^+$ Cell Annotations | Nucleated Cell Annotations

CD8$^+$

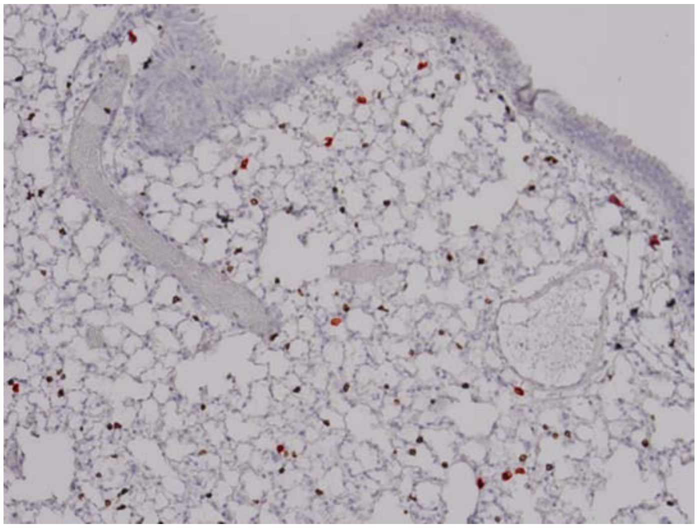

14 CD8$^+$ cells

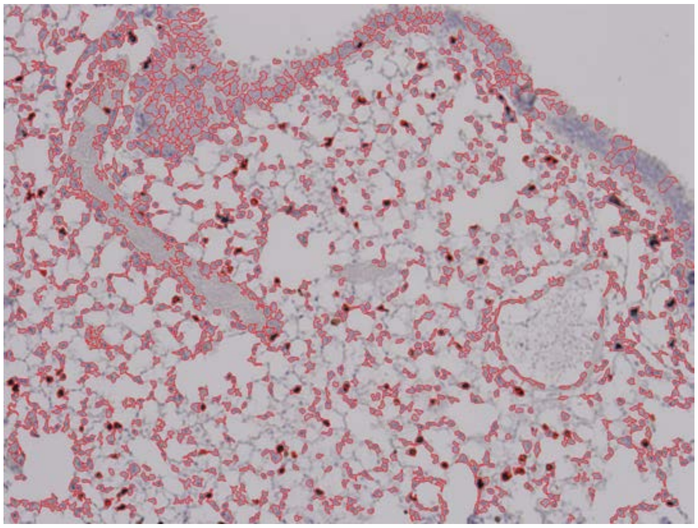

1328 nucleated cells

CD4$^+$

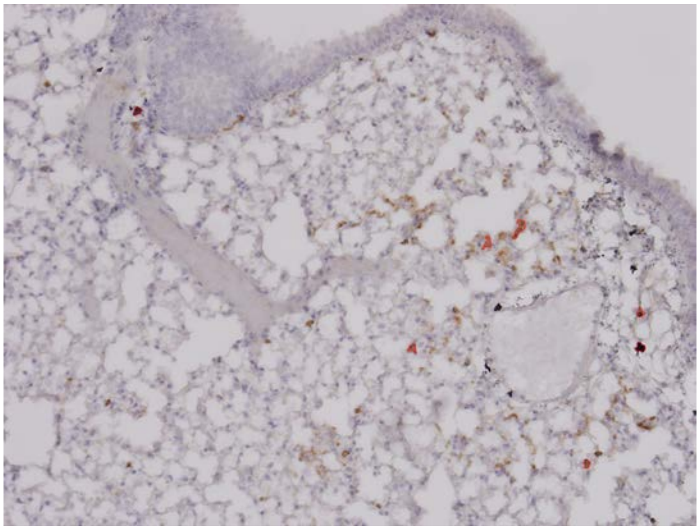

7 CD4$^+$ cells

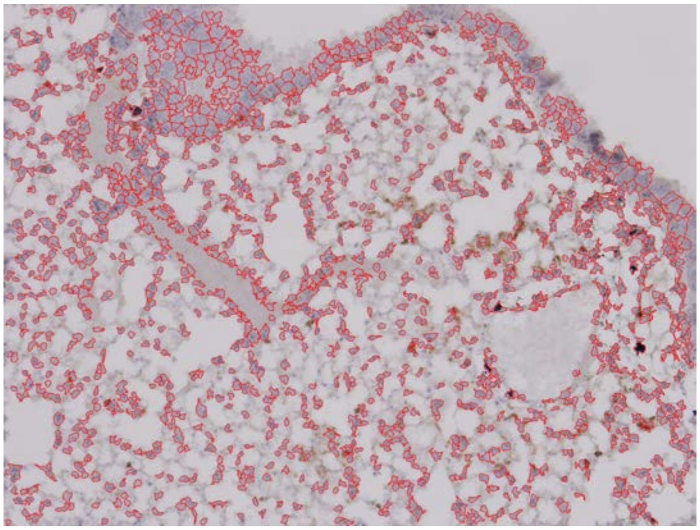

1297 nucleated cells

Supplementary Figure 11
Page 7/30

MIT-T-COVID Lung 3-1
Unchallenged, Female Cohort

CD8$^+$/CD4$^+$ Cell Annotations | Nucleated Cell Annotations

CD8$^+$

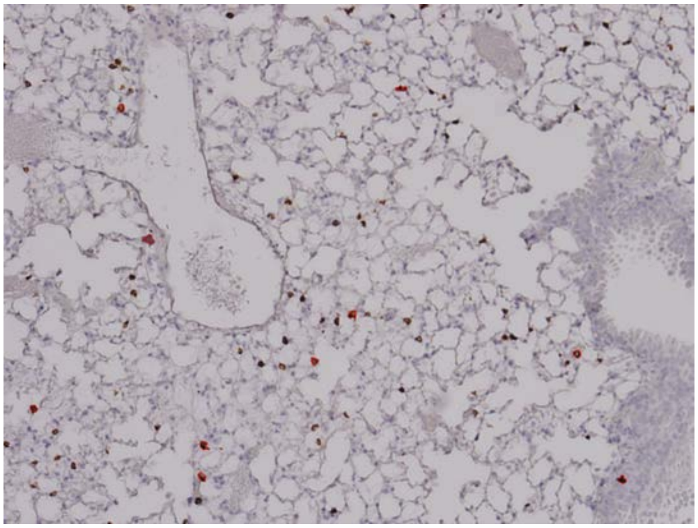

10 CD8$^+$ cells

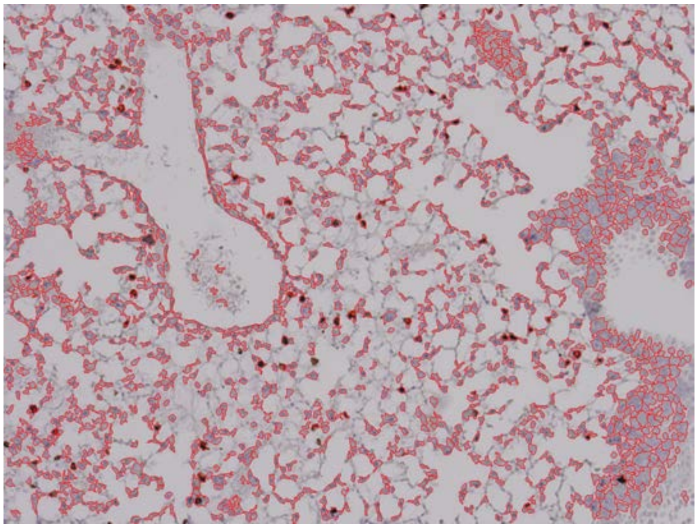

1458 nucleated cells

CD4$^+$

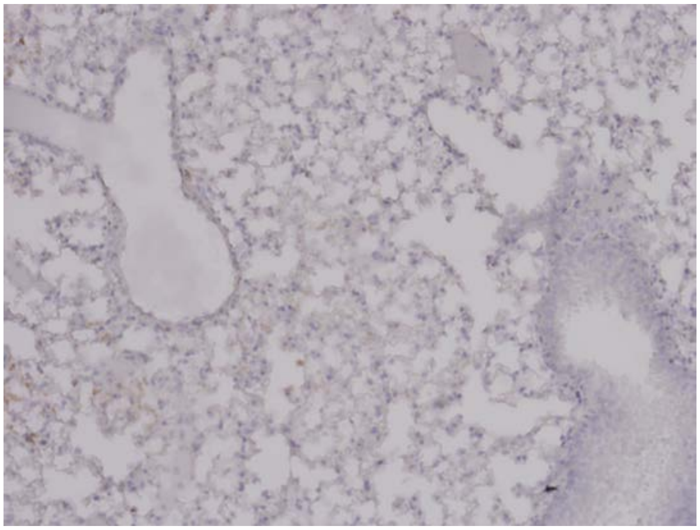

0 CD4$^+$ cells

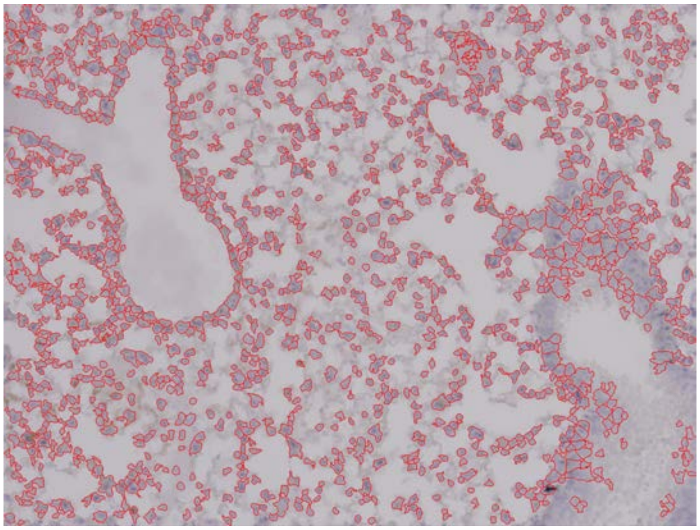

1272 nucleated cells

Supplementary Figure 11
Page 8/30

MIT-T-COVID Lung 3-2
Unchallenged, Female Cohort

CD8^{+}/CD4^{+} Cell Annotations | Nucleated Cell Annotations

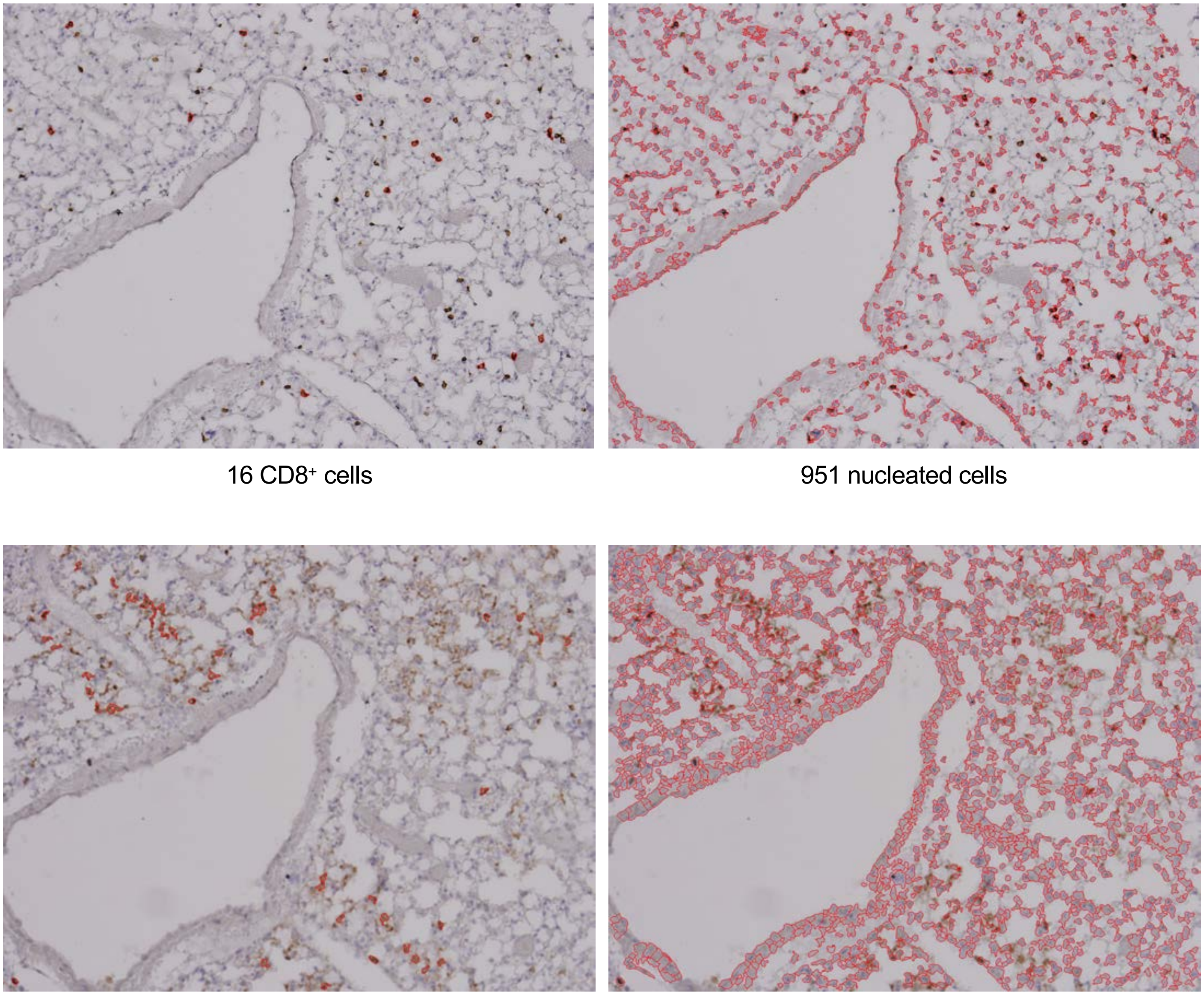

CD8^{+}: 16 CD8^{+} cells; 951 nucleated cells

CD4^{+}: 46 CD4^{+} cells; 1484 nucleated cells

Supplementary Figure 11

MIT-T-COVID Lung 3-3
Unchallenged, Female Cohort

CD8^{+}/CD4^{+} Cell Annotations | Nucleated Cell Annotations

CD8^{+}

CD4^{+}

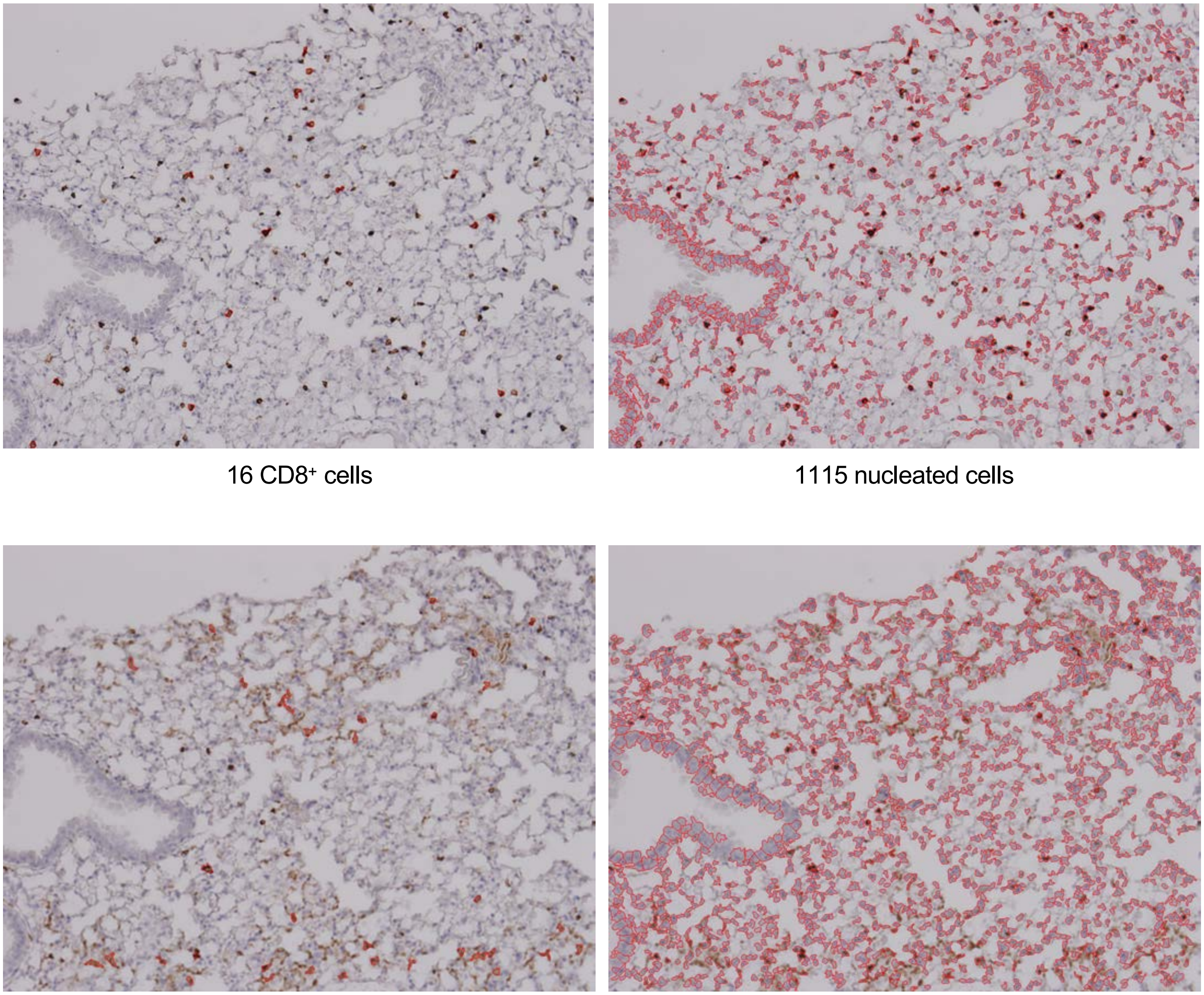

16 CD8^{+} cells | 1115 nucleated cells

37 CD4^{+} cells | 1499 nucleated cells

Supplementary Figure 11
Page 10/30

MIT-T-COVID Lung 3-4
Unchallenged, Female Cohort

CD8$^+$/CD4$^+$ Cell Annotations | Nucleated Cell Annotations

CD8$^+$

CD4$^+$

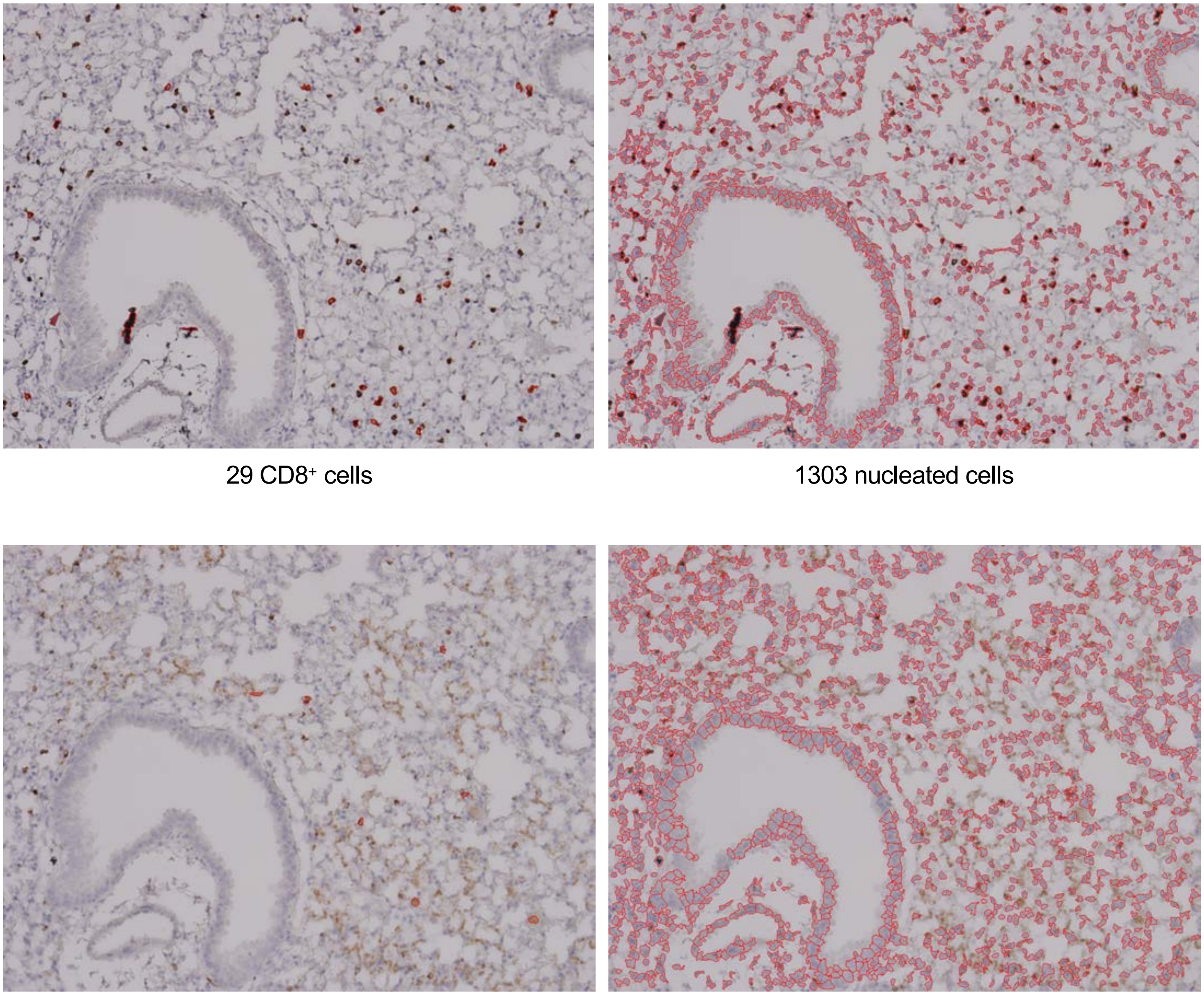

29 CD8$^+$ cells | 1303 nucleated cells

7 CD4$^+$ cells | 1412 nucleated cells

Pfizer/BNT Lung 1-1
Unchallenged, Female Cohort

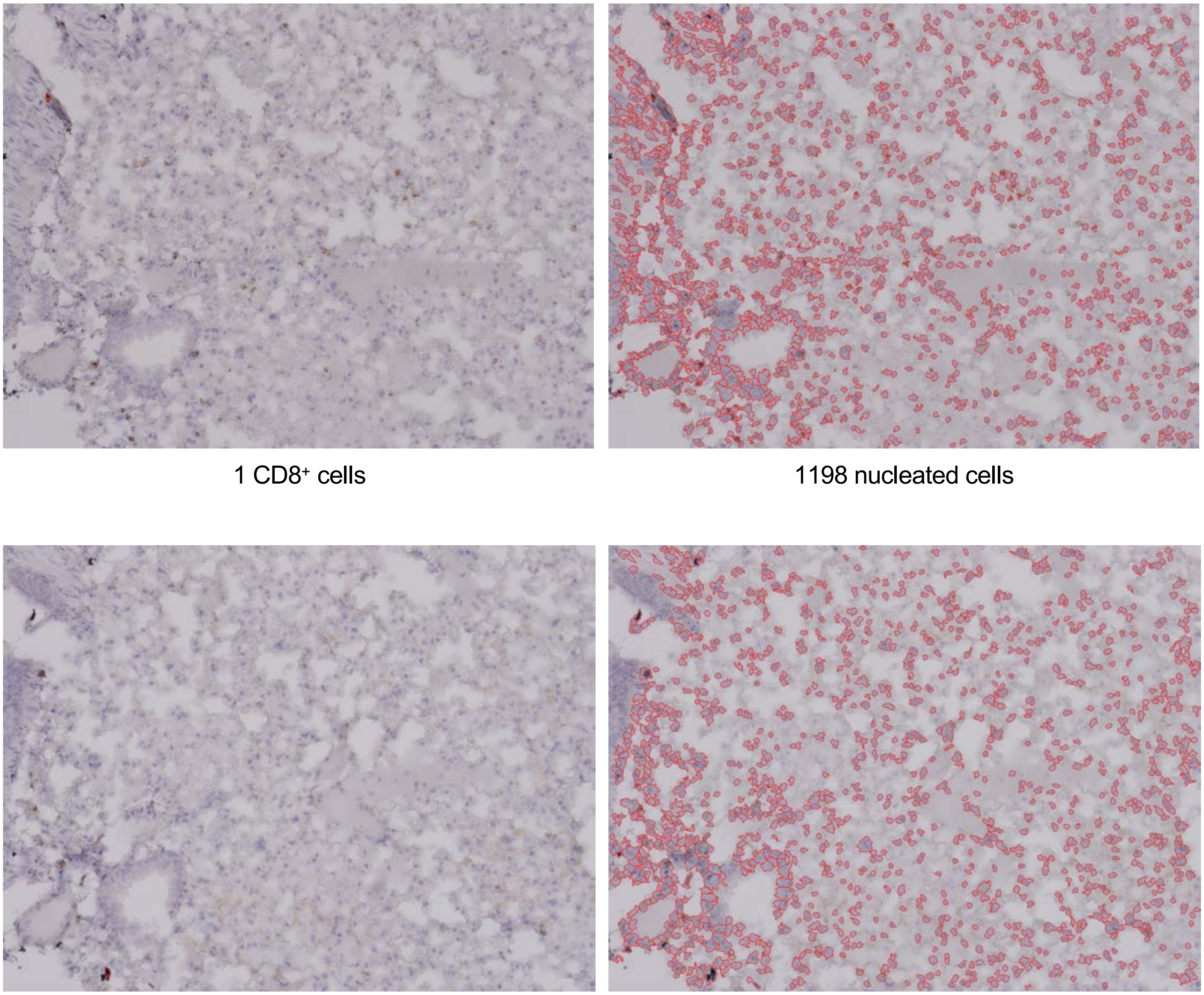

Pfizer/BNT Lung 1-2
Unchallenged, Female Cohort

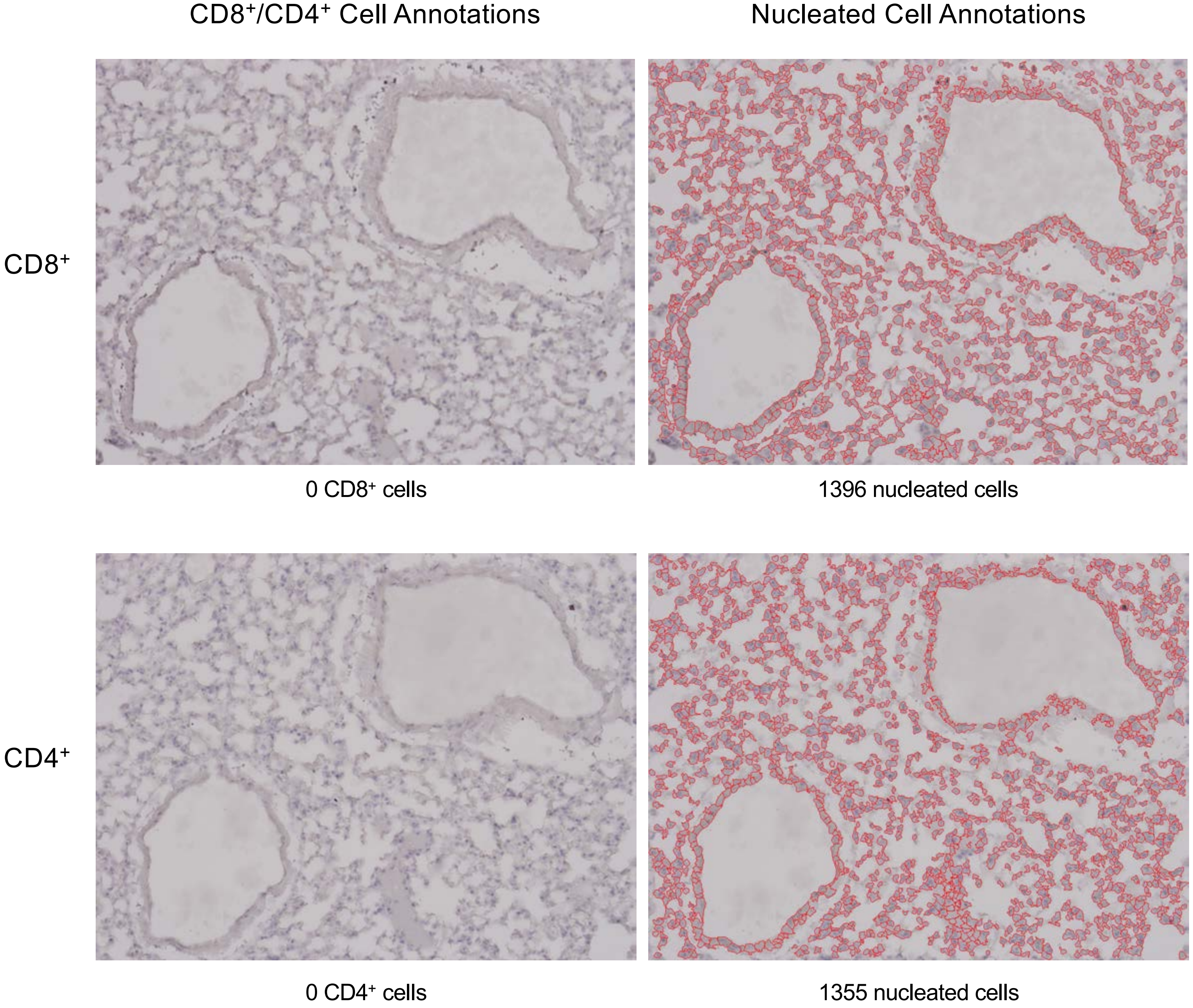

Pfizer/BNT Lung 2-1
Unchallenged, Female Cohort

CD8$^+$/CD4$^+$ Cell Annotations | Nucleated Cell Annotations

CD8$^+$

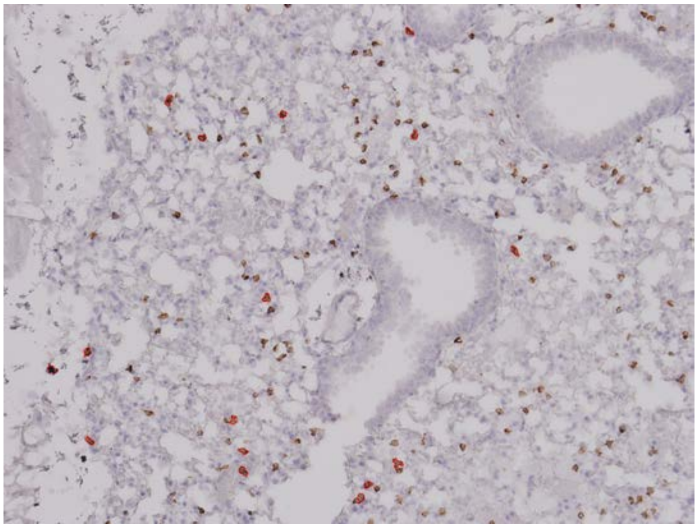

16 CD8$^+$ cells

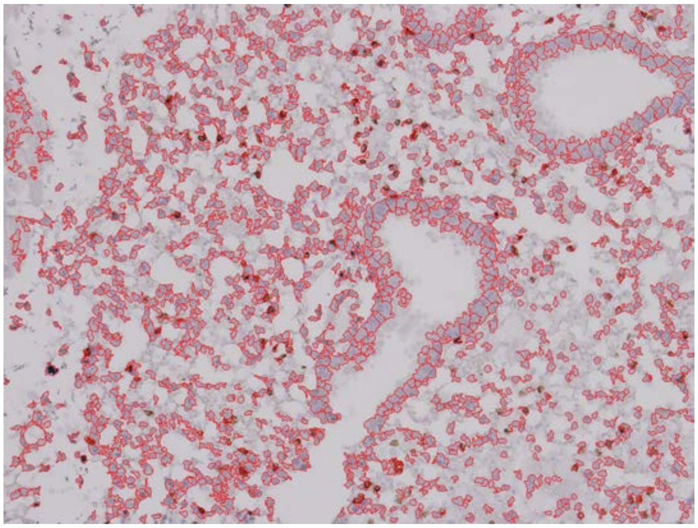

1544 nucleated cells

CD4$^+$

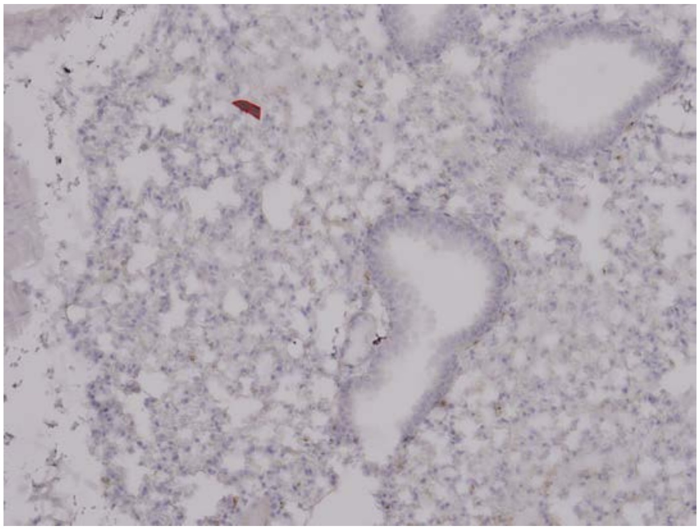

2 CD4$^+$ cells

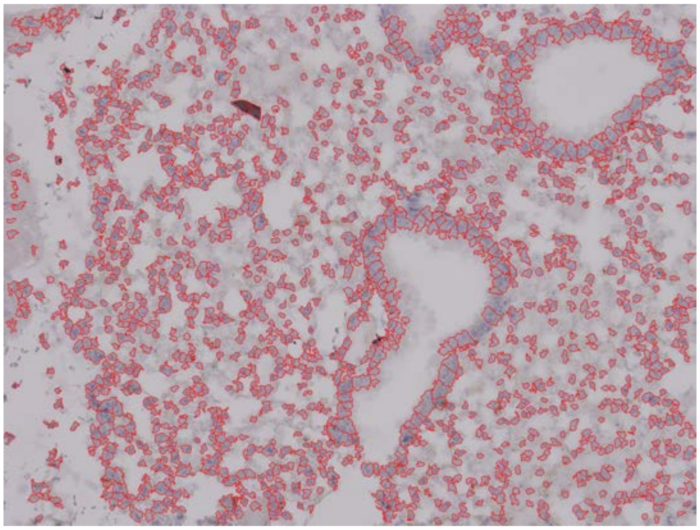

1447 nucleated cells

Supplementary Figure 11

Pfizer/BNT Lung 2-2
Unchallenged, Female Cohort

CD8$^+$/CD4$^+$ Cell Annotations

Nucleated Cell Annotations

CD8$^+$

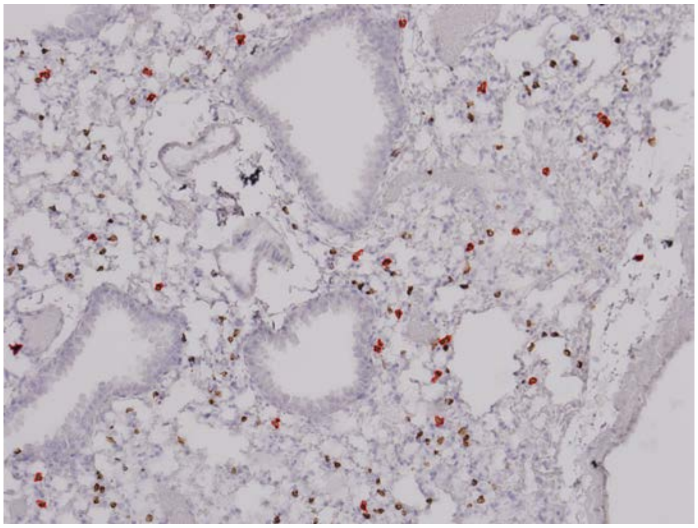

26 CD8$^+$ cells

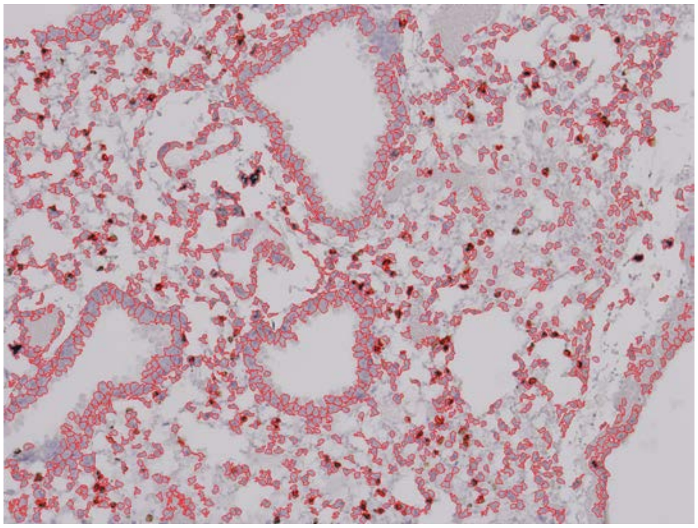

1475 nucleated cells

CD4$^+$

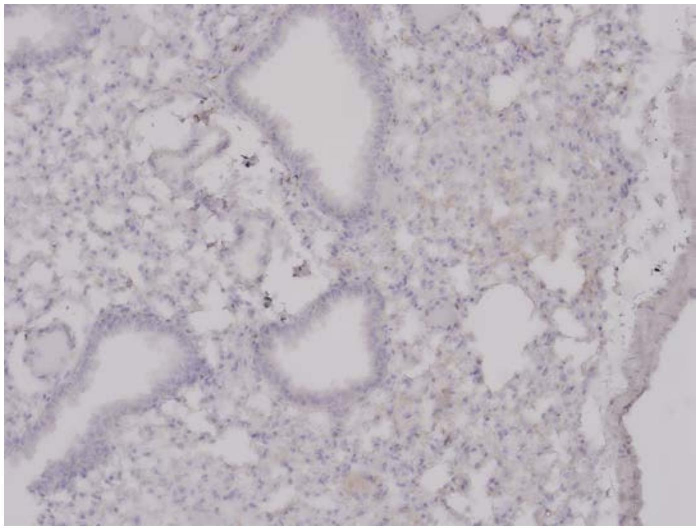

0 CD4$^+$ cells

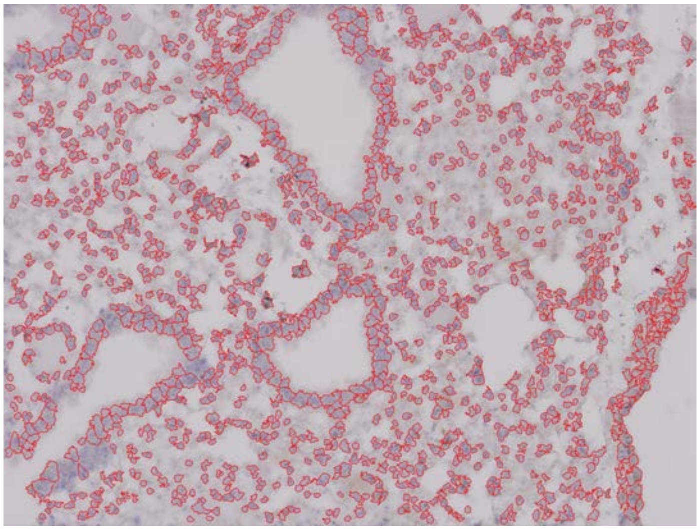

1376 nucleated cells

Supplementary Figure 11

Pfizer/BNT Lung 2-3
Unchallenged, Female Cohort

CD8$^+$/CD4$^+$ Cell Annotations

Nucleated Cell Annotations

CD8$^+$

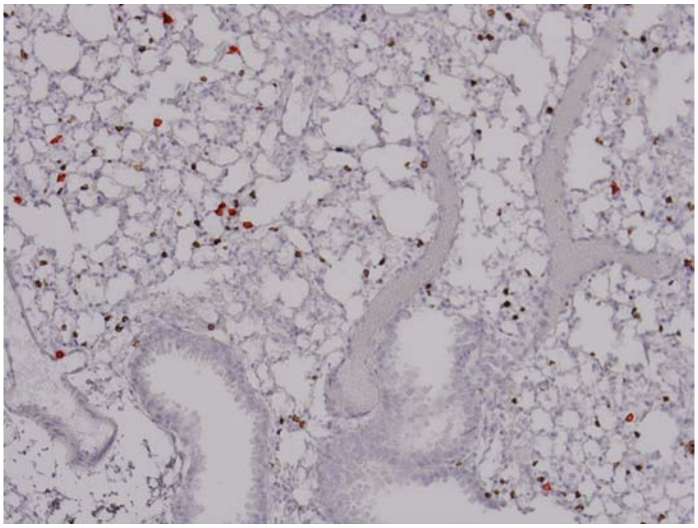

13 CD8$^+$ cells

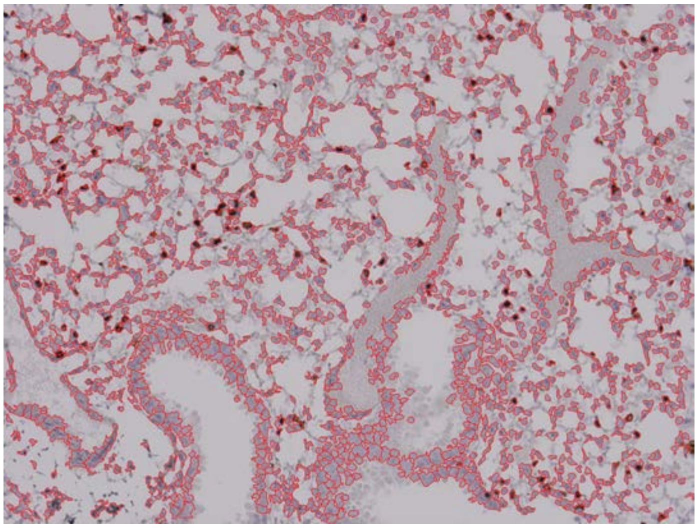

1582 nucleated cells

CD4$^+$

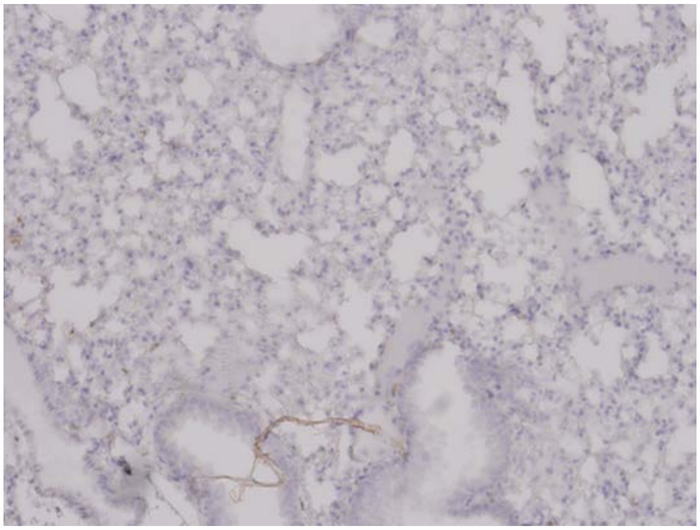

0 CD4$^+$ cells

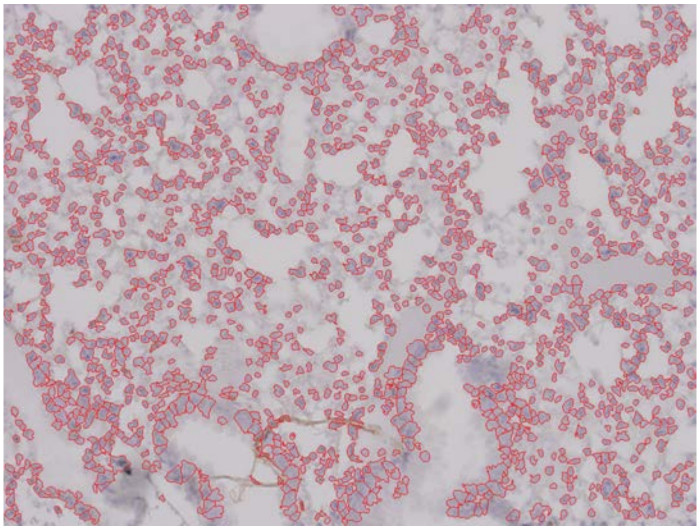

1440 nucleated cells

Supplementary Figure 11
Page 16/30

Pfizer/BNT Lung 2-4
Unchallenged, Female Cohort

CD8$^+$/CD4$^+$ Cell Annotations

Nucleated Cell Annotations

CD8$^+$

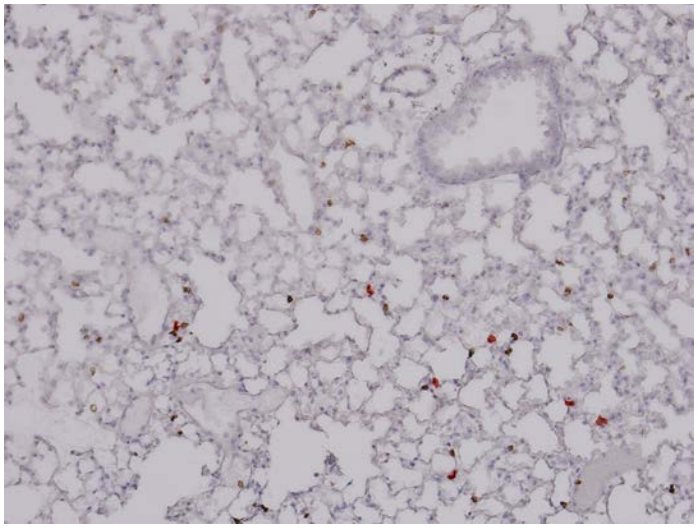

7 CD8$^+$ cells

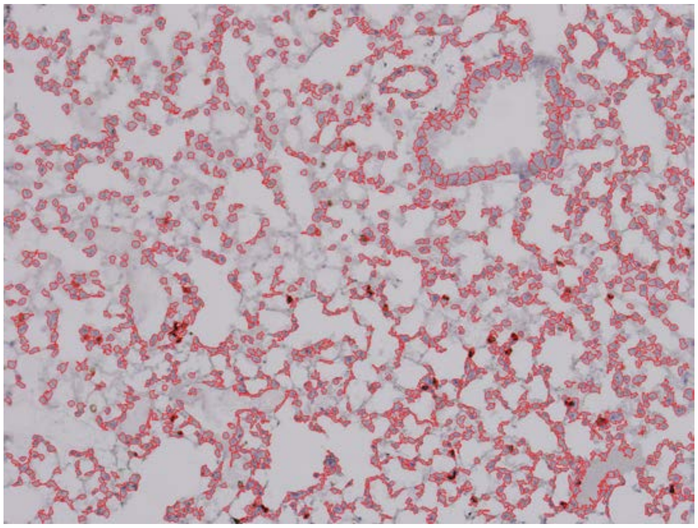

1389 nucleated cells

CD4$^+$

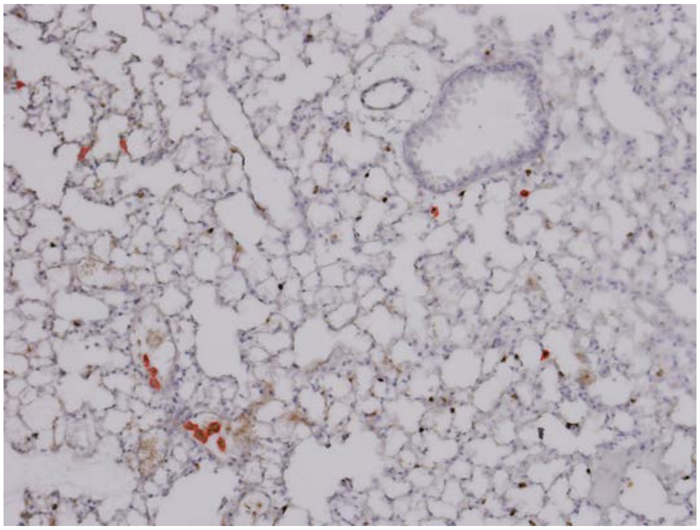

13 CD4$^+$ cells

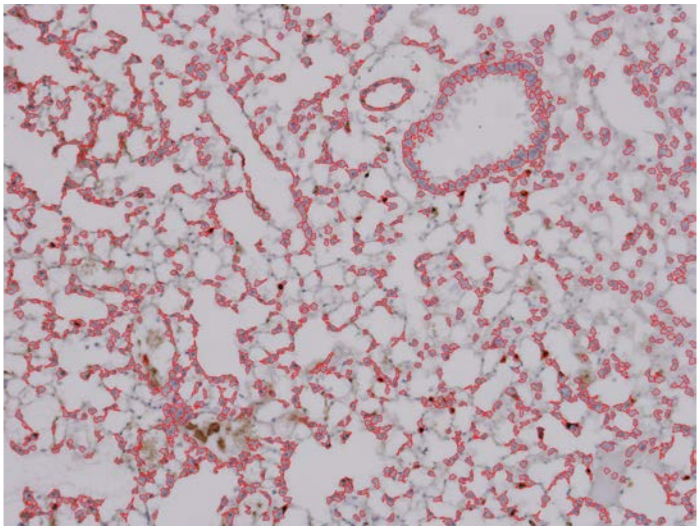

1197 nucleated cells

Supplementary Figure 11
Page 17/30

Pfizer/BNT Lung 3-1
Unchallenged, Female Cohort

CD8$^+$/CD4$^+$ Cell Annotations | Nucleated Cell Annotations

CD8$^+$

CD4$^+$

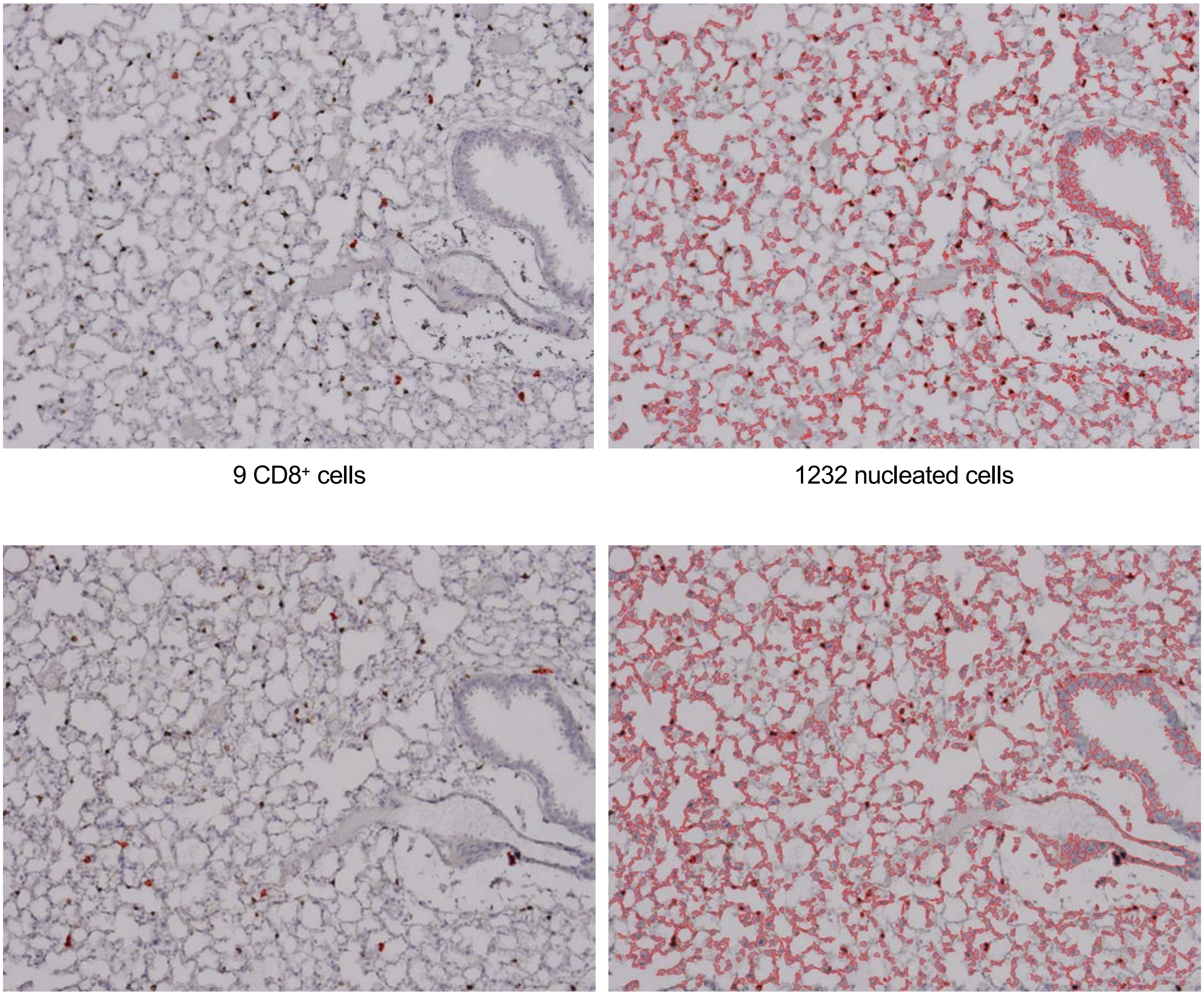

9 CD8$^+$ cells

1232 nucleated cells

9 CD4$^+$ cells

1521 nucleated cells

Supplementary Figure 11
Page 18/30

Pfizer/BNT Lung 3-2
Unchallenged, Female Cohort

CD8$^+$/CD4$^+$ Cell Annotations

Nucleated Cell Annotations

CD8$^+$

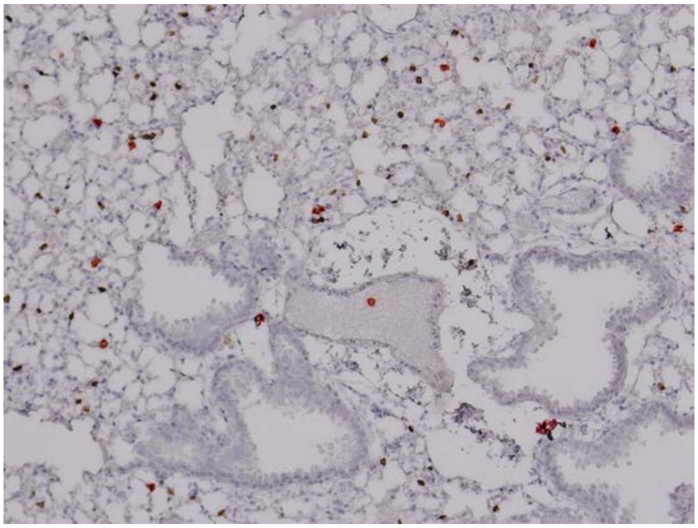

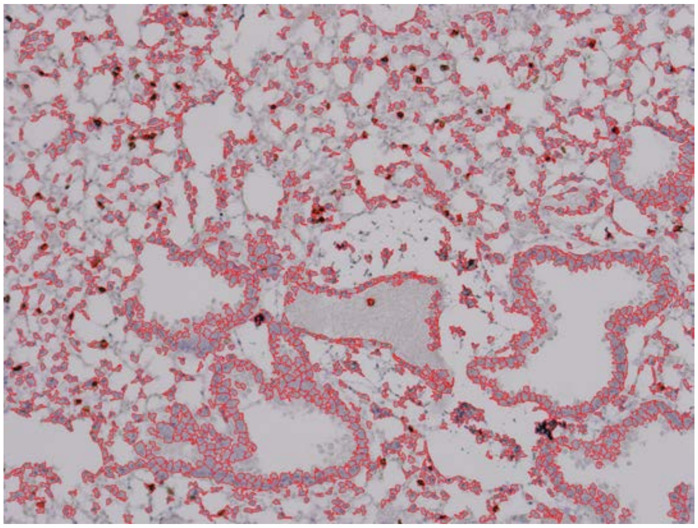

16 CD8$^+$ cells

1499 nucleated cells

CD4$^+$

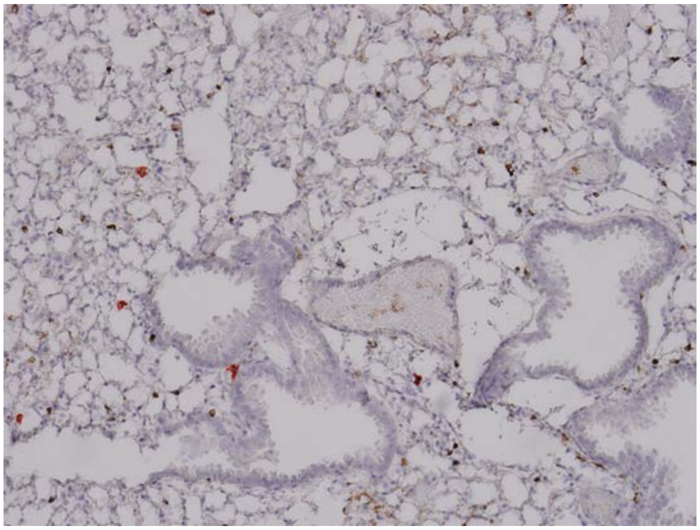

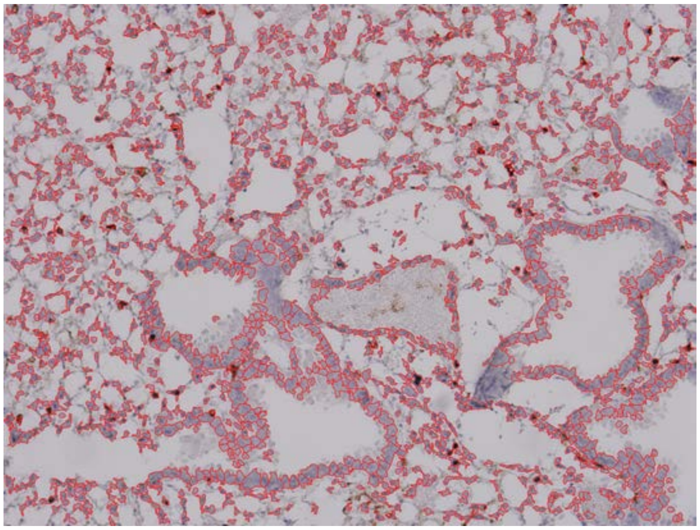

5 CD4$^+$ cells

1464 nucleated cells

Pfizer/BNT Lung 3-3
Unchallenged, Female Cohort

CD8$^+$/CD4$^+$ Cell Annotations | Nucleated Cell Annotations

CD8$^+$

CD4$^+$

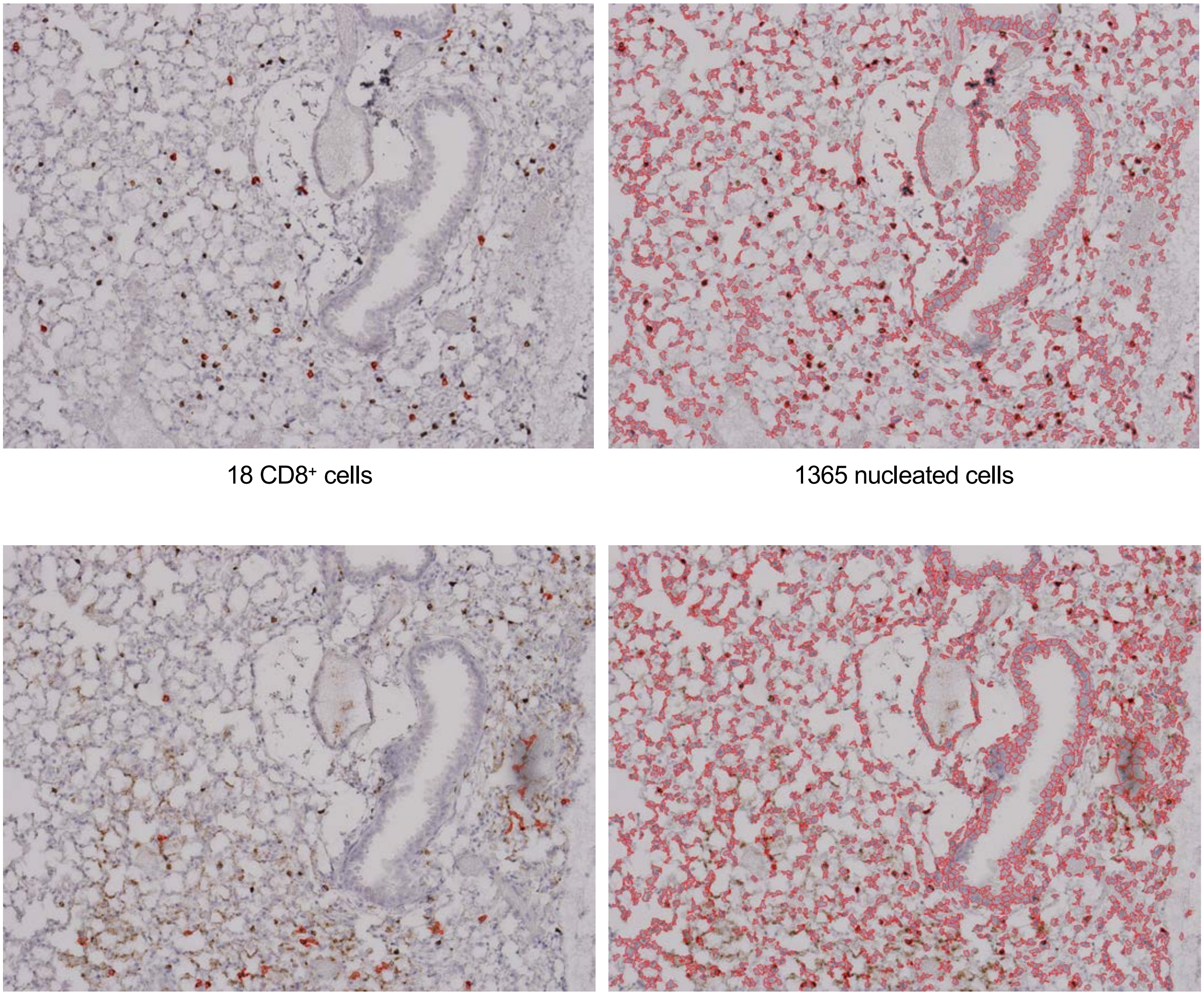

18 CD8$^+$ cells

1365 nucleated cells

28 CD4$^+$ cells

1541 nucleated cells

Pfizer/BNT Lung 3-4
Unchallenged, Female Cohort

CD8$^+$/CD4$^+$ Cell Annotations | Nucleated Cell Annotations

CD8$^+$

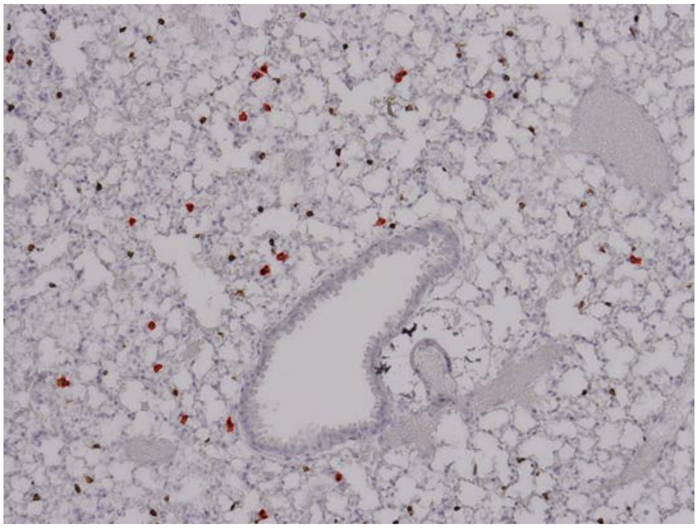

20 CD8$^+$ cells

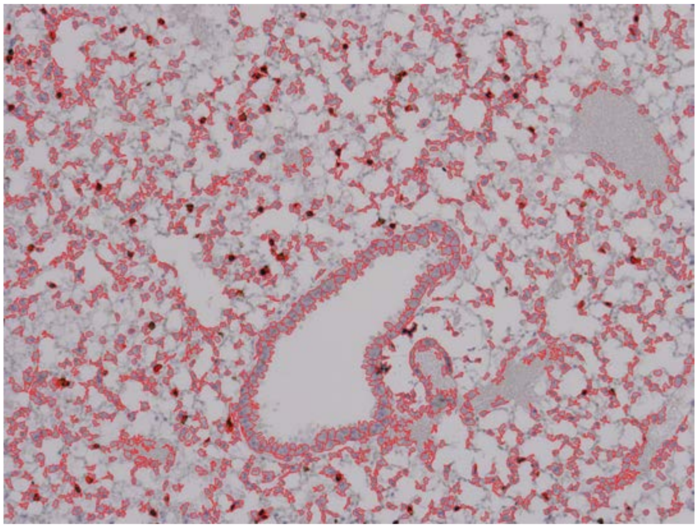

1464 nucleated cells

CD4$^+$

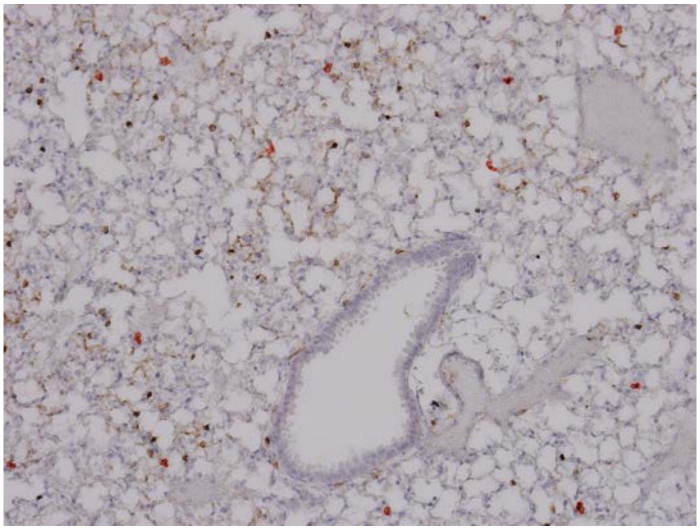

13 CD4$^+$ cells

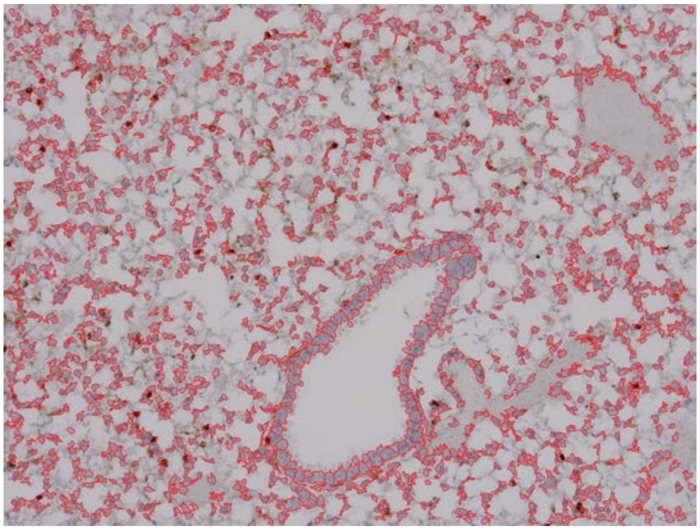

1400 nucleated cells

Supplementary Figure 11

PBS Lung 1-1
Unchallenged, Female Cohort

CD8^{+}/CD4^{+} Cell Annotations | Nucleated Cell Annotations

CD8^{+}

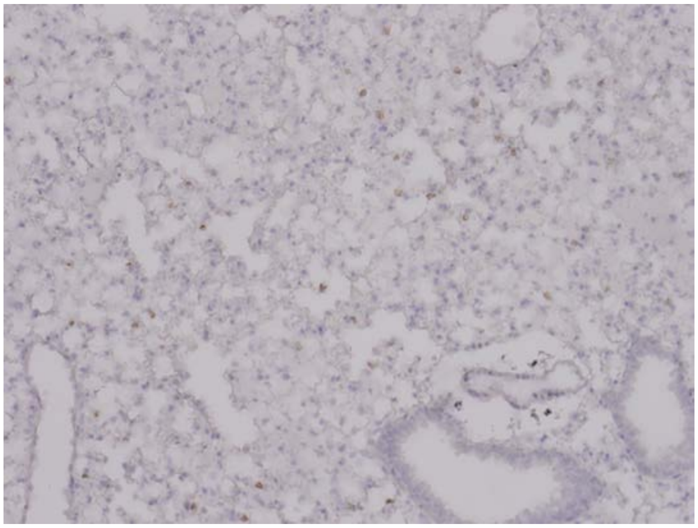

0 CD8^{+} cells

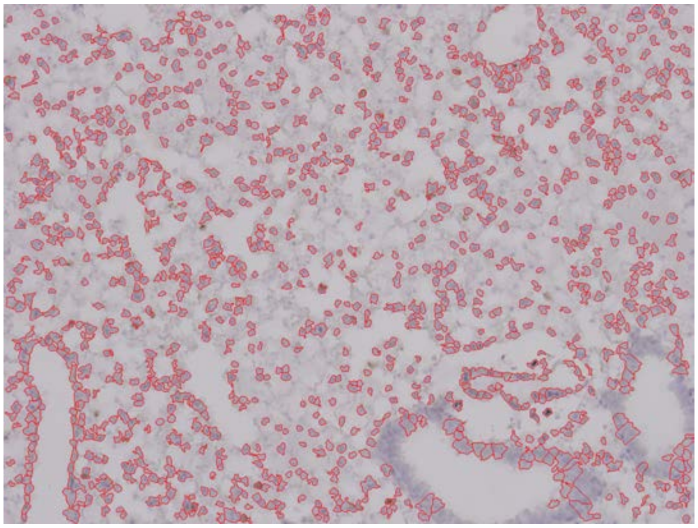

1136 nucleated cells

CD4^{+}

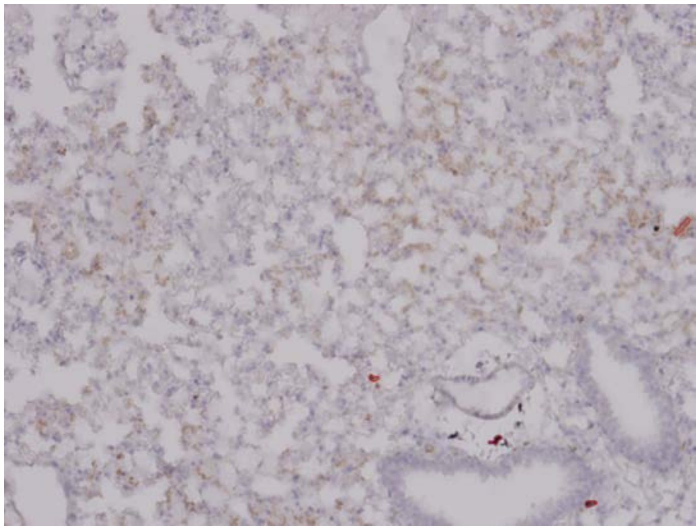

4 CD4^{+} cells

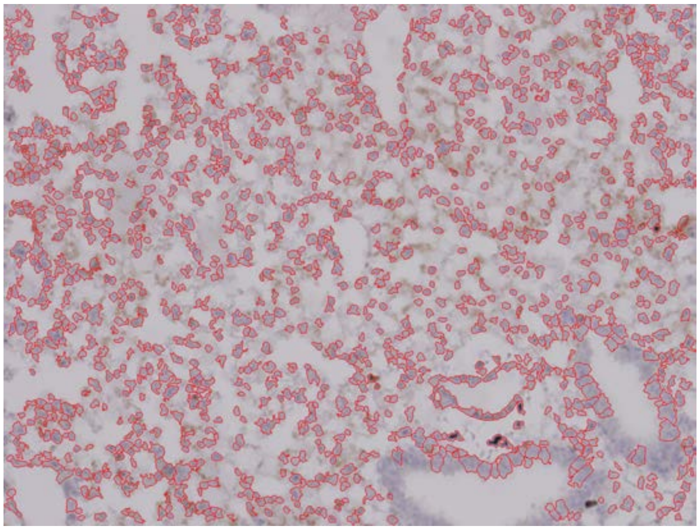

1299 nucleated cells

Supplementary Figure 11
Page 22/30

PBS Lung 1-2
Unchallenged, Female Cohort

CD8$^+$/CD4$^+$ Cell Annotations | Nucleated Cell Annotations

CD8$^+$

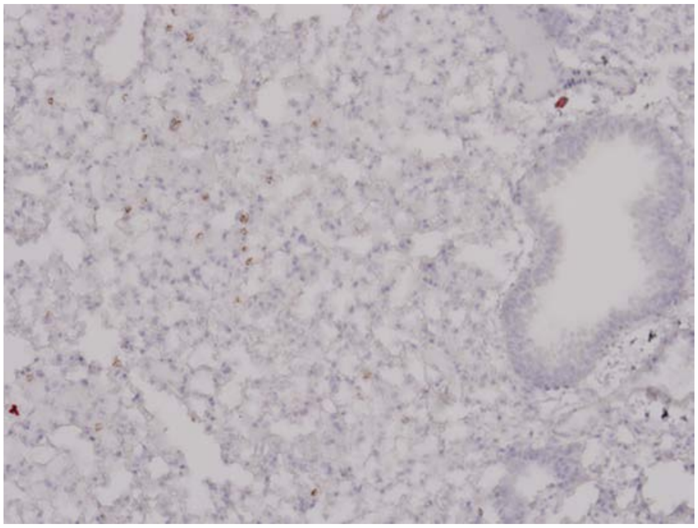

2 CD8$^+$ cells

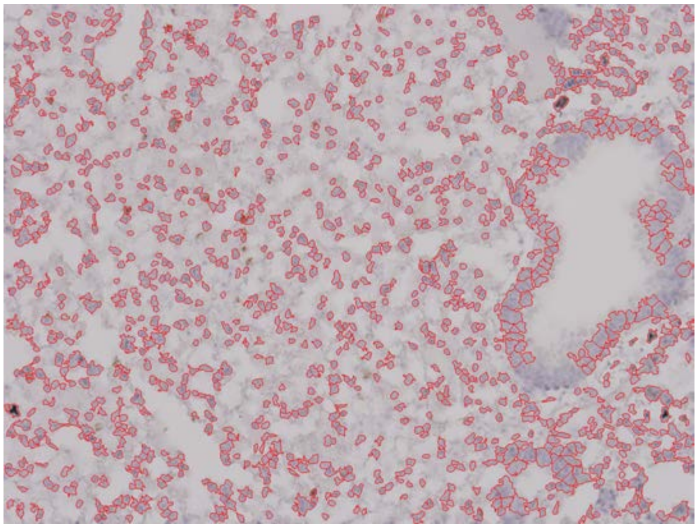

1190 nucleated cells

CD4$^+$

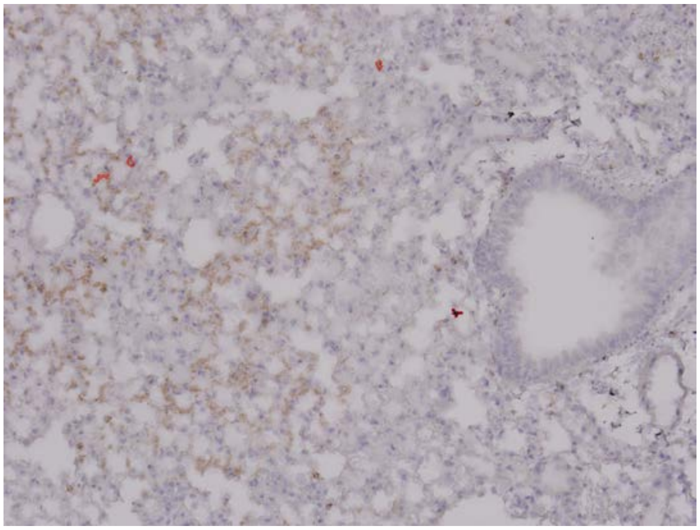

4 CD4$^+$ cells

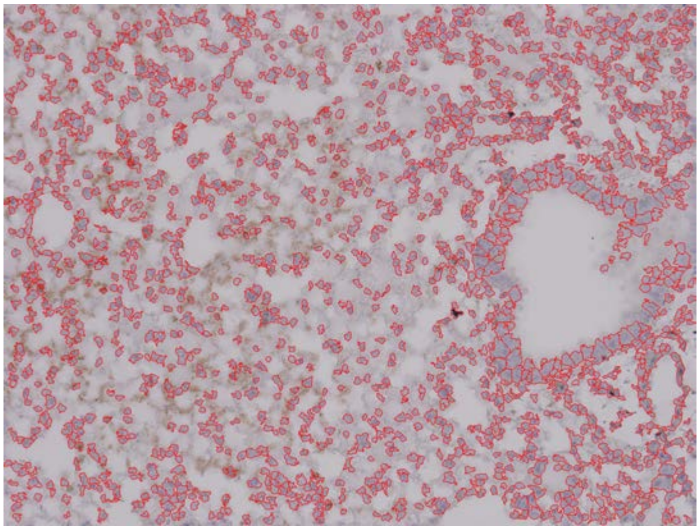

1497 nucleated cells

PBS Lung 2-1
Unchallenged, Female Cohort

CD8$^+$/CD4$^+$ Cell Annotations | Nucleated Cell Annotations

CD8$^+$

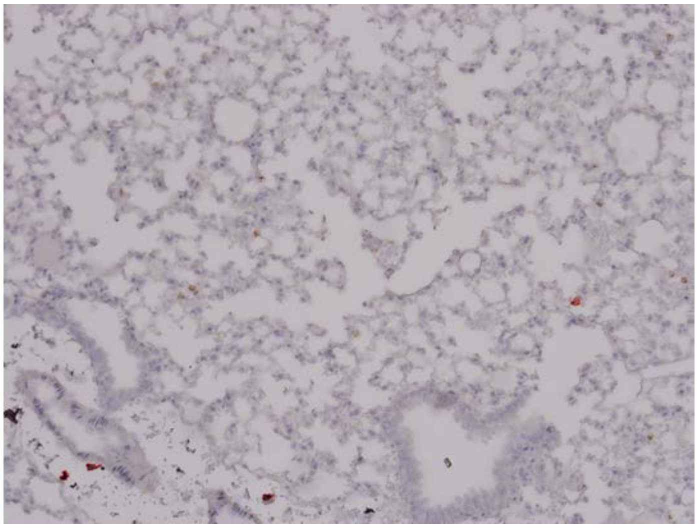

4 CD8$^+$ cells

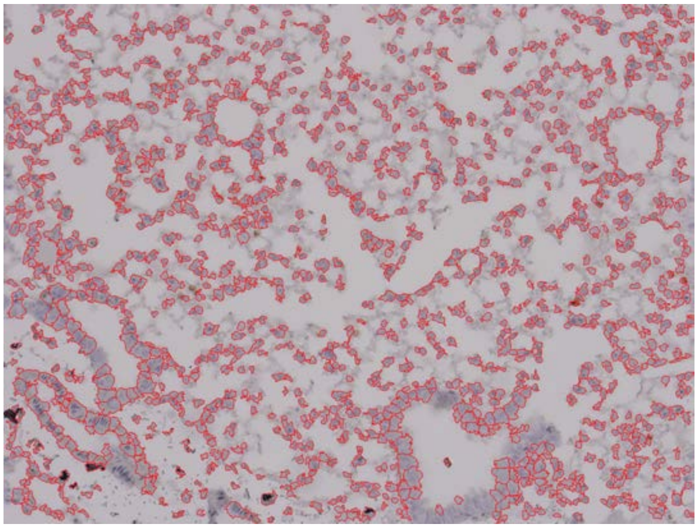

1307 nucleated cells

CD4$^+$

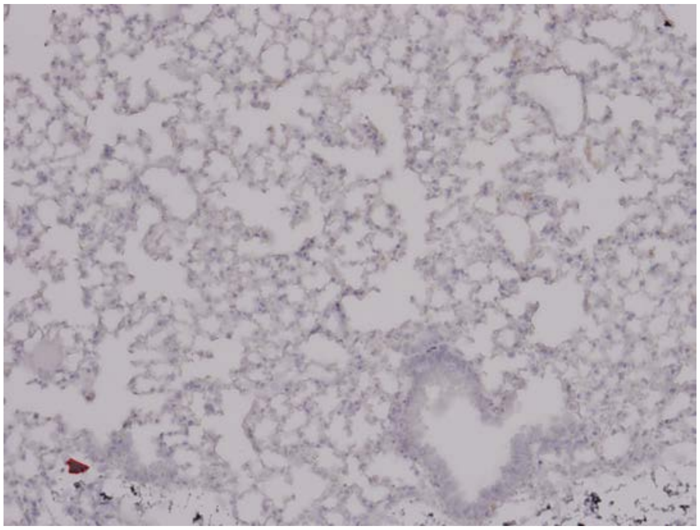

1 CD4$^+$ cells

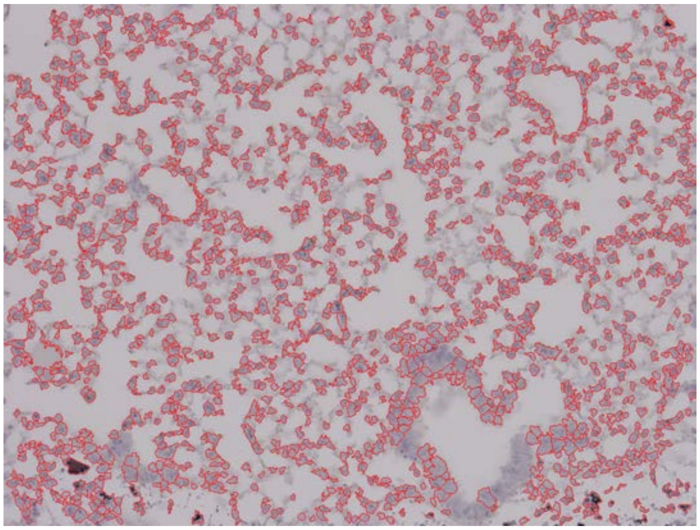

1452 nucleated cells

PBS Lung 2-2
Unchallenged, Female Cohort

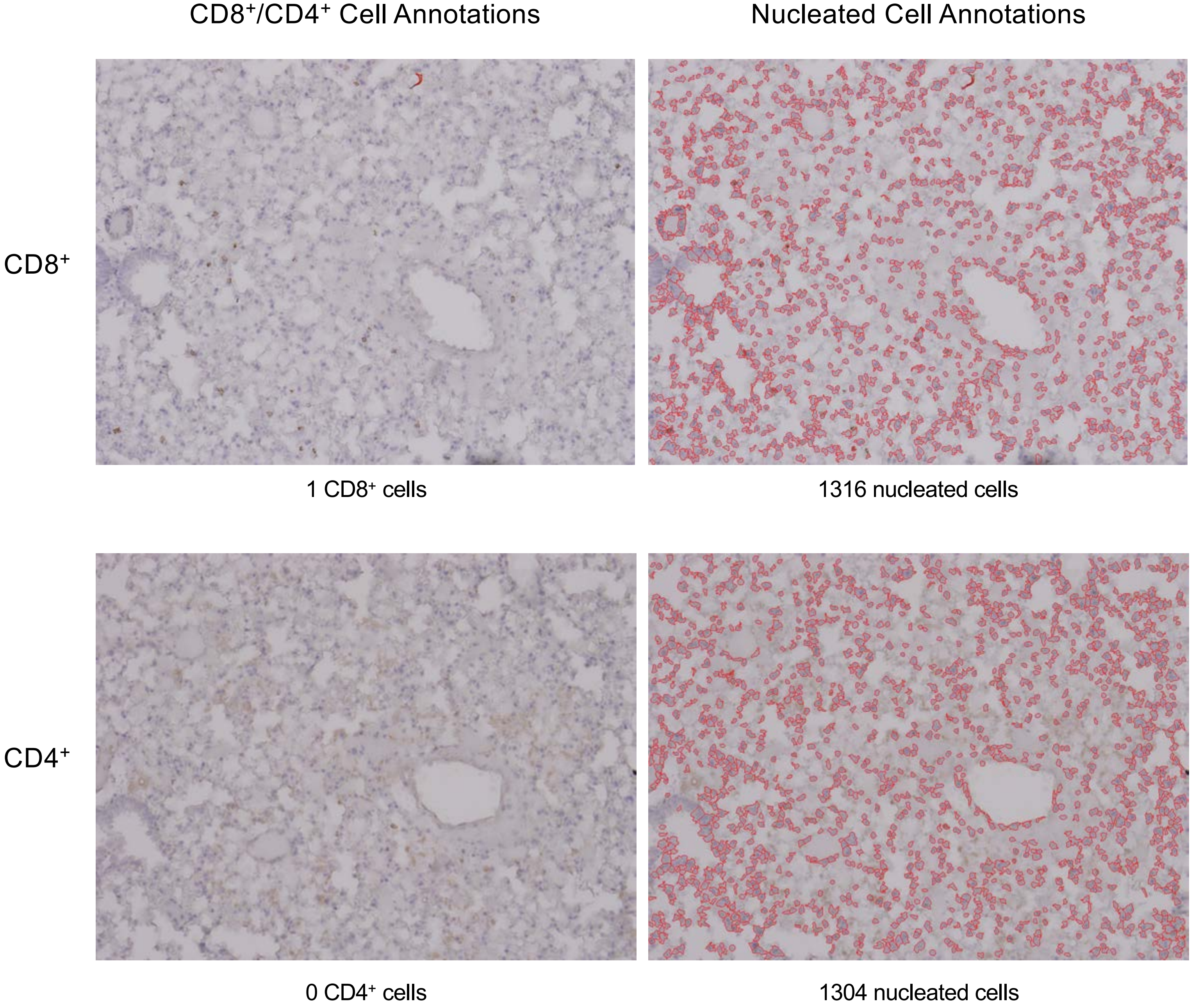

Supplementary Figure 11

PBS Lung 2-3
Unchallenged, Female Cohort

CD8$^+$/CD4$^+$ Cell Annotations | Nucleated Cell Annotations

CD8$^+$

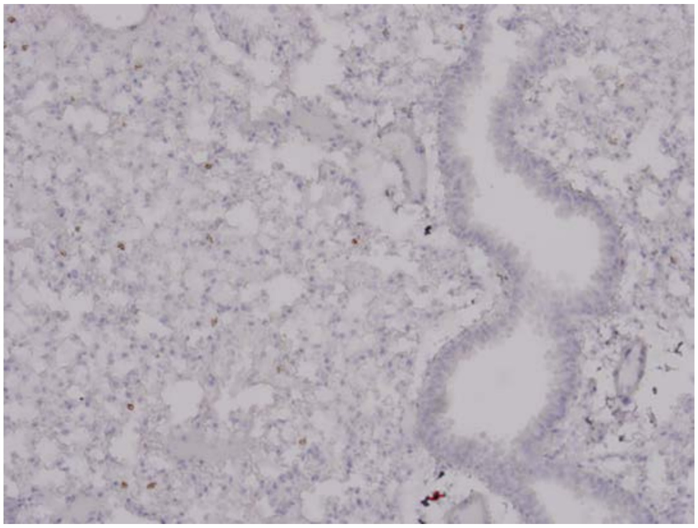

1 CD8$^+$ cells

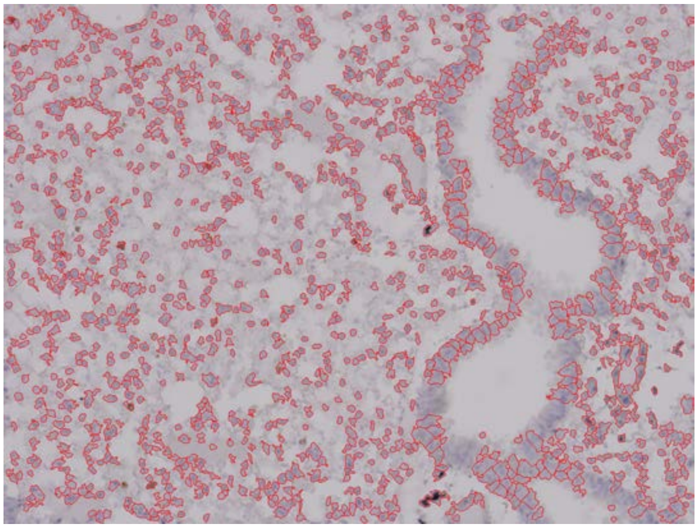

1275 nucleated cells

CD4$^+$

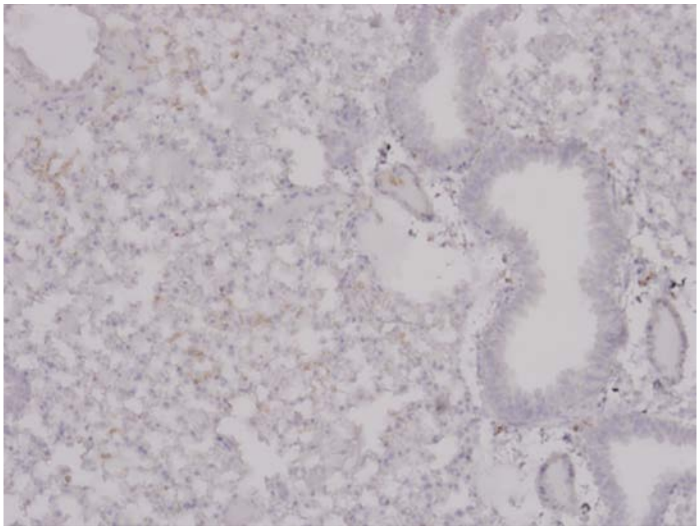

0 CD4$^+$ cells

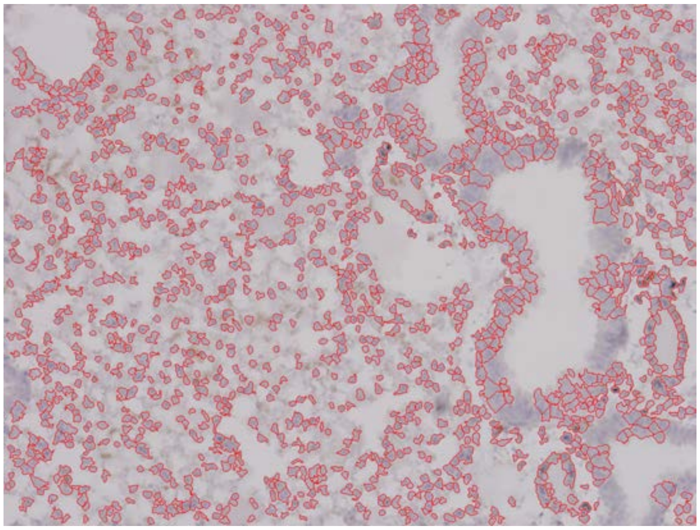

1337 nucleated cells

Supplementary Figure 11
Page 26/30

PBS Lung 2-4
Unchallenged, Female Cohort

CD8$^+$/CD4$^+$ Cell Annotations | Nucleated Cell Annotations

CD8$^+$

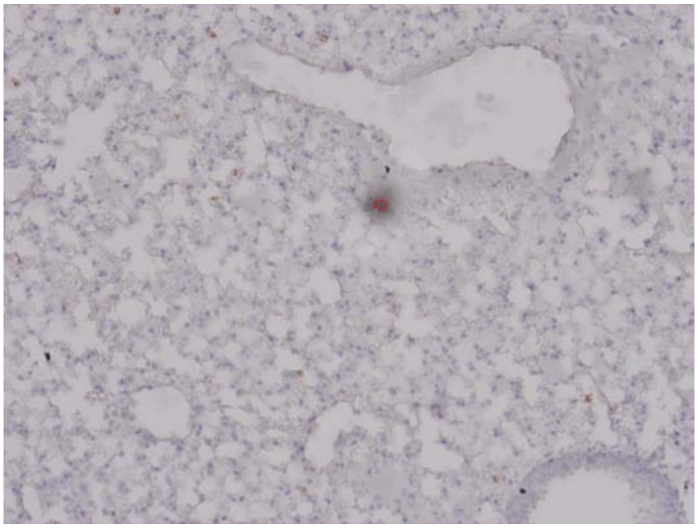

1 CD8$^+$ cells

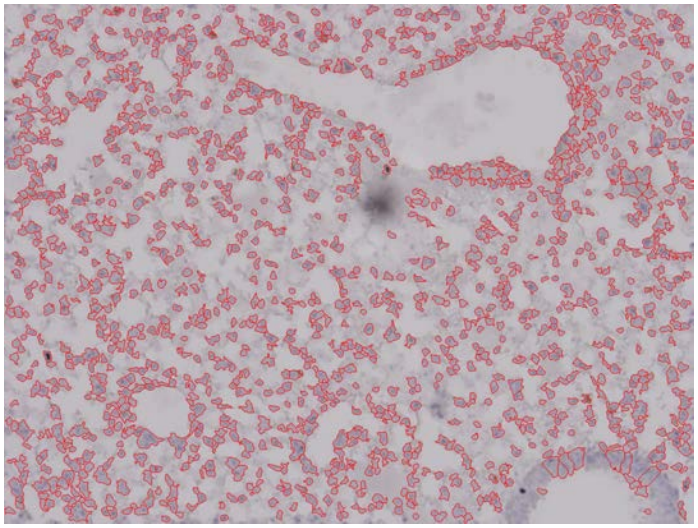

1308 nucleated cells

CD4$^+$

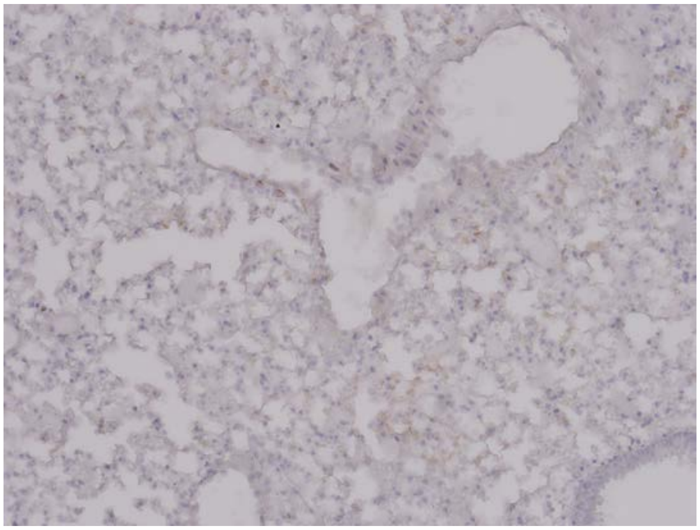

0 CD4$^+$ cells

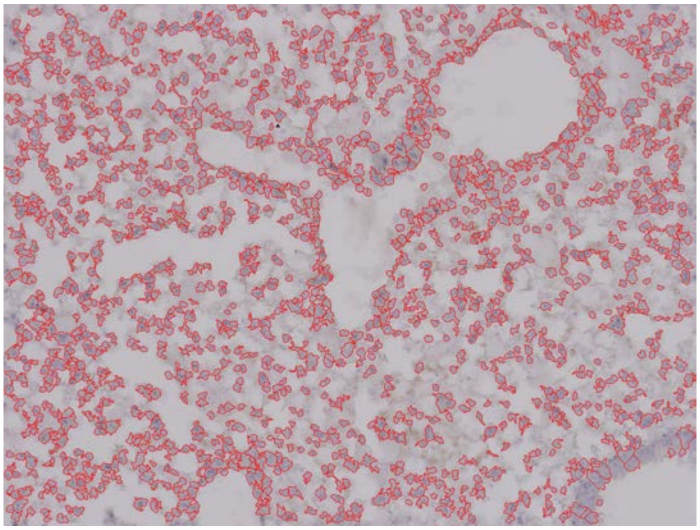

1427 nucleated cells

PBS Lung 3-1
Unchallenged, Female Cohort

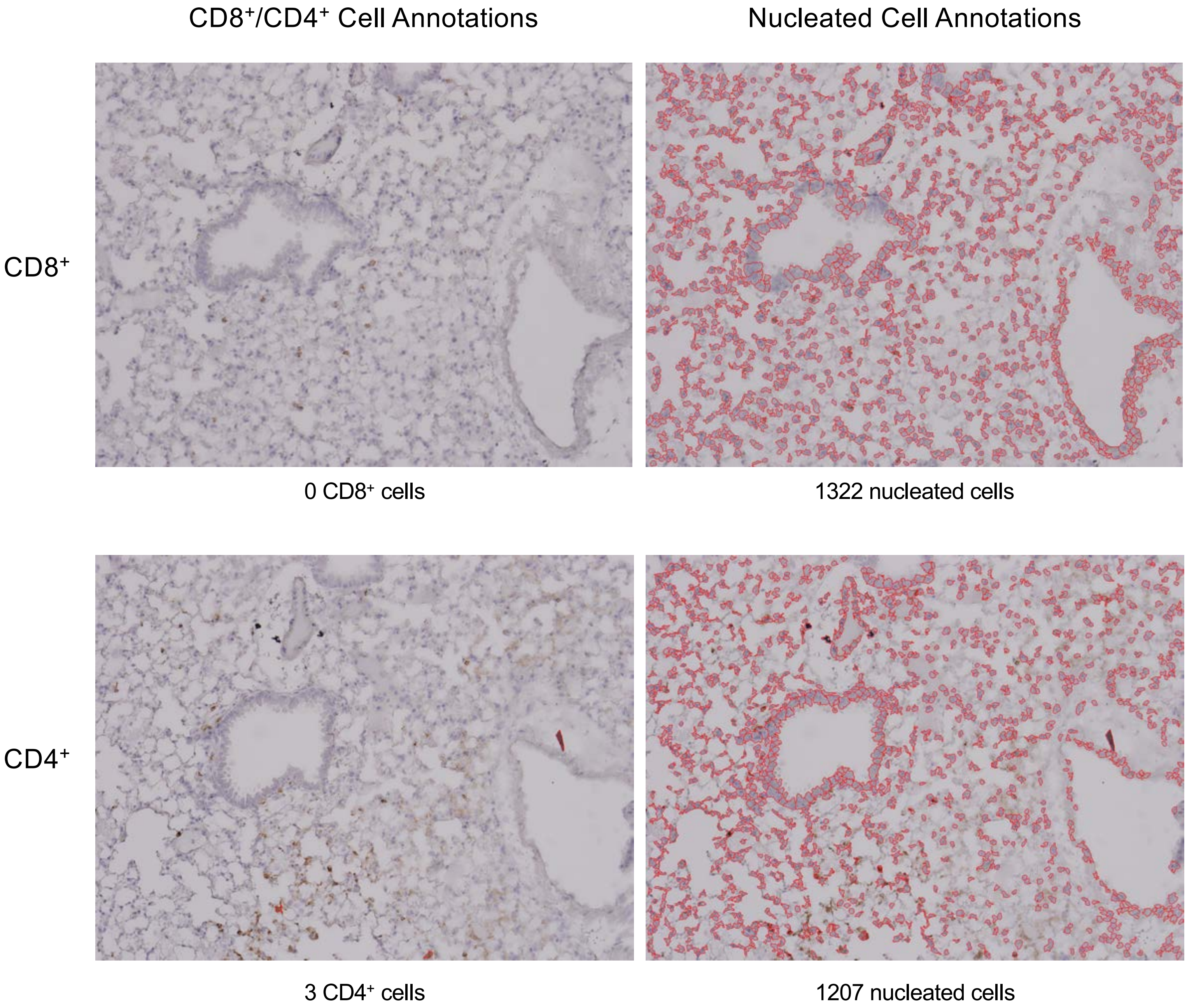

Supplementary Figure 11
Page 28/30

PBS Lung 3-2
Unchallenged, Female Cohort

CD8$^+$/CD4$^+$ Cell Annotations | Nucleated Cell Annotations

CD8$^+$ | CD4$^+$

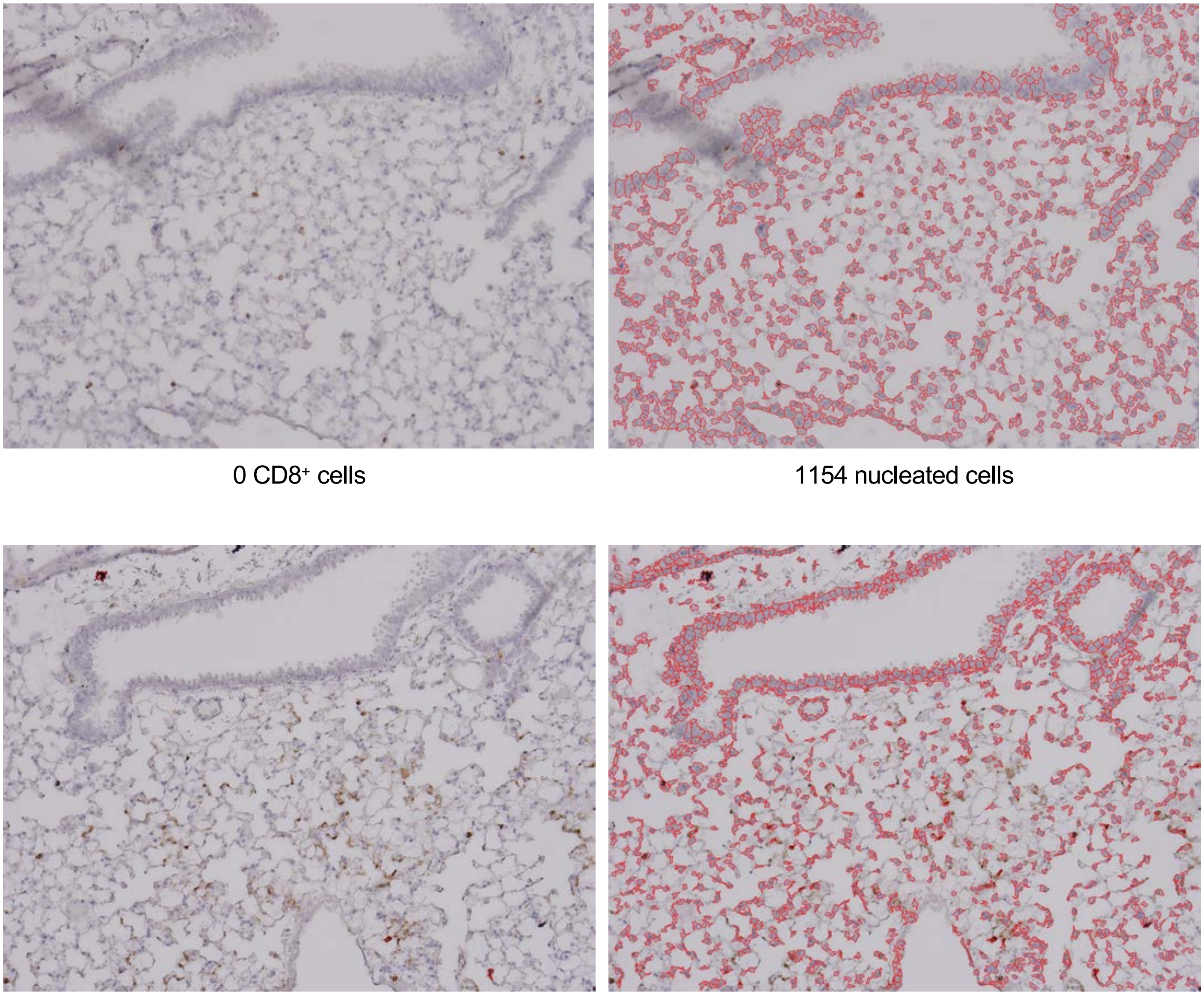

0 CD8$^+$ cells

1154 nucleated cells

3 CD4$^+$ cells

1208 nucleated cells

Supplementary Figure 11

PBS Lung 3-3
Unchallenged, Female Cohort

CD8$^+$/CD4$^+$ Cell Annotations

Nucleated Cell Annotations

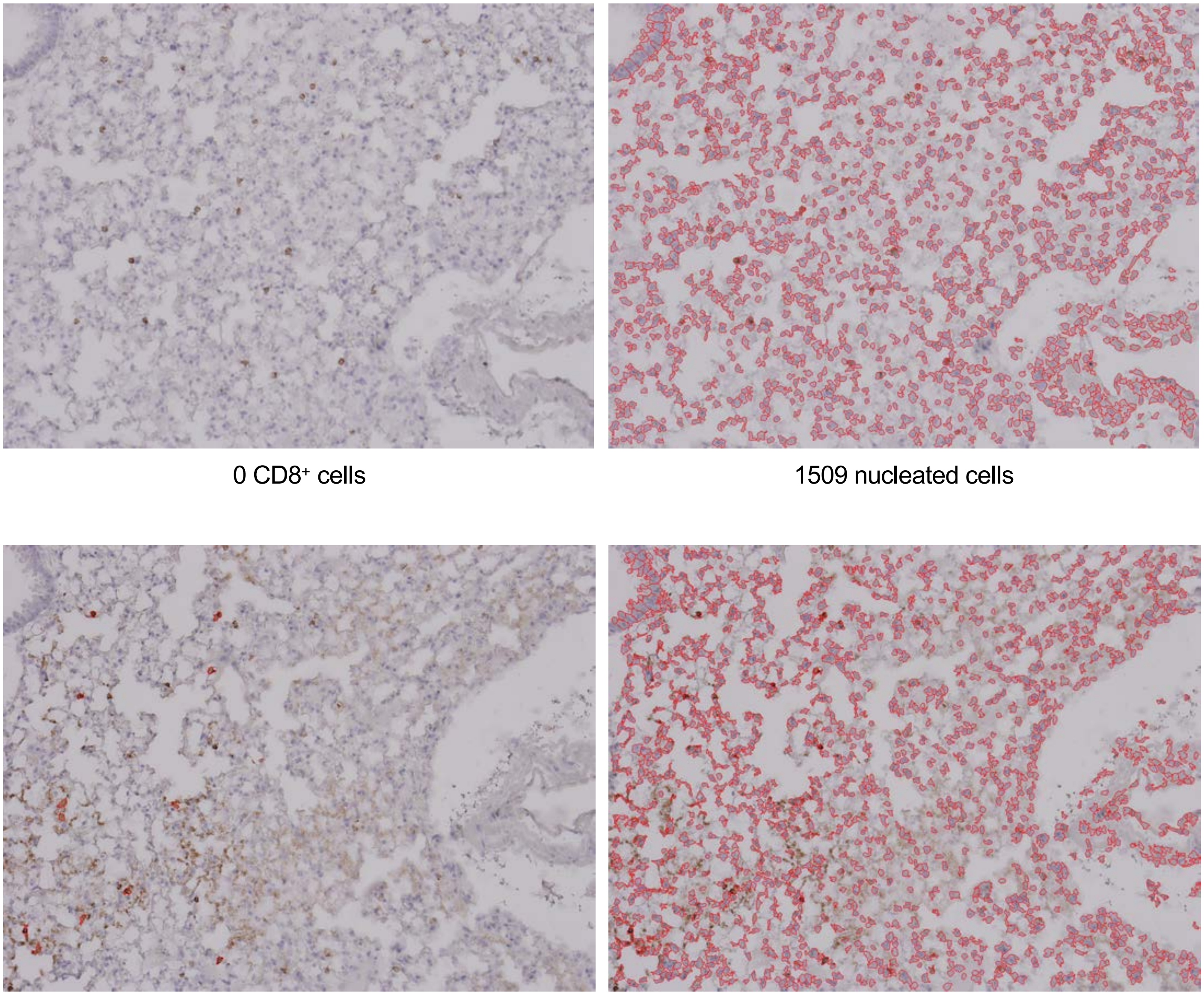

CD8$^+$

0 CD8$^+$ cells

1509 nucleated cells

CD4$^+$

12 CD4$^+$ cells

1402 nucleated cells

Supplementary Figure 11

PBS Lung 3-4
Unchallenged, Female Cohort

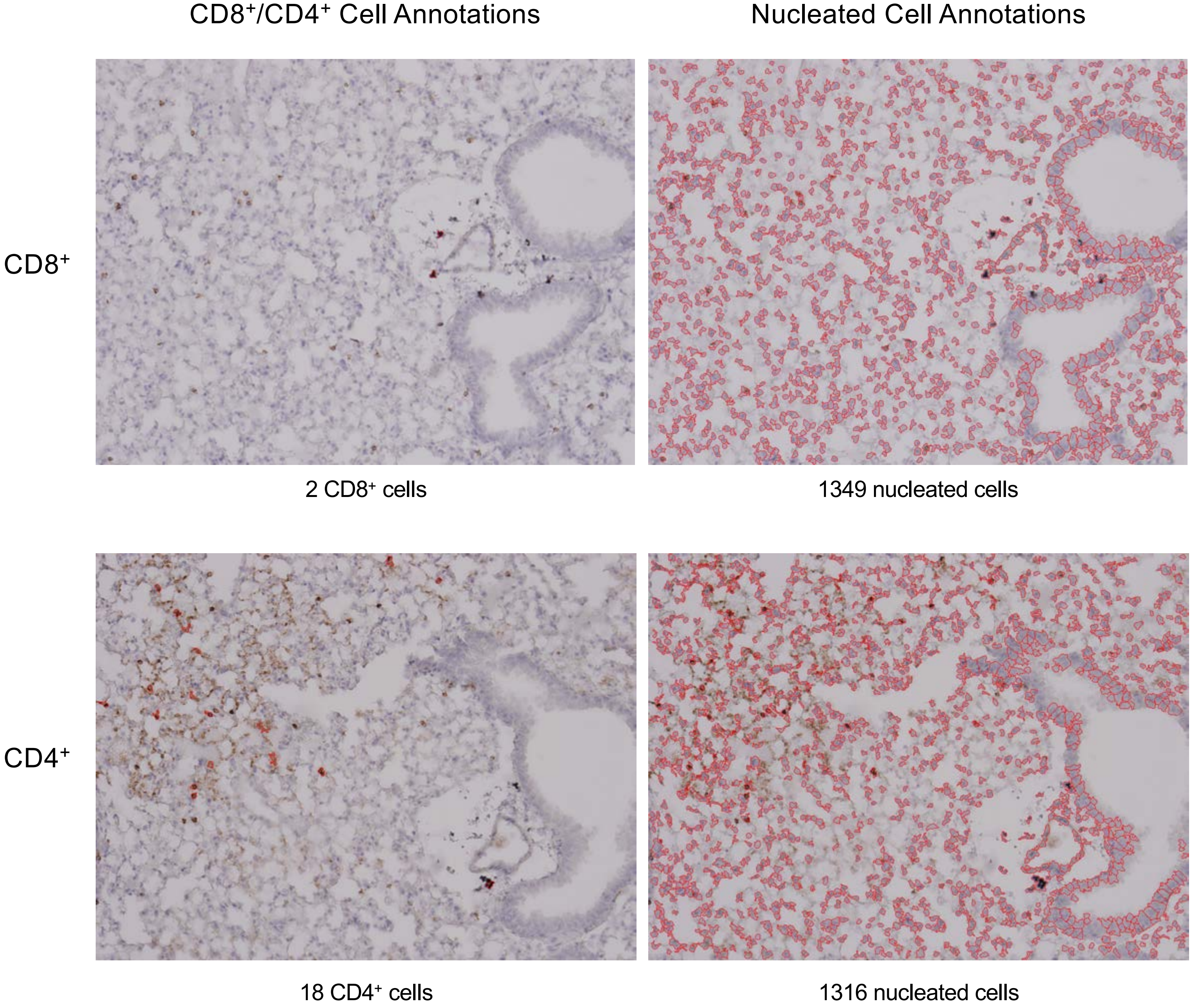

Supplement: Supplementary file 3 [file DataSheet_3.pdf]
